# Circ-0069561 as a novel diagnostic biomarker for progression of diabetic kidney disease

**DOI:** 10.1080/0886022X.2025.2490200

**Published:** 2025-04-22

**Authors:** Chaoyi Chen, Xinran Liu, Sai Zhu, Yukai Wang, Yu Ma, Ziyun Hu, Yonggui Wu, Ling Jiang

**Affiliations:** a Department of Nephropathy, The First Affiliated Hospital of Anhui Medical University, Hefei, China; b Center for Scientific Research, Anhui Medical University, Hefei, China

**Keywords:** Diabetic kidney disease, CircRNAs, transcriptome sequencing, ferroptosis

## Abstract

**Background:**

Circular RNAs (circRNAs) are non-coding RNAs that are key regulators of the initiation and progression of various human diseases. However, the role of circRNAs in diabetic kidney disease (DKD) remains unknown.

**Methods:**

Whole high-throughput RNA sequencing (RNA-seq) was performed on kidney tissues from clinical DKD patients and controls. Circ-0069561 with significantly up-regulated expression level was selected by real-time PCR (RT-PCR) analysis. RT-PCR and fluorescent *in situ* hybridization (FISH) further validated the expression and subcellular localization of circ-0069561 in type 2 diabetic mice and DKD patients. The clinical significance of circ-0069561 in DKD was evaluated. The circRNA-miRNA-ferroptosis associated mRNA network was constructed. The biological function of circ-0069561 in mouse podocyte clone 5 (MPC5) was analyzed.

**Results:**

The top 10 up-regulated circular RNAs were selected by RT-PCR validation, and the results demonstrated a significant elevation in the expression level of circ-0069561. The RT-PCR and FISH results demonstrated that the expression of circ-0069561 was elevated in renal tissues of type 2 diabetic mice and DKD patients, with a predominant localization in glomerulus. The ROC curves showed that circ-0069561 had a good diagnostic value in massive proteinuria (area under the curve = 0.889). Kaplan-Meier analysis showed that high expression of circ-0069561 was associated with an increased risk of primary endpoints. The circRNA-miRNA-mRNA network indicated that ferroptosis might be involved in the pathogenesis of DKD. *In vitro* experiments demonstrated that circ-0069561 aggravated glucose-induced podocyte damage and ferroptosis.

**Conclusion:**

Circ-0069561 has the potential to be an ideal biomarker and therapeutic target for DKD progression.

## 1. Introduction

Diabetic kidney disease (DKD) is one of the most serious microvascular chronic consequences in diabetic patients [1]. Diabetes mellitus (DM) is an international health crisis. According to the prediction of the World Health Organization, diabetes mellitus will affect about 642 million people globally by 2040. Diabetes can involve all important organs in the body, including central nervous complications, peripheral neuropathy, renal disease, cardiovascular disease, various infections, etc, seriously damaging the quality of life of patients [2,3]. DM is classified into two types: type 1 (inadequate the production of insulin from pancreatic β-cells) and type 2 (insulin resistance). Around 40% of DM patients progress to DKD, and 30% to 50% of these cases attributed to T2D [4]. DKD is the main cause of dialysis treatment for end-stage renal disease (ESRD), resulting in significant economic burdens [5]. The molecular pathophysiology of DKD remains unknown. Thus, it’s critical to investigate novel treatment targets and gain a deeper understanding of the molecular mechanism behind DKD.

In eukaryotes, circular RNA (circRNA) is an ubiquitous type of noncoding RNA [6]. CircRNAs exhibit greater stability than linear non-coding RNAs because of their covalent closed-loop structure [7]. Circ_104075 may be used as a biomarker for identifying hepatocellular cancer [8]. Researchers revealed that circRNAs play a role in the onset and progress of different diseases, including metabolic illnesses like diabetes [9,10]. Our earlier study discovered that the absence of circ-0000953 in DM caused podocyte damage and defective autophagy [11]. Peng et al. discovered that renal fibrosis in DM is a result of down-regulation of circRNA_010383 [12]. CircRNA is intimately linked to the progression of numerous disease.

The mechanisms of DKD are complex. Medical researchers have recently become interested in ferroptosis [13]. Ferroptosis is a new kind of planned cell death that requires iron overload and ROS production, in relative to apoptosis and necrosis [14]. Recent evidence suggests that ferroptosis plays a significant role in the development of DKD [15,16].

In this research, whole high-throughput RNA sequencing (RNA-seq) was utilized to explore circRNA expression profiles, and real-time PCR (RT-PCR) analysis was used to select circ-0069561, which had significantly higher expression levels. The clinical data analysis showed that circ-0069561 has a good value in the detection of massive proteinuria and can be used as an indicator of disease progression. The pathophysiology of DKD may involve ferroptosis, according to the circRNA-miRNA-mRNA network. Podocyte damage and ferroptosis brought on by elevated glucose can be lessened by silencing circ-0069561, while overexpression of circ-0069561 aggravates glucose-induced podocyte damage and ferroptosis. This work suggests that circ-0069561 could be a novel therapeutic target and diagnostic biomarker to preventing DM development.

## 2. Materials and methods

### 2.1. Clinical specimens and collection

Biopsy specimens from the kidneys of four people diagnosed with DKD and healthy kidney tissue samples adjacent to tumors from four patients who underwent nephrectomy for renal tumors were collected for RNA-seq and analysis of genomics. From January 2022 to June 2023, 46 volunteers who had received a kidney biopsy diagnosis of DKD were gathered from Anhui Medical University’s First Affiliated Hospital. One criterion for the advancement of UACR was a urine albumin/creatinine ratio (UACR) of ≥ 300 mg/g. Diabetic nephropathy patients were further separated into two groups: DKD1 patients (those with microalbuminuria or negative protein urine) and DKD2 patients (those with macroalbuminuria) with UACR ≥ 300 mg/g. Renal biopsy was also used to harvest kidney tissue from the minimal change disease (MCD) group. With reference to other studies [17,18], we selected MCD as the disease control group mainly based on its unique pathological features. MCD is characterized by the fusion of foot processes in glomerular epithelial cells, but is not accompanied by basement membrane thickening, which is in sharp contrast to the pathological changes of DKD. By using MCD as a control, we were able to more accurately identify the specific pathological mechanisms of DKD and isolate the effects of specific changes. Referring to other studies [17–19], kidney tissue from the normal control (NC) group was extracted after urological surgery, ensuring that the sampling location was > 2 cm from the tumor edge and evaluated by a pathologist. All specimens were verified by histopathology. RNA-seq results were confirmed with RT-PCR in 46 kidney tissues from DKD patients and 12 healthy kidney tissues. Consent for this study was given by the First Affiliated Hospital’s Research Ethics Committee of Anhui Medical University (NO.2022311), which was executed in accordance with the Helsinki Declaration. Everyone who participated signed a consent form.

### 2.2. Chemicals and antibodies

In this research, the following chemicals and antibodies were used: Lipofectamine RNAiMAX Reagent (Invitrogen Life Technologies, 13,778-030) was used for transfection techniques, closely adhering to the manufacturer’s recommendations. Proteintech’s 66009-1-Ig anti-β-actin antibody, 60004-1-Ig anti-GAPDH antibody, Abcam’s ab267377 anti-WT-1 antibody, Abcam’s ab181143 anti-podocin antibody, HUABIO’s ET7111-43 anti-ACSL4 antibody, and HUABIO’s ET1706-45 anti-GPX4 antibody are among the antibodies. Biochemical Assays: Superoxide dismutase (SOD), glutathione (GSH), and malondialdehyde (MDA) were measured using assay kits purchased from Jiancheng (Nanjing, China). Iron content was quantified using an assay kit from Leagene (Beijing, China). The DHE were achieved in accordance with the kits provided by Beyotime (Jiangsu, China).

### 2.3. CircRNA sequencing

RNA-seq was performed by Kang Cheng Bio (Shanghai, China). The total RNA samples were subjected to oligo dT enrichment (rRNA removal), after which the KAPA Stranded RNA-Seq Library Prep Kit (Illumina) was employed to construct the libraries. Further data mining analyses were done through the Kang Cheng Biologicals program.

### 2.4. Inclusion and exclusion criteria

The criteria for including participants in DM group were established as follows [17]: Clinically and pathologically diagnosed type 2 diabetes, aged 18–80 years. Individuals with type 1 diabetes, secondary diabetes, and other diabetes types were not considered eligible; Patients with diabetic ketoacidosis were excluded; Patients with severe infection, pregnancy, other endocrine diseases, connective tissue diseases, malignant tumors, myocardial infarction, and severe liver dysfunction were excluded. This study included two control groups: MCD and NC. Exclusion criteria for these control groups included the following: (1) any type of diabetes; (2) severe heart, liver or renal insufficiency; (3) Urinary tract infection; (4) Pregnancy or breastfeeding. In total, the study comprised 73 participants (NC:MCD:DKD1:DKD2 = 12:15:10:36).

### 2.5. Pathological examination of renal tissue utilized staining techniques and transmission electron microscopy (TEM)

Solarbio Corporation (Beijing, China) provided the following staining kits: hematoxylin and eosin (H&E), periodate-schiff (PAS), Masson, and periodic acid-silver methenamine (PASM). Tissue slices were fixed in formalin, embedded in paraffin, and stained with H&E, PAS, Masson, and PASM according to the manufacturer’s instructions. Renal cortical tissues were fixed in 2.5% glutaraldehyde, dried and embedded, and examined under a transmission electron microscope.

### 2.6. Type 2 diabetes mouse model

The study utilized a db/db mouse model of type 2 diabetes. Male db/db mice and db/m control mice aged 6 weeks were purchased from GemPharmatech (Nanjing, China). These mice were housed in a specific-pathogen-free (SPF) environment with a stable temperature of *22 °C* ± 2 °C and a humidity level of 60%, following a 12-h light-dark cycle. Refer to our previous research [20], the db/m control mice (*n* = 6) and db/db mice were maintained until 8 weeks (*n* = 6), 12 weeks (*n* = 6), 16 weeks (*n* = 5), and 20 weeks (*n* = 6), the mice were randomly placed in cages with 2–3 mice per cage. There was no loss of mice during feeding. For each mouse, three different researchers were involved, and the first researcher was responsible for random grouping and feeding. Two researchers were left to measure and evaluate the results. All animals were treated in compliance with the principles of the Declaration of Helsinki and the ARRIVE recommendations. This research was carried out in accordance with the Declaration of Helsinki and approved by Anhui Medical University’s Animal Research Ethics Committee (NO. 20220759).

### 2.7. Immunohistochemical staining

After deparaffinizing kidney paraffin sections, they underwent high-pressure antigen repair using citric acid solution, hydrogen peroxide incubation for 20 min, goat serum blocking for 40 min, goat serum removal, primary antibody addition, and overnight incubation at 4 °C in a humidified refrigerator. The sections were then cleaned and incubated with the secondary antibody for 30 min at 37 °C. The color development of 3,3′-diaminobenzidine (DAB) was carried out. Hematoxylin was used to stain the cell nuclei. Use a Leica microscope (Bensheim, Germany) to examine the sections. Utilize ImageJ software (NIH, Bethesda, MD, USA) to analyze and quantify the staining.

### 2.8. Cell culture

The Institute of Basic Medical Sciences at the Chinese Academy of Medical Sciences provided the conditionally immortalized mouse podocytes (MPC5). These cells were maintained at 33 °C in RPMI 1640 supplemented with 20 U/mL of mouse recombinant interferon-g (IFN-g) and 10% fetal bovine serum. The podocytes were maintained at 37 °C for seven days without IFN-γ under non-permissive conditions in order to induce differentiation. Professor Lan HY of the Chinese University of Hong Kong kindly provided mouse tubular epithelial cells (mTECs). Vascular endothelial cells (VECs) and mouse mesangial cells (SV40) were obtained from the Chinese Academy of Sciences Cell Bank and cultivated in DMEM supplemented with 5% serum. MPC5 cells were transfected with siRNA or an overexpression plasmid (Hanbio Biotechnology, China) using Lipofectamine RNAiMAX Reagent (Invitrogen) in accordance with the manufacturer’s recommendations. Table S1 lists the siRNA sequences that were employed in this investigation. A negative scrambled siRNA or plasmid (provided by Hanbio Biotechnology, China) was used as the control. The diluted siRNA or plasmid was combined with Lipofectamine RNAiMAX and incubated for 20 min prior to being introduced to the cells in OPTI-MEM medium. Following a six-hour incubation period, the medium was replaced with a complete medium. The treatments employed in this study included high glucose (HG) (30 mM glucose), mannitol, and moderate glucose (MG) (24.5 mM mannitol + 5.5 mM glucose), as well as normal glucose (NG) (5.5 mM glucose).

### 2.8. Fluorescence *in situ* hybridization (FISH)

For the hybridization process, circ-0069561-specific fluorescent dye-labeled nucleic acid probes were employed. First, 4% paraformaldehyde was used to fix the samples, and then hydrochloric alcohol was used to wash them. After ten minutes of air drying at 46 °C, they were put through a five-minute gradient ethanol dehydration process. After that, 1 μL of the probe was mixed with 10 μL of the previously made hybridization buffer. For one and a half hours, the hybridization was carried out in a sealed chamber with washing buffer at 46 °C in the dark. The slides were then incubated for a further half hour at 48 °C once the temperature was raised to that level. Following hybridization, distilled water was used to rinse the slides and an anti-fading solution was used. The Leica microscope (Bensheim, Germany) was used to investigate the location of circ-0069561 expression. Table S2 contains a list of the probe sequences that were employed.

### 2.9. RT-PCR analysis

Total RNA was isolated from human paraffin-embedded tissue sections using the Paraffin Tissue Total RNA Kit (Simgen, Hangzhou, China). Following the manufacturer’s instructions, RNA extraction was carried out using Trizol (Invitrogen) for cellular or renal samples. Mean ± SEM was utilized to display the results, with β-actin serving as an internal control for normalization. Table S3 lists all of the primer sequences used in this investigation.

### 2.10. Western blotting

Cell and kidney tissue samples were lysed using ice-cold radio-immunoprecipitation assay (RIPA) buffer supplemented with protease and phosphatase inhibitors (Beyotime, China). Following denaturation, proteins were separated using SDS-PAGE and transferred to a membrane for further immunoblotting using specific antibodies. The protein bands were identified using an Enhanced ECL Chemiluminescent Substrate Kit (Yeasen, 36222ES76), and images were captured using the Amersham Imager 600 System.

### 2.11. KEGG pathway enrichment analysis and construction of a circRNA-miRNA-mRNA network

We constructed a circRNA-miRNA-mRNA network to study their interactions. Moreover, the FerrDb database was used to identify mRNAs associated with ferroptosis. Ferroptosis-associated mRNAs and differentially expressed mRNAs intersected in the ceRNA network for KEGG pathway analysis of ferroptosis-associated DEGs. CircRNA-miRNA-ferroptosis-associated mRNA networks were then constructed using Cytoscape software (v3.7.1).

### 2.12. Sample size calculation

The sample size was calculated as mentioned above [17]. We divided the patients into four groups: NC, MCD, DKD1, and DKD2. We evaluated the expression level of circ-0069561 by RT-PCR. The sample size was calculated from the mean and SD of circ-0069561 expression in kidney tissue. We set the sample size ratio of NC:MCD:DKD1: DKD2 to 1:1:1:2 and define the Class I error *α* of the hypothesis test to be 0.05 and Class II error *β* to be 0.2. The minimum sample size was calculated by comparing the expression of circ-0069561 in multiple groups in the pre-experiment. The mean and standard deviation of NC, MCD, DKD1 and DKD2 groups were 0.0002 ± 0.00024, 0.001 ± 0.016, 0.0209 ± 0.02924 and 0.05 ± 0.02295, respectively. The minimum sample sizes of the four groups were 5, 5, 5 and 10, respectively. The actual sample size will be expanded as far as possible on this basis to improve the efficiency of the test.

### 2.13. Endpoints

The endpoints are as follows: (1) all-cause mortality, (2) development of ESRD (estimated glomerular filtration rate [eGFR] < 15 mL/min/1.73 m^2^), (3) need for renal replacement therapy, and (4) elevated serum creatinine (2 times higher than baseline). All patients enrolled in the study were followed up by telephone until 1 March 2025.

### 2.14. Statistical analyses

Each experiment was carried out at least three times. For statistical analysis, R (version 4.2.1) and SPSS (version 26.0) were utilized. First, the normality of the data was evaluated using the Shapiro-Wilk test. When describing quantitative data with a normal distribution, the mean ± SD is utilized. One-way analysis of variance was employed for comparisons between several groups, whereas the *t*-test was employed for comparisons between two groups. Group variances and multiple comparisons were evaluated using Tamhane’s T2 and LSD tests, respectively, and Levene’s test for equality of variances. When displaying variables that are not normally distributed, interquartile ranges and medians are utilized. The Kruskal–Wallis *H*-test and the Mann–Whitney *U*-test were used to assess differences between two and three groups, respectively. Pearson’s and Spearman’s correlation coefficients were used to assess the normality of normally and non-normally distributed variables, respectively. Covariates were initially evaluated using univariate regression analysis, and then multivariable logistic regression analysis, in order to develop prediction models. The models were evaluated using receiver operating characteristic (ROC) curves. Kaplan-Meier analysis was used to estimate the Event-free survival curve, and the comparison between different survival curves was made by the log-rank test. Graphing was done using GraphPad Prism (version 8). *p* < 0.05 was set as the significant level.

## 3. Results

### 3.1. Expression profile of circRNA in DKD

**Figure 1.**
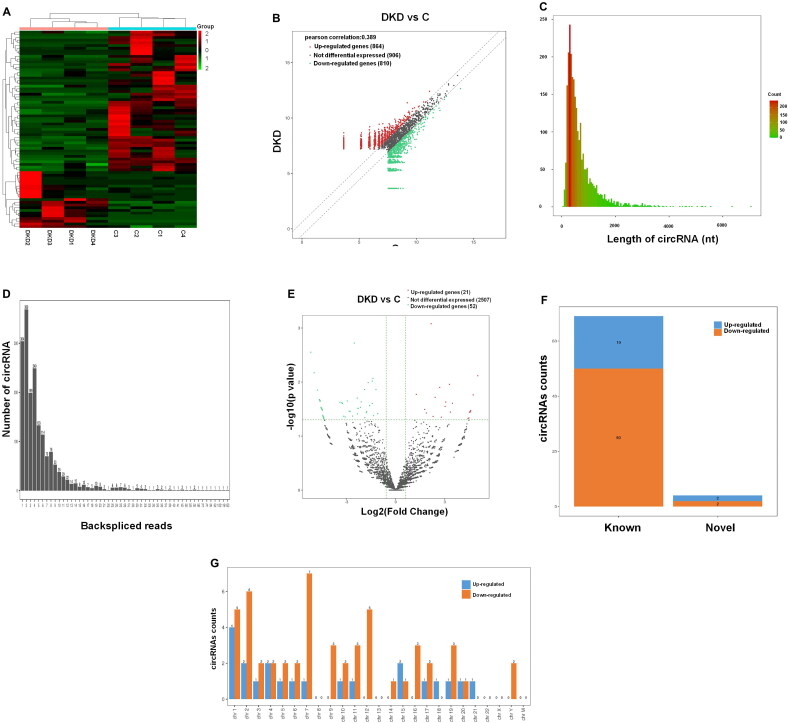
Analysis of differentially expressed circRNA in DKD and NC renal tissues. (A) Heatmap comparing the expression levels of circRNAs in the DKD group (*n* = 4) with the NC control group (*n* = 4). Horizontal axis markers represent DKD and NC samples, and vertical axis markers represent circRNAs expression levels. Expression values are indicated by the color scale. The intensity gradually increases from green (relatively low expression) to red (relatively high expression). Each column represents a tissue sample and each row represents a circRNAs. (B) Scatterplot to assess the distribution of circRNAs between DKD and NC groups. Red dots represent up-regulated circRNAs, green dots represent down-regulated circRNAs, and grey dots represent circRNAs that were not differentially expressed. (C) Length of circRNAs. (D) Reverse shear reads of circRNAs. (E) Volcano plots of differentially expressed circRNAs in the DKD and NC groups. Red dots indicate significantly differentially expressed upregulated circRNAs and green dots indicate significantly differentially expressed downregulated circRNAs. outside the two vertical lines are circRNAs with log2FC absolute value ≥ 0.585, and the horizontal line indicates a p-value of 0.05. (F) Percentage of new and known differentially expressed circRNAs. (G) Location of differentially expressed circRNAs on the position on human chromosomes.

Four patients with diabetic nephropathy and four paracancerous tissues were used for RNA sequencing, with the objective of identifying functional circRNAs involved in the progression of the disease Figure 1(A,B) illustrates the results of the heatmaps and scatter plots, which demonstrate that a total of 2,580 circRNAs were identified, with notable differences observed between the DKD and NC groups. The length and reverse shear reads of the detected circRNAs are presented in Figure 1(C,D) Volcano plots demonstrated that, using log2FC absolute value ≥ 0.585 and *p* Value < 0.05 as the cutoff value, 21 circRNAs were upregulated in diabetic nephropathic tissues, and 52 were downregulated in diabetic nephropathic tissues (Figure 1(E)). Of the 73 identified circRNAs, four were novel discoveries, while 69 were previously reported (Figure 1(F)). These circRNAs were predominantly distributed across autosomes, with two downregulated circRNAs originating from sex chromosomes (Figure 1(G)).

### 3.2. Screening for differentially expressed circRNAs

**Figure 2.**
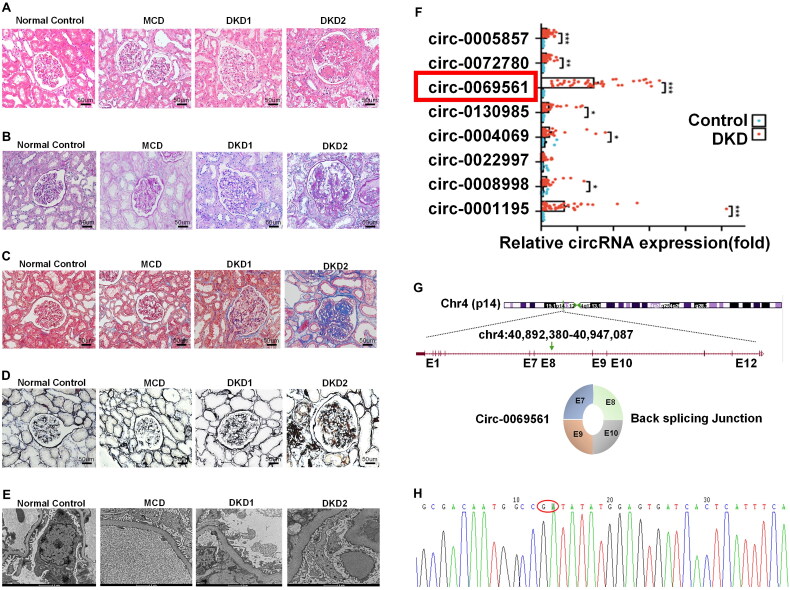
Screening for differentially expressed circRNAs. (A) H&E staining of human kidney. Scale bar = 50 μm. (B) PAS staining of human kidney. Scale bar = 50 μm. (C) MASSON staining of human kidney. Scale bar = 50 μm. (D) PASM staining of human kidney. Scale bar = 50 μm. (E) TEC of human kidney. Scale bar = 2 μm. (F) Relative expression levels of 8 up-regulated circRNAs in renal tissues of 46 DKD patients and 12 NC controls, *p < 0.05, **p < 0.01, ***p < 0.001. (G) Public prediction sites predicted the genomic structure of circ-0069561. (H) Sanger sequencing confirmed the circ-0069561 reverse splice site. H&E: hematoxylin and eosin; PAS: Periodate-Schiff; PASM: periodic acid-silver methenamine; TEM: transmission electron microscopy.

The baseline characteristics of 46 patients with DKD (including 10 patients with microproteinuria DKD and 36 patients with massive proteinuria DKD), 15 patients with MCD, and 12 patients with urological tumors postoperatively are shown in Table 1. There were no statistically significant differences between the groups in terms of age or sex distribution (*p* > 0.05). Additionally, as Table 2 illustrates, DKD patients with UACR ≥ 300 mg/g did not significantly differ from DKD patients with UACR < 300 mg/g in terms of DM, systolic blood pressure (SBP), diastolic blood pressure (DBP), blood urea nitrogen (BUN), serum creatinine (Scr), eGFR, fasting blood glucose (FBG), glycosylated hemoglobin (HbAc1), triglyceride (TG), C-reactive protein (CRP), and urinary-β2-microglobulin (U-β2-MG). Total cholesterol (TC), urinary creatinine (UACR), urinary transferrin (U-TRF), urinary immunoglobulin G (uIgG) and urinary-α1-microglobulin (U-α1-MG) were increased in the DKD2 group compared to the DKD1 group. And albumin (ALB) was decreased in the DKD2 group compared to the DKD1 group. The differences were statistically significant (*p* < 0.05). We performed HE, PAS, Masson and PASM staining on the sections of each group and observed the characteristic changes of DKD under electron microscope. Representative pathological pictures of each group are shown in Figure 2(A–E). A comparative examination of DKD individuals’ pathological data is presented in Table 3. The glomerular lesions, arteriolar hyalinosis and arteriosclerosis of DKD2 group were higher than that of DKD1 (*p* < 0.05), and the two groups’ differences in interstitial inflammation and IFTA were not statistically significant (*p* > 0.05).

**Table 1.** Baseline demographic data of patients expression of renal tissue.

Variables	NC (*n* = 12)	MCD (*n* = 15)	DKD (*n* = 46)		*p* Value
			DKD1 (*n* = 10) (UACR 30–299 mg/g)	DKD2 (*n* = 36) (UACR ≥ 300 mg/g)	
Age (years)	53.00 (42.00, 59.75)	52.00 (32.00, 57.00)	53.00 (48.50, 55.25)	53.00 (42.50, 56.00)	0.765
Sex (M/F)	7/5	9/6	8/2	24/12	0.593
BMI (kg/m^2^)	25.58 ± 3.07	24.44 ± 5.00	26.41 ± 3.91	25.63 ± 3.70	0.648

BMI: body mass index.

**Table 2.** Comparison of clinical data of DKD patients.

Variables	DKD1(*n* = 10) (UACR 30–299 mg/g)	DKD2(*n* = 36) (UACR ≥ 300 mg/g)	*t* (*Z*)	*p* Value
DM duration (Y)	9.00 (2.75, 18.50)	10.00 (3.00, 11.75)	−0.857	0.407
SBP (mmHg)	128.70 ± 7.82	134.22 ± 14.74	−1.135	0.263
DBP (mmHg)	84.30 ± 10.67	83.61 ± 6.01	0.196	0.848
ALB (g/L)	42.85 (40.18, 45.08)	36.55 (28.08, 39.90)	−3.529	<0.001***
BUN (mmol/L)	7.01 (5.79, 8.90)	7.94 (6.11, 11.07)	−1.065	0.298
Scr (μmol/L)	123.70 (63.45, 155.63)	129.05 (92.50, 175.28)	−0.905	0.378
eGFR [ml/min·1.73 m^2^]	54.00 (45.50, 110.50)	50.50 (38.00, 82.25)	−1.186	0.240
FBG (mmol/L)	6.42 (5.33, 8.82)	6.53(4.89, 9.75)	−0.080	0.948
HbAc1 (%)	6.65 (6.15, 8.30)	7.60 (6.53, 8.40)	−0.893	0.378
TC (mmol/L)	4.30 (3.36, 5.11)	5.36 (4.39, 6.34)	−2.423	0.014*
TG (mmol/L)	1.46 (1.00, 3.62)	1.87 (1.41, 2.58)	−0.559	0.591
CRP (mg/L)	2.58 (0.92, 6.66)	1.09 (0.65, 1.66)	−1.505	0.134
UACR (mg/g)	77.00 (32.50, 197.03)	2409.4 (627.50, 4668.30)	−4.714	<0.001***
U-TRF (mg/L)	5.17 (0.58, 9.42)	81.51 (35.17, 151.60)	−4.394	<0.001***
uIgG (mg/L)	11.32 (4.17, 17.59)	154.89 (59.27, 450.66)	−4.581	<0.001***
U-α1-MG (mg/L)	12.67 (6.13, 27.21)	33.02 (16.38, 54.74)	−2.503	<0.001***
U-β2-MG (mg/L)	0.27 (0.08, 1.26)	0.63 (0.21, 8.25)	−1.678	0.096

*Significant level at *p* ≤ 0.05 and *** significant level at *p* ≤ 0.001. DM: diabetes mellitus; SBP: systolic blood pressure; DBP: diastolic blood pressure; ALB: albumin; BUN: blood urea nitrogen; Scr: serum creatinine; eGFR: estimated glomerular filtration rate; FBG: fasting blood glucose; HbAc1: glycosylated hemoglobin; TC: total cholesterol; TG: triglyceride; CRP: C-reactive protein; UACR: urinary creatinine; U-TRF: urinary transferrin; uIgG: urinary immunoglobulin G; U-α1-MG: urinary-α1-microglobulin; U-β2-MG: urinary-β2-microglobulin.

**Table 3.** Comparison of pathological data of DKD patients.

Variables	DKD1(*n* = 10) (UACR 30–299 mg/g)	DKD2(*n* = 36) (UACR ≥ 300 mg/g)	*Z*	*p* Value
Glomerular lesions, *n* (*n*%)			3.207	<0.001***
I	0 (0)	0 (0)		
IIa	9 (90)	12 (33.33)		
IIb	1 (10)	3 (8.33)		
III	0 (0)	14 (38.89)		
IV	0 (0)	7 (19.44)		
IFTA, *n* (*n*%)			1.977	0.096
Score 0	0 (0)	1 (2.78)		
Score 1	5 (50)	6 (16.67)		
Score 2	5 (50)	25 (69.44)		
Score 3	0 (0)	4 (11.11)		
Interstitial inflammation, *n* (*n*%)			1.089	0.646
Score 0	1 (10)	1 (2.78)		
Score 1	9 (90)	34 (94.44)		
Score 2	0 (0)	1 (2.78)		
Arteriolar hyalinosis, *n* (*n*%)			3.143	0.005**
Score 0	1 (10)	1 (2.78)		
Score 1	8 (80)	11 (30.56)		
Score 2	1 (10)	24 (66.67)		
Arteriosclerosis, *n* (*n*%)			4.129	<0.001***
Score 0	2 (20)	0 (0)		
Score 1	8 (80)	11 (30.56)		
Score 2	0 (0)	25 (69.44)		

**Significant level at *p* ≤ 0.01 and *** significant level at *p* ≤ 0.001. IFTA: interstitial fibrosis and tubular atrophy.

Table S4 lists the top 10 circRNAs that are up-regulated. As the GC content and Tm value of the sequences in the vicinity of the chr1:245180543-245180628:+ and circ-0057338 cyclization sites were low and did not meet the requirements of the primer design, we opted to employ RT-PCR in 46 DKD patients and 12 NC controls to verify the expression of the remaining 8 upregulated circRNAs. As illustrated in Figure 2(F), we identified circ-0069561, which exhibited notable distinctions between the DKD and NC groups. To this end, we consulted the circBase database (http://www.circbase.org). We obtained that circ-0069561 is located on chromosome 4:40,892,380–40,947,087. The genomic structure indicated that circ-0069561 was generated by closing shear cyclization of four exons (exons 7, 8, 9 and 10) of the APBB2 locus (Figure 2(G)). Further evidence was provided by Sanger sequencing after PCR, which demonstrated the cyclic structure of circ-0069561. The GA of the cyclization site is depicted in Figure 2(H).

### 3.3. Circ-0069561 expression is elevated in kidney of type 2 diabetic mice and DKD patients

**Figure 3.**
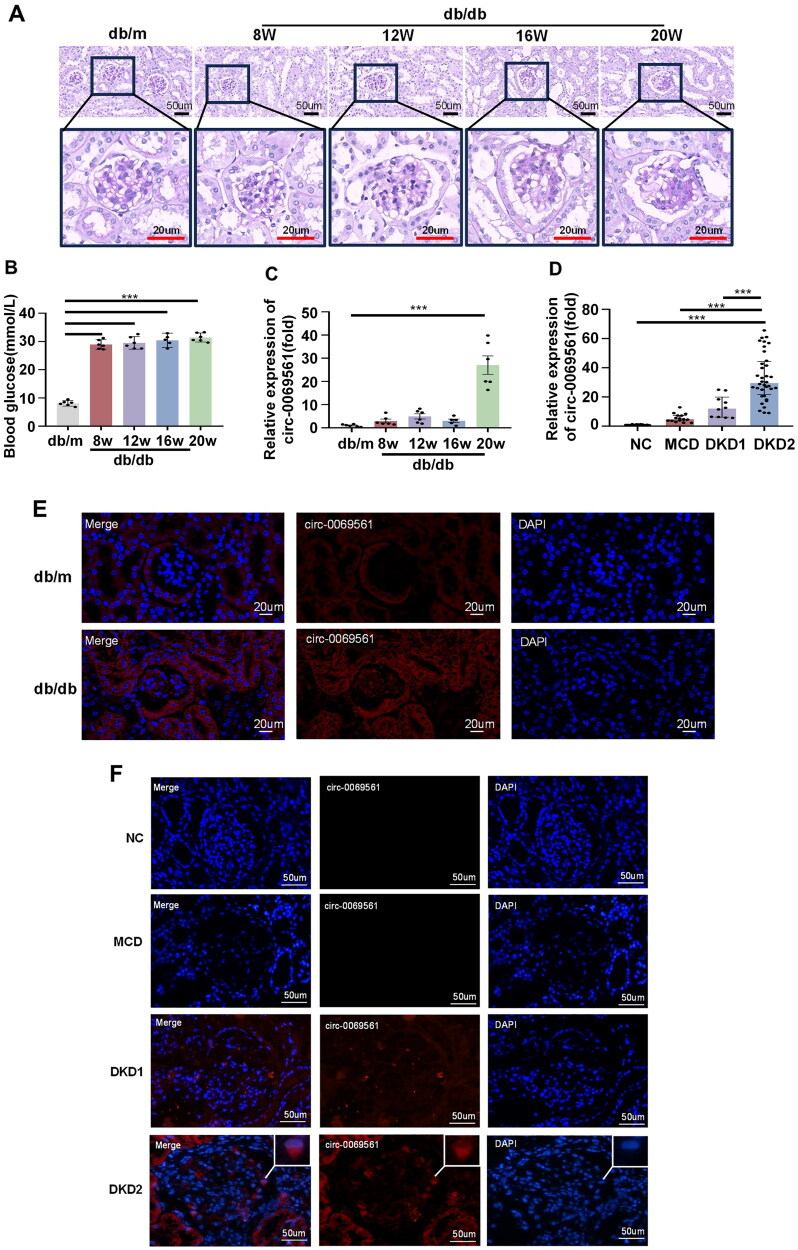
Circ-0069561 expression is up-regulated in type 2 diabetic mice and DKD patients. (A) PAS staining showing typical glomerular structure changes in different groups of mice. Scale bars: black 50 um; red 20 um. (B) Diabetic mice have significantly higher blood glucose, ***p < 0.001. (C) RT-PCR analysis revealed that circ-0069561 expression is up-regulated in renal tissues of db/db, ***p < 0.001. (D) RT-PCR analysis demonstrated that circ-0069561 expression is up-regulated in patients with clinical DKD, ***p < 0.001. (E) FISH experiment revealed that circ-0069561 expression is up-regulated in renal tissues of db/db. Scale bars: 20 um. (F) FISH experiment revealed that the expression level of circ-0069561 was significantly higher in kidney tissues of patients with clinical DKD than that of controls. Scale bars: 50 um.

First, we investigated the expression of circ-0069561 using db/db type 2 diabetic mice. The the glomerular basement membrane thickened, the mesangial matrix expanded, and the diabetic mice’s blood glucose levels were much higher, according to PAS staining (Figure 3(A,B)). In db/db type 2 diabetic mice, we confirmed the expression of circ-0069561 by RT-PCR at various weekly ages. We discovered that circ-0069561 expression was markedly increased (Figure 3(C)). Furthermore, we validated that circ-0069561 expression was greatly enhanced in db/db type 2 diabetic mice and was mostly localized in the glomerulus by performing FISH on kidney tissue samples from these mice (Figure 3(E)). To confirm the expression of circ-0069561 in renal tissues, we applied RT-PCR and FISH in renal tissue samples from 46 patients with DKD (including 10 patients with microproteinuria DKD and 36 patients with massive proteinuria DKD), 15 patients with MCD, and 12 patients with urological tumors postoperatively to circ-0069561 expression was validated, and the results were similar to those of the *in vivo* ex­­periments (Figure 3(D,F)). Next, we focused on the role of circ-0069561 in DKD disease.

### 3.4. Correlation between circ-0069561 expression and clinicopathology of DKD patients

We used RT-PCR to measure the expression level of circ-0069561 in kidney tissue samples from DKD patients and correlated the expression level of hsa_circRNA_0069561 with clinicopathological markers in order to assess the clinical importance of circ-0069561 expression in DKD. The results showed (Table 4) that the expression level of circ-0069561 was positively correlated with serum BUN level, and urine UACR level. Circ-0069561 expression was positively linked with pathological data such as glomerular lesions, interstitial inflammation, arteriolar hyalinosis, and arteriosclerosis (*p* < 0.05).

**Table 4.** Correlation analysis between circ-0069561 level and clinicopathological.

Variables		circ-0069561	
		Correlation coefficient (*r*)	*p* Value
Renal function indices			
	BUN	0.297	0.045*
	Scr	0.049	0.745
	eGFR	−0.034	0.825
Urine test			
	UACR	0.306	0.039*
	U-TRF	0.254	0.088
	uIgG	0.202	0.179
	U-α1-MG	0.072	0.634
	U-β2-MG	0.072	0.635
Other serum indices			
	ALB	−0.113	0.456
	FBG	0.038	0.800
	HbAc1	0.241	0.107
	TC	0.035	0.816
	TG	−0.001	0.993
	CRP	−0.085	0.572
Pathological data			
	Glomerular lesions	0.304	0.040*
	IFTA	0.149	0.323
	Interstitial inflammation	0.376	0.010**
	Arteriolar hyalinosis	0.415	0.004**
	Arteriosclerosis	0.526	<0.001***

*Significant level at *p* ≤ 0.05, **Significant level at *p* ≤ 0.01 and *** significant level at *p* ≤ 0.001. ALB: albumin; BUN: blood urea nitrogen; Scr: serum creatinine; eGFR: estimated glomerular filtration rate; FBG: fasting blood glucose; HbAc1: glycosylated hemoglobin; TC: total cholesterol; TG: triglyceride; CRP: C-reactive protein; UACR: urinary creatinine; U-TRF: urinary transferrin; uIgG: urinary immunoglobulin G; U-α1-MG: urinary-α1-microglobulin; U-β2-MG: urinary-β2-microglobulin; IFTA: interstitial fibrosis and tubular atrophy.

### 3.5. Regression analysis, ROC curve and Kaplan-Meier

**Figure 4.**
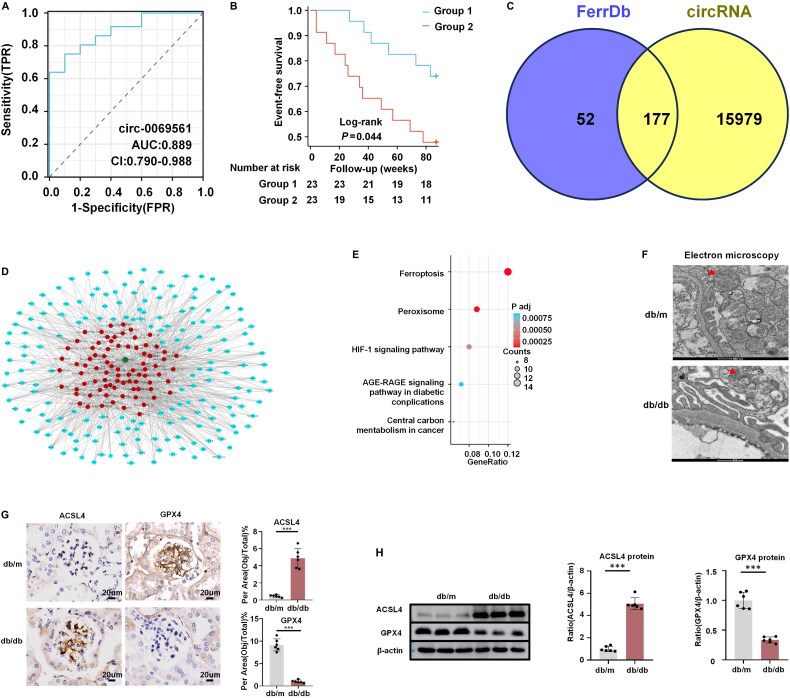
Function of mRNAs in the ceRNA network in DKD and construction of circRNA-miRNA-ferroptosis-related mRNA network. (A) ROC curve analysis of circ-0069561 expression in DKD patients. (B) The Kaplan–Meier analysis for DKD patients with different levels of circ-0069561. (C) Wayne diagram showing intersection analysis of ferroptosis-associated mRNAs with differentially expressed mRNAs in the ceRNA network of circ-0069561. (D) CircRNA-miRNA-ferroptosis-associated mRNA network of circ-0069561. Red circles indicate miRNAs that may be more relevant to DKD. Blue circles represent mRNAs that may be involved in DKD pathogenesis. (E) Significant enrichment of the top 5 KEGG pathways. (F) Representative transmission electron micrographs of podocyte in control and db/db groups. Scale bar: 500 nm (*n* = 6). (G) Immunohistochemistry results showed that ACSL4 levels were increased and GPX4 levels were decreased in the db/db groups. Scale bar: 20 um (*n* = 6), ***p < 0.001. (H) Western blot results also showed that ACSL4 levels were increased and GPX4 levels were decreased in the db/db groups. (*n* = 6), ***p < 0.001.

Binary logistic regression was employed using UACR ≥ 300 mg/g as the grouping criterion [17]. Clinical and pathological data were the initial subjects of univariate regression analysis. A number of indicators were then used in multivariable regression analysis (*p* < 0.05). The levels of circ-0069561 (*β* = 0.956, *p* < 0.05) and ALB (*β* = −0.261, *p* < 0.05) were determined to be possible risk factors using multivariable regression analysis (Table 5). The following prediction model was produced from the results: In (p/1-p) = 0.956 × circ-0069561 − 0.261 × ALB + 8.179. To further explore the diagnostic significance of circ-0069561 in DKD with considerable albuminuria, we created the ROC diagnostic model by considering the kidneys in the DKD2 group as positive samples (*n* = 36) and the kidneys in the DKD1 group as negative samples (*n* = 10) According to the findings, the AUC (0.95 CI) was 0.889 (0.790–0.988). Figure 4(A) and Table S5 both show the ROC curve. They were divided into two groups according to the median expression level of circ-0069561, group1: circ-0069561 < 0.0829 (*n* = 23); group2: circ-0069561 ≥ 0.0829 (*n* = 23). Survival curves for group1 and group2 plotted using Kaplan-Meier analysis, Figure 4(B) showed that high expression of circ-0069561 was associated with an increased risk of primary endpoints, including end-stage renal failure, kidney replacement therapy, doubling of serum creatinine levels, or death. The difference between the two groups was statistically significant (*HR* 2.630, 95%CI 1.035–6.684, *p* = 0.044).

**Table 5.** Binary logistic regression analysis.

Variables	Univariate analysis		Multivariable analysis	
	*p*	*OR* (95% CI)	*p*	*OR* (95% CI)
circ-0069561^a^	0.004**	1.382–5.426	0.013*	1.220–5.542
ALB	0.009**	0.583–0.925	0.023*	0.615–0.964
TC	0.026*	1.121–6.020	——	——
U-TRF	0.014*	1.040–1.402	——	——
uIgG	0.031*	1.014–1.352	——	——
U-α1-MG	0.031*	1.014–1.352	——	——
Glomerular lesions	0.022*	1.320–34.804	——	——
Arteriolar hyalinosis	0.009**	1.668–37.539	——	——

*Significant level at *p* ≤ 0.05 and ** significant level at *p* ≤ 0.01. Circ-0069561^a^ = circ-0069561*50 for inclusion in binary logistics regression; ALB: albumin; TC: total cholesterol; U-TRF: urinary transferrin; uIgG: urinary immunoglobulin G; U-α1-MG: urinary-α1-microglobulin.

### 3.6. Function of mRNAs in the ceRNA network in DKD and construction of circRNA-miRNA-ferroptosis-related mRNA network

The circRNA-miRNA-mRNA network of circ-0069561 was built. FerrDb is a publicly accessible database that provides a comprehensive overview of genes associated with ferroptosis. This includes genes that induce, suppress, or regulate ferroptosis, as well as genes that act as markers for ferroptosis. The ferroptosis-associated mRNAs were obtained by downloading the ferroptosis dataset and overlapping it with the differentially expressed mRNAs in the ceRNA network. This resulted in the identification of 177 DEGs associated with ferroptosis, as illustrated in a shared Venn diagram (Figure 4(C)). A circRNA-miRNA-ferroptosis-related mRNA network was built. The network prediction (Figure 4(D)) revealed that some genes associated with ferroptosis, including ACSL4, ALOX12, ALOX15, and others, were closely linked to the biological function of circ-0069561. The ferroptosis-associated DEGs were subjected to KEGG pathway analysis in order to investigate the role of 177 mRNAs in the ceRNA network. The KEGG pathway analysis in Table S6 and Figure 4(E) indicated that ferroptosis may be associated with the pathogenesis of DKD. Our findings revealed that in diabetic mice, podocyte mitochondria exhibited contraction, an increase in mitochondrial membrane density, and alterations resulting from ferroptosis-induced cell death, as observed through transmission electron microscopy (Figure 4(F)). Furthermore, we found that diabetic mice had higher expression levels of ACSL4 and lower expression levels of GPX4 (Figure 4(G–H)). The results of the *in vivo* investigations suggest that ferroptosis may contribute to the pathogenesis of DKD.

### 3.7. Significant correlation between circ-0069561 expression levels and levels of podocyte injury and ferroptosis in DKD group

**Figure 5.**
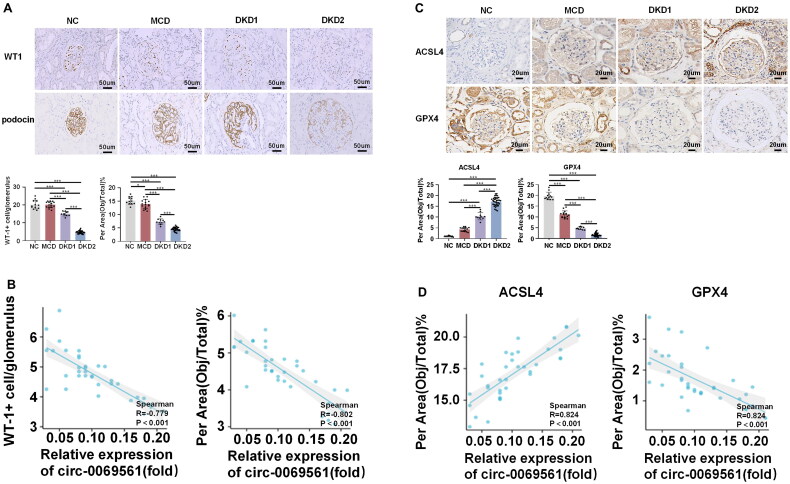
Significant correlation between circ-0069561 expression levels and levels of podocyte injury and ferroptosis in DKD group with massive proteinuria. (A) Immunohistochemistry staining of glomerulus WT1 and podocin. Scale bar: 50 um, *p < 0.05, ***p < 0.001. **(**B) Correlation analysis between circ-0069561 expression level and levels of podocyte injury in DKD group with massive proteinuria. **(**C) Immunohistochemistry staining of glomerulus ACSL4 and GPX4. Scale bar: 20 um, ***p < 0.001. **(**D) Correlation analysis between circ-0069561 expression level and levels of ferroptosis in DKD group with massive proteinuria.

To learn more about the function of circ-0069561 in DKD, we applied immunohistochemistry to validate the expression of markers of podocyte injury and key proteins of ferroptosis in renal tissues from 46 patients with DKD (including 10 patients with microproteinuria DKD and 36 patients with massive proteinuria DKD), 15 patients with MCD, and 12 patients with urological tumors postoperatively. Immunohistochemical findings showed that the glomerulus of DKD patients had considerably lower levels of WT1 and podocin protein than those of the NC and MCD groups; DKD patients with extensive proteinuria had the lowest expression levels (Figure 5(A)). The results in Figure 5(B) showed a significant correlation between circ-0069561 expression levels and the level of podocyte injury in the DKD group with massive proteinuria. In contrast, the glomerulus of DKD patients showed a significantly higher level of ACSL4 protein and a significantly lower level of GPX4 protein when compared to the NC and MCD groups. The expression alterations were especially noticeable in DKD patients with massive proteinuria (Figure 5(C)). Ferroptosis levels and circ-0069561 expression levels had a significant relationship in the mass proteinuria DKD group, according to the results in Figure 5(D). Therefore, we speculated that circ-0069561 may contribute to the development of DKD by inducing ferroptosis in podocytes.

### 3.8. Silencing circRNA-0069561 attenuates high glucose-induced podocyte damage and ferroptosis

**Figure 6.**
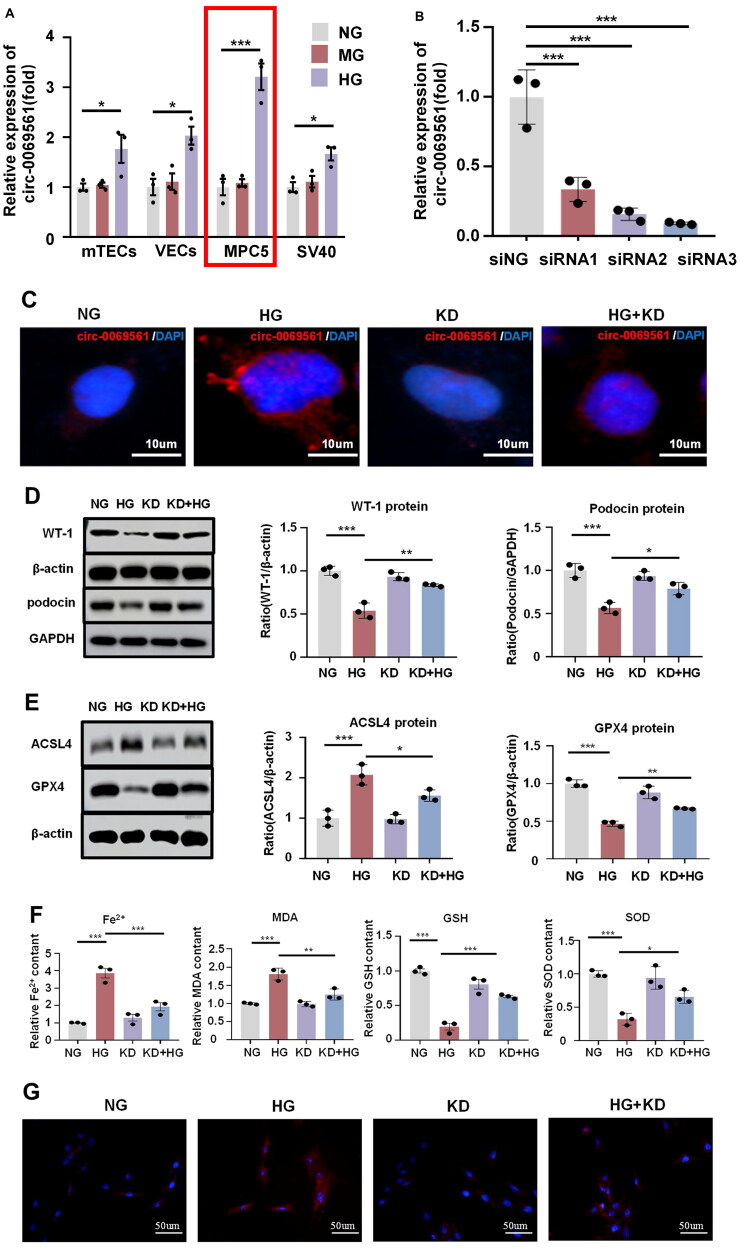
Silencing circRNA-0069561 attenuates high glucose-induced podocyte damage and ferroptosis. (A) Real-time PCR showed that circ-0069561 expression was significantly elevated in high glucose-induced MPC5. mTEC: mouse tubular epithelial cells; vascular endothelial cells: VECs; MPC5: mouse podocyte clone 5; SV40-MES13: mouse glomerulus mesangial cells, *p < 0.05, ***p < 0.001. **(**B) Real-time PCR screening of circ-0069561 siRNA sequences for the best knockdown effect, ***p < 0.001. **(**C) The expression level of circ-0069561 was verified by FISH experiment. Scale: 10 μm, ***p < 0.001. **(**D) Western blot and quantification showed that silencing circ-0069561 attenuated high glucose-induced loss of the podocyte marker proteins WT1 and podocin, *p < 0.05, **p < 0.01, ***p < 0.001. **(**E) Western blot and quantification showed that silencing circ-0069561 attenuated high glucose-induced ferroptosis levels in podocytes, *p < 0.05, ***p < 0.001. **(**F) Fe^2+^, MDA, GSH and SOD level in each group, *p < 0.05, **p < 0.01, ***p < 0.001. (G) Fluorescent probe DHE staining to detect ROS levels in each group of MPC5 cells. Scale bar: 50 um.

The expression level of circ-0069561 was verified in high glucose-induced mTECs, VECs, MPC5, and SV40, respectively, and it was found that circ-0069561 was significantly enriched in podocytes (Figure 6(A)). To further elucidate its function, we constructed an *in vitro* model of podocyte circ-0069561 knockdown (Figure 6(B,C)). The knockdown of circ-0069561 was observed to restore the loss of the podocyte markers WT-1 and podocin that was induced by high glucose (Figure 6(D)). Additionally, it was shown that circ-0069561 knockdown prevented the up-regulation of ACSL4 and restored the down-regulation of GPX4 in podocytes caused by high glucose (Figure 6(E)). To further elucidate the impact of circ-0069561 knockdown on high glucose-induced iron-dependent cell death in podocytes, we examined the levels of Fe^2+^, MDA, GSH, and SOD in each group. Knocking down circ-0069561 reduced Fe^2+^ and MDA buildup in high glucose-induced podocytes while improving GSH and SOD downregulation (Figure 6(F)). DHE staining revealed that the knockdown of circ-0069561 alleviated the accumulation of ROS in high glucose-induced podocytes (Figure 6(G)). In conclusion, the results demonstrate that the knockdown of circ-0069561 mitigates high glucose-induced podocyte injury and ferroptosis.

### 3.9. Overexpression of circRNA-0069561 aggravates high glucose-induced podocyte damage and ferroptosis

**Figure 7.**
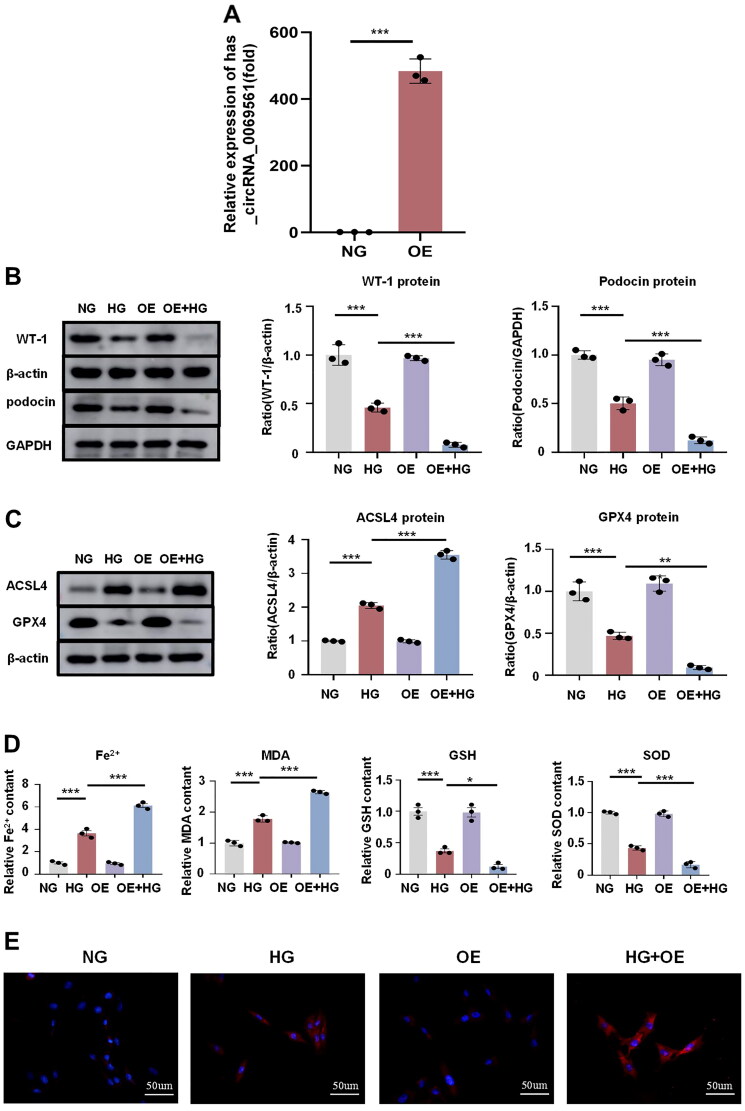
Overexpression of circRNA-0069561 aggravates high glucose-induced podocyte damage and ferroptosis. (A) The overexpression of circ-0069561 was verified by Real-time PCR, ***p < 0.001. (B) Western blot and quantitative analysis showed that overexpression of circ-0069561 aggravated high glucose-induced loss of the podocyte marker proteins WT1 and podocin, ***p < 0.001. (C) Western blot and quantification showed that overexpression of circ-0069561 aggravated high glucose-induced ferroptosis levels in podocytes, **p < 0.01, ***p < 0.001. (D) Fe^2+^, MDA, GSH and SOD level in each group, *p < 0.05, ***p < 0.001. (E) Fluorescent probe DHE staining to detect ROS levels in each group of MPC5 cells. Scale bar: 50 um.

To further elucidate its function, we constructed an *in vitro* model of the overexpression of circ-0069561 in podocyte (Figure 7(A)). We found that overexpression of circ-0069561 aggravated the loss of the podocyte markers WT-1 and podocin that was induced by high glucose (Figure 7(B)). In addition, our results showed that overexpression of circ-0069561 aggravated the up-regulation of ACSL4 and down-regulation of GPX4 in podocytes caused by high glucose (Figure 7(C)). To further elucidate the impact of overexpression of circ-0069561 on high glucose-induced iron-dependent cell death in podocytes, we examined the levels of Fe^2+^, MDA, GSH, and SOD in each group. Overexpression of circ-0069561 aggravated Fe^2+^ and MDA buildup in high glucose-induced podocytes and the downregulation of GSH and SOD (Figure 7(D)). DHE staining showed that overexpression of circ-0069561 increased ROS accumulation in high glucose-induced podocytes (Figure 7(E)).

## 4. Discussion

Chronic kidney disease (CKD) is an important and prevalent complication in patients with T2D [21] and has a significant impact on the global burden [22]. CircRNAs are novel non-coding RNA molecules that have received increasing attention [23]. CircRNAs have been identified with the beginning and progression of kidney disease [24]. In this study, we employed RNA-seq to look for circRNAs that were expressed differently in renal tissues from DKD and NC. Subsequently, circ-0069561, which exhibited markedly altered expression levels, was subjected to screening. Circ-0069561 expression levels correlate with DKD disease severity. Furthermore, podocyte damage and ferroptosis brought on by elevated glucose can be lessened by silencing circ-0069561, while overexpression of circ-0069561 aggravates glucose-induced podocyte damage and ferroptosis. Our findings indicate that circ-0069561 may toward the progress of DKD by inducing ferroptosis in podocytes. This implies that circ-0069561 might be a viable treatment option for DKD, which would have important ramifications for the clinical diagnosis and treatment of DKD.

Firstly, we explored the expression profile of circRNAs in renal tissues of patients with type 2 diabetic nephropathy by RNA-seq and screened for the significantly up-regulated circ-0069561. According to the findings of numerous studies, circRNAs may be used as new biomarkers for cancer and other human disorders [25,26] and heart disease [27]. The function of circRNAs as biomarkers in renal disease has been attempted to be clarified in the past few years. Cao et al. discovered a correlation between the degree of renal fibrosis and urinary exosomal circ_0036649 expression [28]. Another study analyzed RNA-seq in the IMN and HC groups and found that An novel diagnostic biomarker for idiopathic membranous nephropathy was urine exosomal circ_0001250 [29]. A total of 2,580 circRNAs were identified by RNA-seq in this study. Using log2FC absolute value ≥ 0.585 and *P*-value < 0.05 as the thresholds, 21 circRNAs were up-regulated in diabetic nephropathy tissues and 52 were down-regulated in diabetic nephropathy tissues. 10 up-regulated circRNAs were chosen for RT-PCR validation in 46 DKD patients with 12 NC kidney tissues, and the results were in high agreement with the RNA-seq data, which validated the reliability of RNA-Seq results. Meanwhile, we identified circ-0069561, which showed significant difference between DKD and NC groups. To this end, we consulted the circBase database (http://www.circbase.org). We obtained that circ-0069561 is located on chromosome 4:40,892,380–40,947,087. The genomic structure indicated that circ-0069561 was generated by closing shear cyclization of four exons (exons 7, 8, 9 and 10) of the APBB2 locus. Further evidence was provided by Sanger sequencing after PCR, which demonstrated the cyclic structure of circ-0069561.

The current investigation found that both diabetic mouse models and DKD patients have increased expression of circ-0069561. A correlation analysis of clinical trials demonstrated a positive correlation between the expression of circ-0069561 in DKD patients and UACR levels. It is established that a high level of proteinuria represents a significant clinical risk factor for a rapid decline in estimated eGFR and can be used to predict the progress of DKD [30]. Megumi Oshima et al. found that STING activation causes proteinuria, which assists with the development and progress of DKD [31]. We used multiple regression analysis to confirm the clinical significance of circ-0069561 in DKD and discovered that it was an independent risk factor for excessive albuminuria in DKD. The ROC curves demonstrated that circ-0069561 had a high detection value for massive proteinuria. Kaplan-Meier analysis showed that high expression of circ-0069561 was associated with an increased risk of primary endpoints, including end-stage renal failure, kidney replacement therapy, doubling of serum creatinine levels, or death. Therefore, we conclude circ-0069561 that it is associated with the severity of DKD disease and proteinuria.

The significant significance of ferroptosis in DKD was examined in the present study. Ferroptosis is an innovative type of controlled cell death [14]. Yi-Chun Tsai, et al. used RNA-seq to identify ferroptosis as an important pathophysiological mechanism in early DKD [15]. Another study found that tRF3-IleAAT inhibited ferroptosis in diabetic kidney disease mice [16]. Ferroptosis may be a key factor in the development of DKD, according to mounting data. We built a circRNA-miRNA-ferroptosis-related mRNA network in this research and discovered that the biological activity of circ-0069561 was tightly linked to several ferroptosis-related genes, including ACSL4, ALOX12, ALOX15, etc. The KEGG pathway investigation suggests that ferroptosis may be involved in the pathophysiology of DKD. Ferroptosis may be linked to the pathophysiology of DKD, as our work revealed elevated ferroptosis levels in diabetic mice.

Subsequently, we investigated the relationship between circ-0069561 expression levels and the extent of podocyte damage and ferroptosis in patients with DKD. Podocytes are terminally differentiated cells that are unable to proliferate, and podocyte dysfunction in DKD results in proteinuria [32]. We discovered a strong relationship between the degree of podocyte damage in DKD patients and the expression levels of circ-0069561. The lipid metabolism-related gene ACSL4 is a key driver gene for ferroptosis [33]. Lipid oxidation is efficiently inhibited by GPX4, an antioxidant enzyme and structural protein. It has been found to be a vital ferroptosis regulator that influences lipid and amino acid metabolism [34]. Ferroptosis was linked to STZ-induced kidney damage in type 1 diabetic mice and db/db animals [35]. Kim et al. discovered that kidney biopsy samples from diabetic individuals had considerably lower expression levels of SLC7A11 and GPX4 [36]. High fructose also induces ferroptosis in podocytes, ultimately leading to glomerular injury [37]. According of Zhang et al. reducing ferroptosis could stop podocyte damage brought on by elevated glucose [38]. Ferroptosis reduction may therefore be a therapy option for DKD, as there is mounting evidence that ferroptosis contributes to the development of DKD [39]. Ferroptosis levels in DKD patients were shown to be significantly correlated with circ-0069561 expression levels in this investigation. Furthermore, *in vitro* experiments demonstrated that circ-0069561 was significantly enriched in podocytes. To further investigate the role of circ-0069561, we developed *in vitro* models by knocking down and overexpressing circ-0069561 in podocytes. The results showed that silencing circ-0069561 reduces glucose-induced podocyte damage and ferroptosis, while its overexpression aggravates these effects. Therefore, we speculated that circ-0069561 may contribute to the development of DKD by inducing ferroptosis in podocytes.

Despite being instructive, the current results could be constrained by a number of reasons. First off, in order to fully evaluate the clinical utility of circ-0069561 in DKD, additional sample size expansion is required. The patient sample size was quite limited and the follow-up time was short in this study. In addition, this study mainly focused on the clinical relevance of circ-0069561 in DKD and its preliminary verification at the cellular level. The molecular mechanism between circ-0069561 and ferroptosis has not yet been fully elucidated, and we plan to further explore its molecular mechanism in future studies and verify the possible pathway of research through *in vivo* experiments.

## 5. Conclusion

In conclusion, we found that circ-0069561 is highly expressed in DKD. Circ-0069561 expression levels correlate with DKD disease severity and proteinuria, and circ-0069561 may cause podocytes to undergo ferroptosis, which could aid in the progression of DKD. The results of this study can be used to better understand diabetic diabetes and uncover new targets for the disease’s prevention and treatment.

## Supplementary Material

Figure 4C.JPG

Figure 3C.JPG

Figure 6D.JPG

Figure 1G.JPG

Figure 7A.JPG

Figure 2E.JPG

Figure 1E.JPG

Figure 2F.JPG

Figure 7B.JPG

Figure 2H.JPG

Figure 4F.JPG

Figure 6G.JPG

Figure 6A.JPG

Figure 4D.JPG

Figure 6C.JPG

Figure 1A.JPG

Figure 4H.JPG

Figure 1C.JPG

Figure 1D.JPG

Figure 4B.JPG

Figure 5D.JPG

Figure 7C.JPG

Figure 1F.JPG

Figure 3B.JPG

Figure 4G.JPG

Figure 6E.JPG

Figure 3E.JPG

Figure 2D.JPG

Figure 3F.JPG

Figure 2C.JPG

Figure 1B.JPG

Figure 2A.JPG

Figure 4E.JPG

Figure 6F.JPG

Figure 2G.JPG

Figure 7D.JPG

Figure 6B.JPG

Figure legends.doc

Figure 4A.JPG

Figure 3A.JPG

Figure 5B.JPG

Figure 2B.JPG

Figure 3D.JPG

Figure 5C.JPG

Supplementary Tables.doc

Figure 5A.JPG

Figure 7E.JPG

## Data Availability

Readers can access the data underlying the study’s findings by contacting the corresponding author.
