# Supplementary figures and images for: Circ-0069561 as a novel diagnostic biomarker for progression of diabetic kidney disease
Source: Ren Fail. 2025 Apr 22;47(1):2490200. doi: 10.1080/0886022X.2025.2490200 (PMC12016256; doi:10.1080/0886022X.2025.2490200)

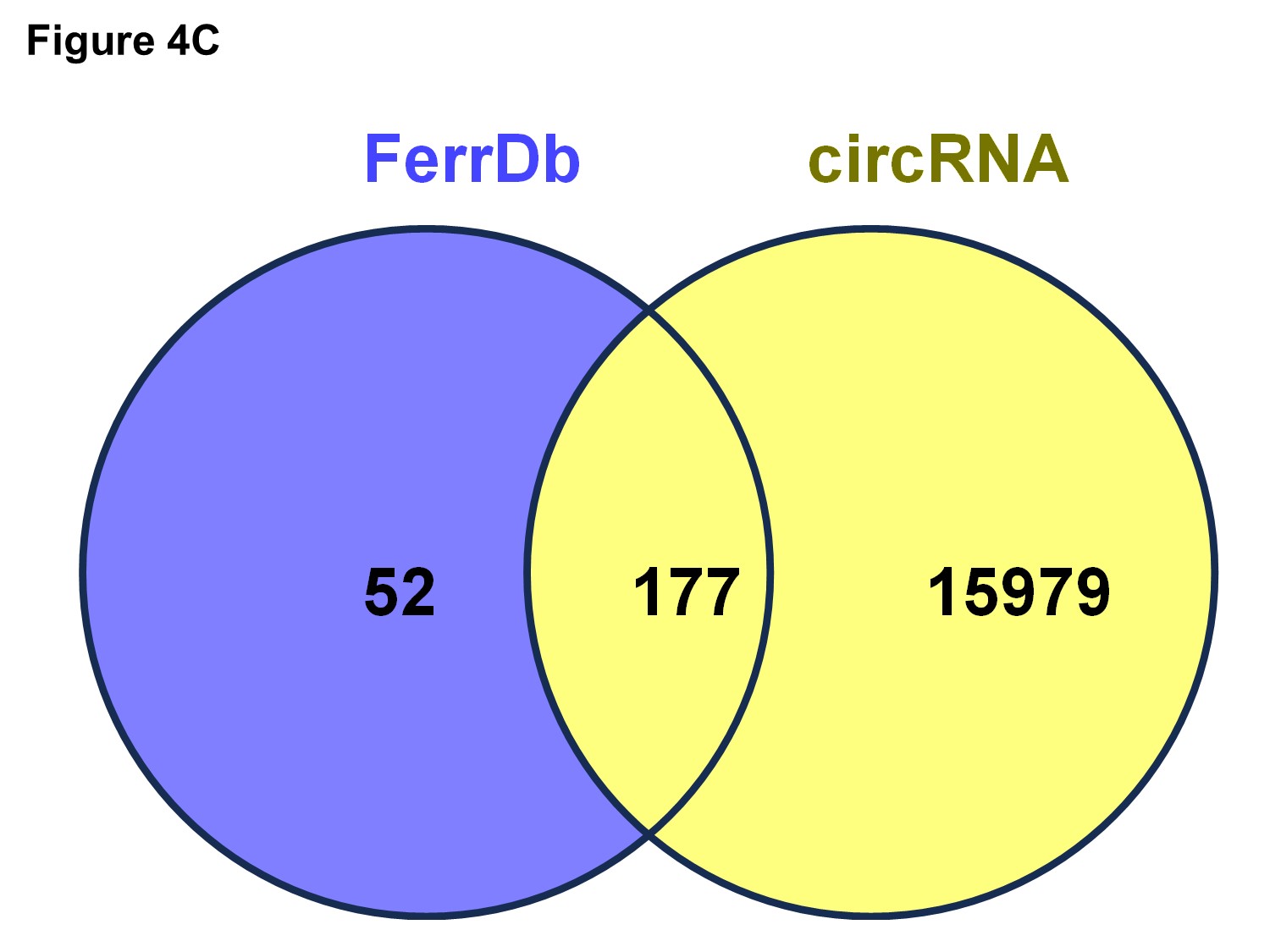

Supplement: Figure 4C.JPG [file IRNF_A_2490200_SM8368.jpg]

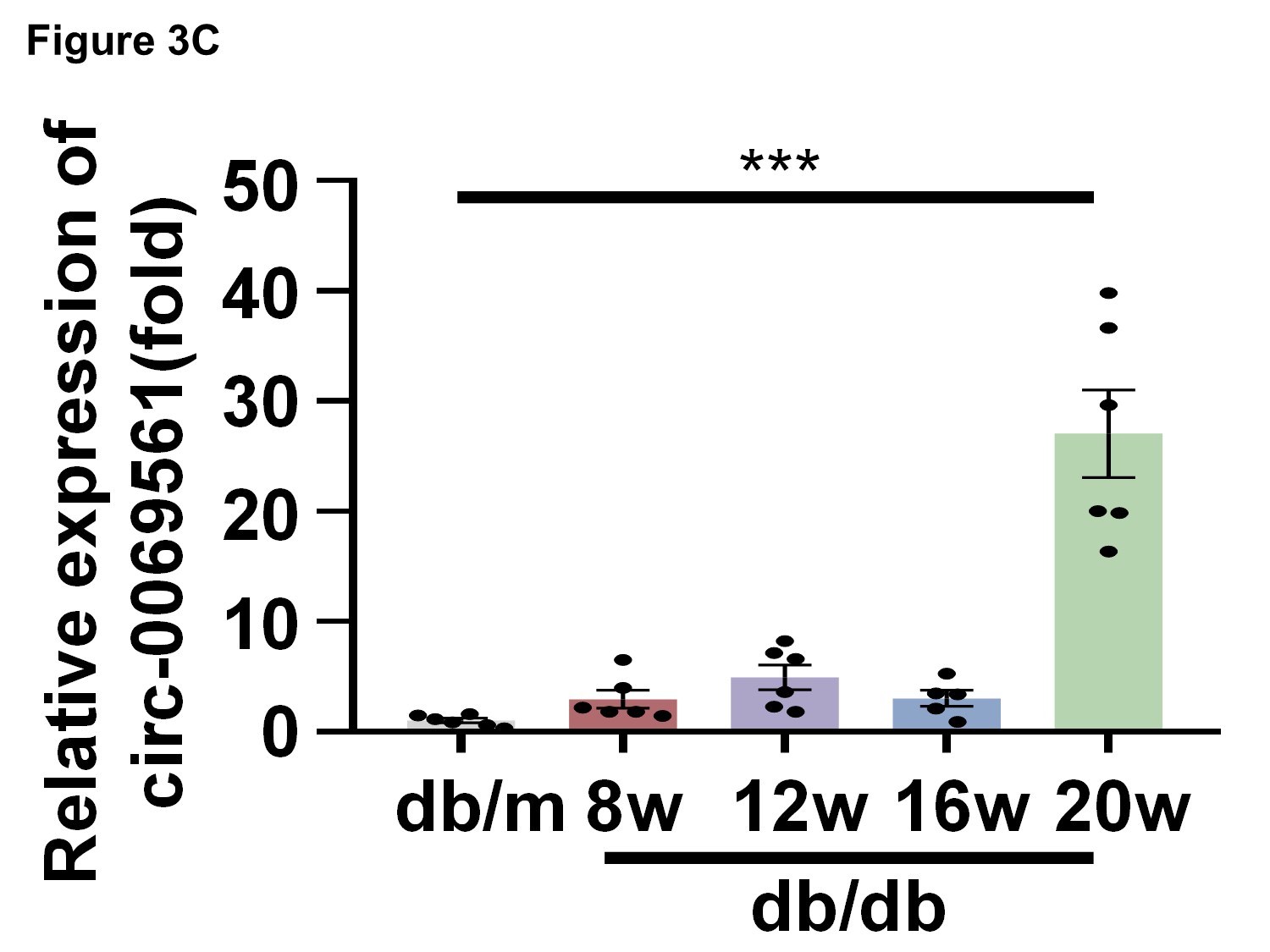

Supplement: Figure 3C.JPG [file IRNF_A_2490200_SM8367.jpg]

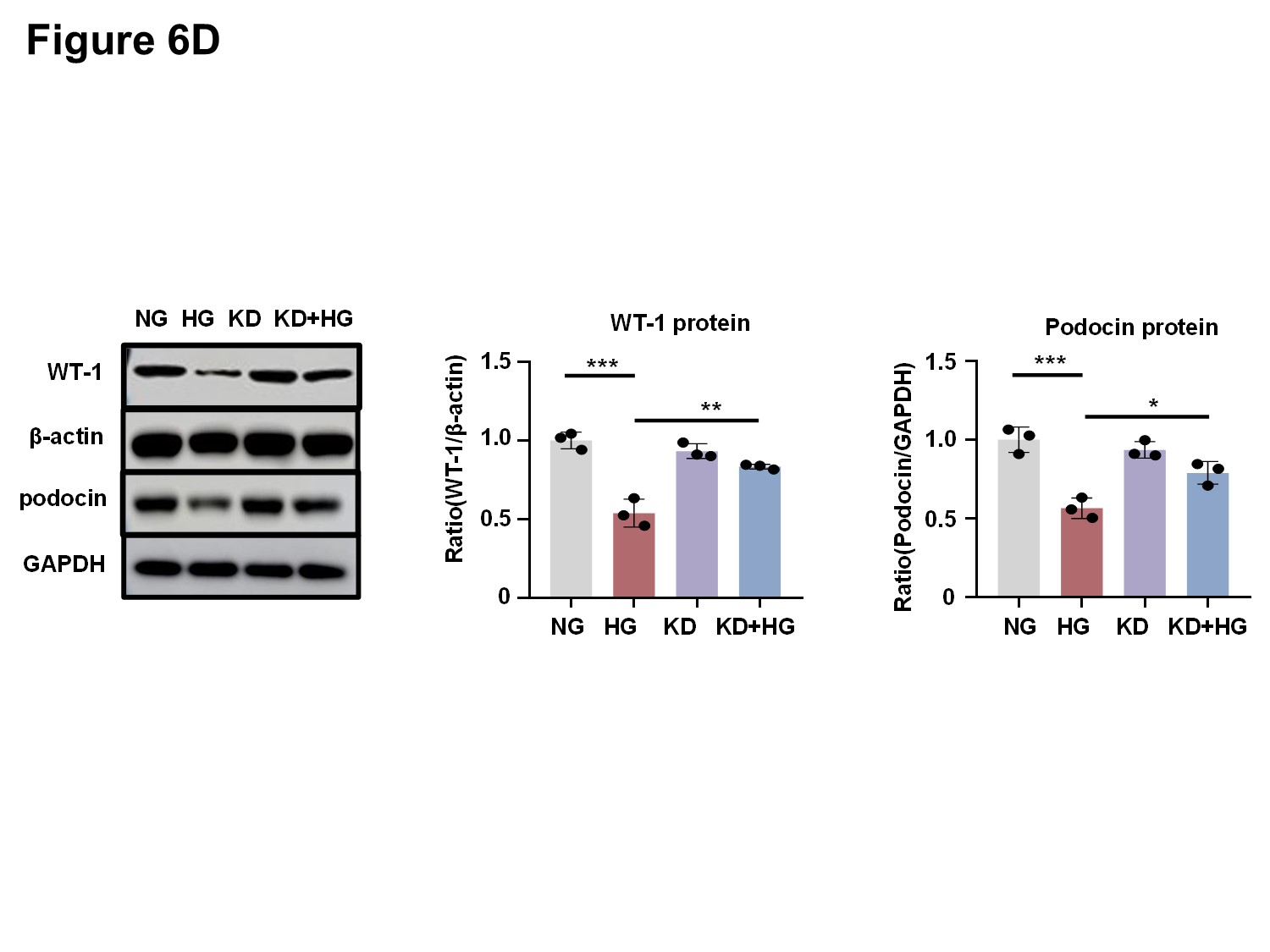

Supplement: Figure 6D.JPG [file IRNF_A_2490200_SM8366.jpg]

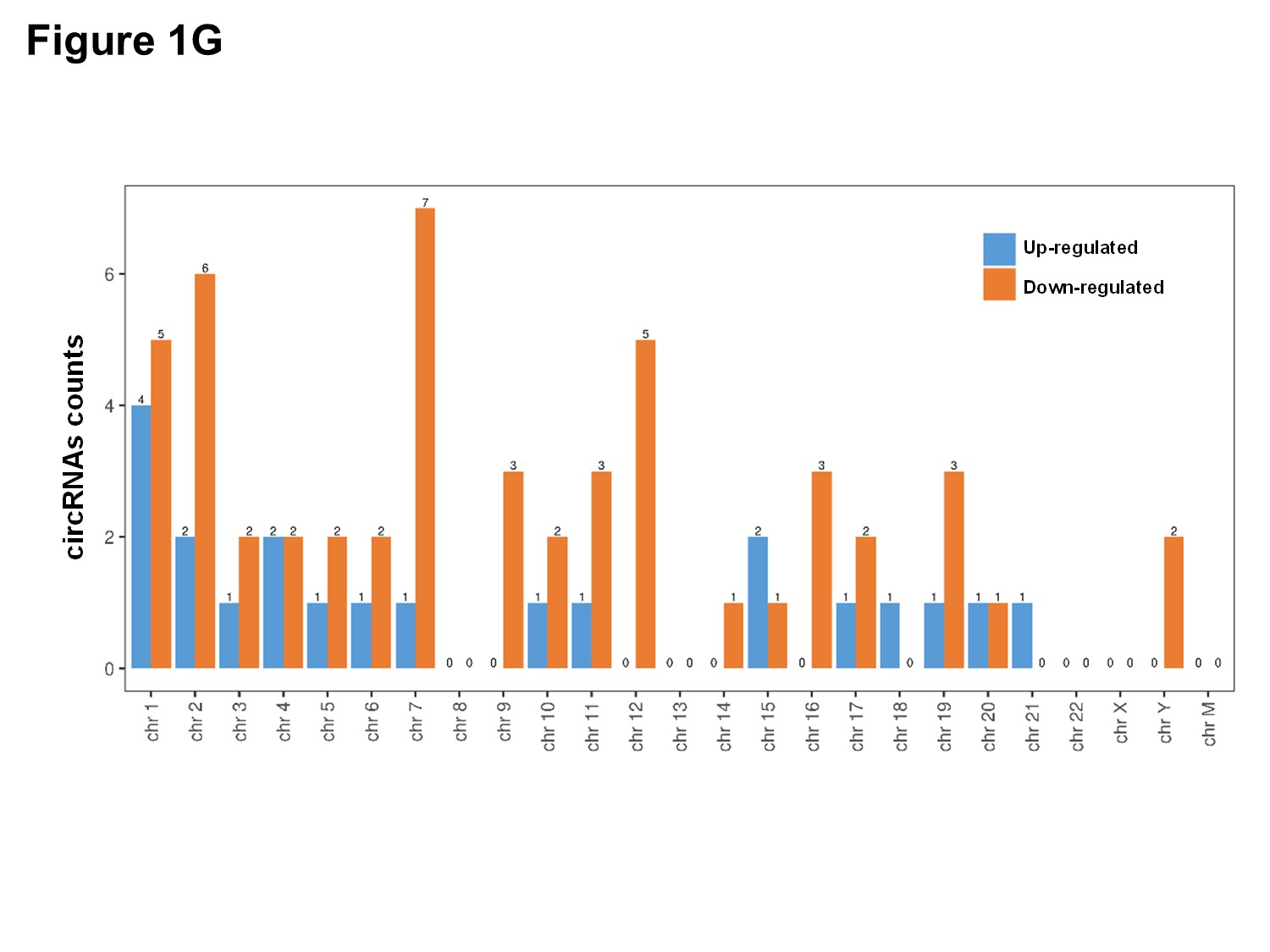

Supplement: Figure 1G.JPG [file IRNF_A_2490200_SM8365.jpg]

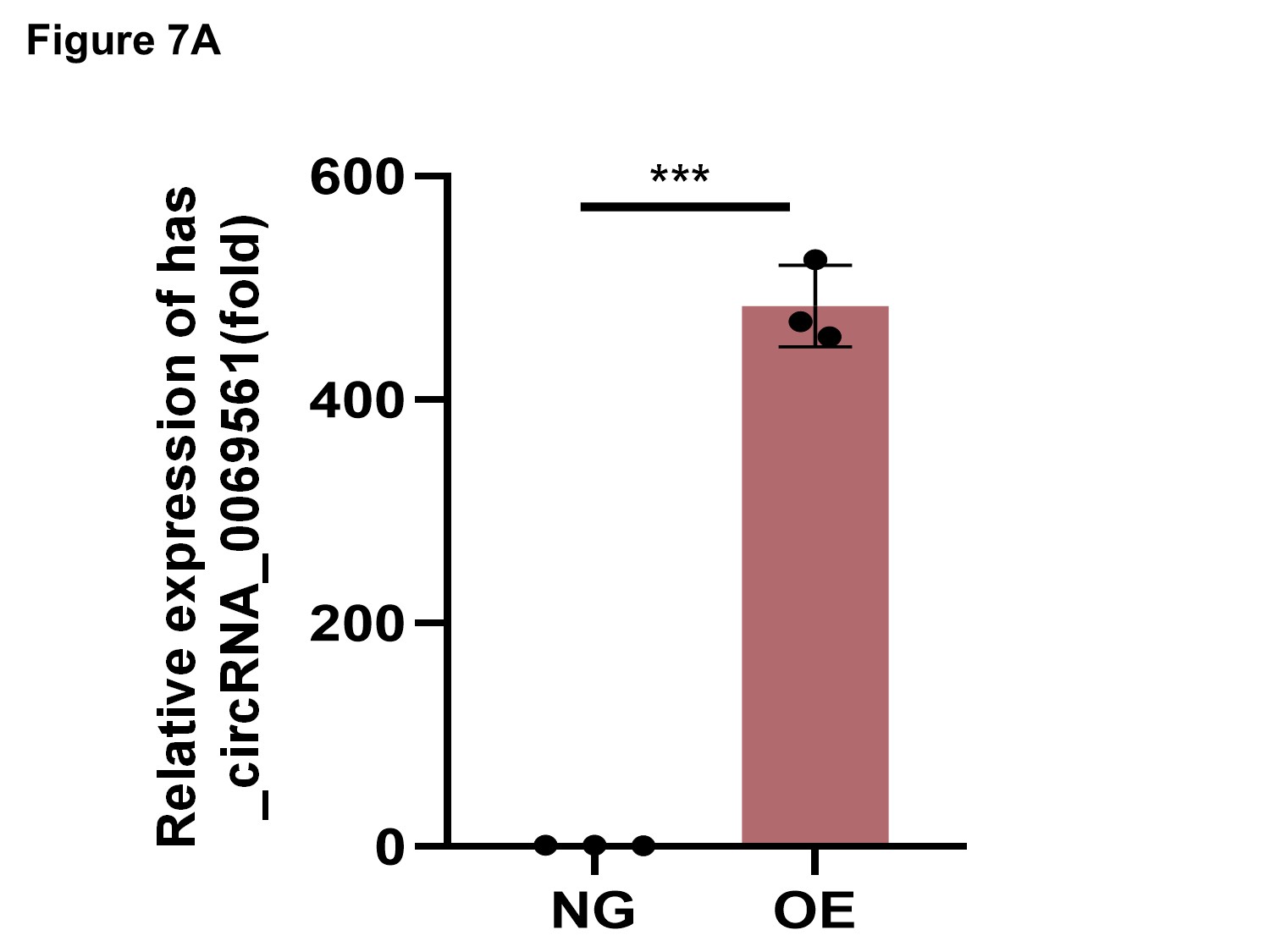

Supplement: Figure 7A.JPG [file IRNF_A_2490200_SM8364.jpg]

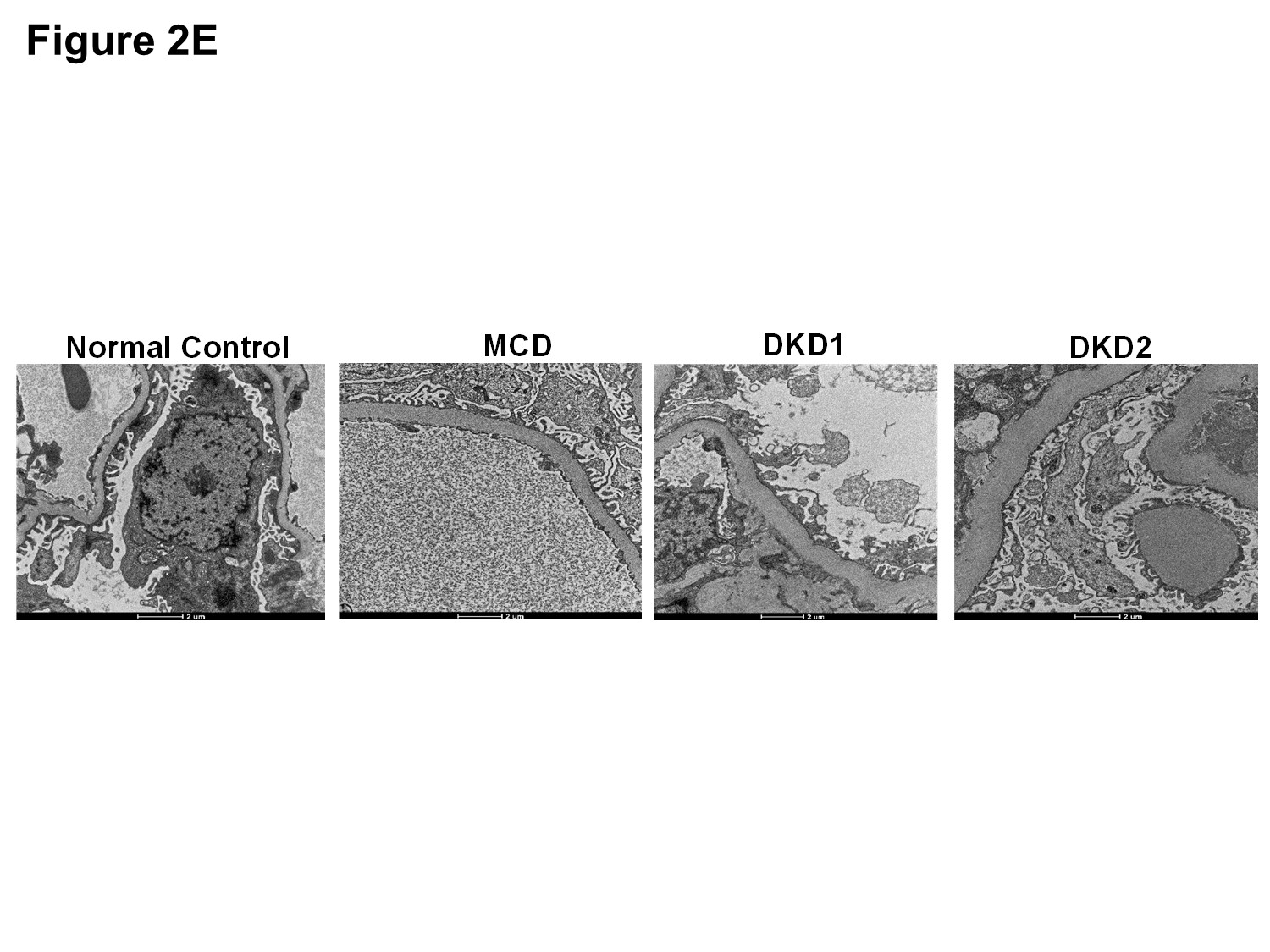

Supplement: Figure 2E.JPG [file IRNF_A_2490200_SM8363.jpg]

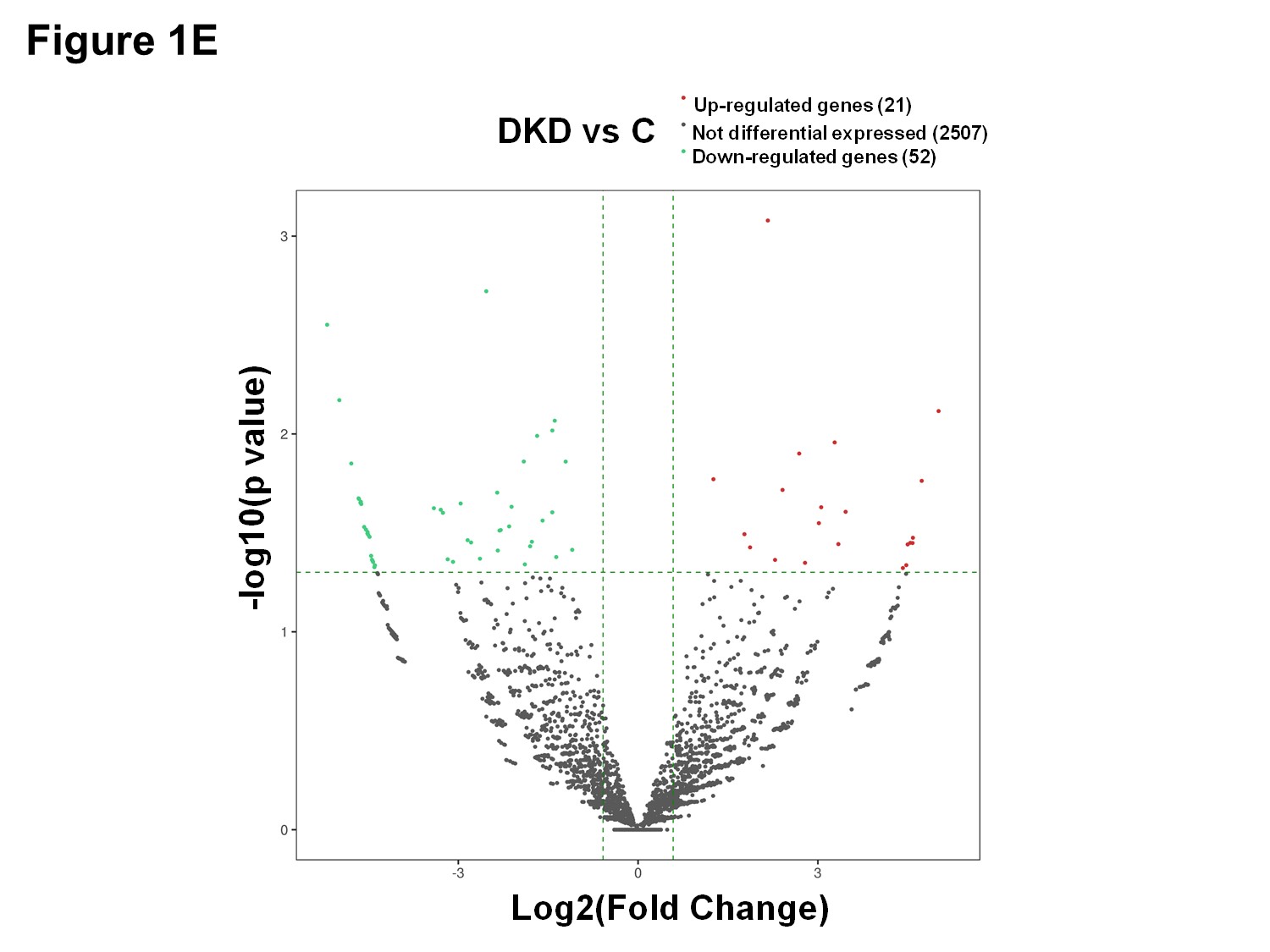

Supplement: Figure 1E.JPG [file IRNF_A_2490200_SM8362.jpg]

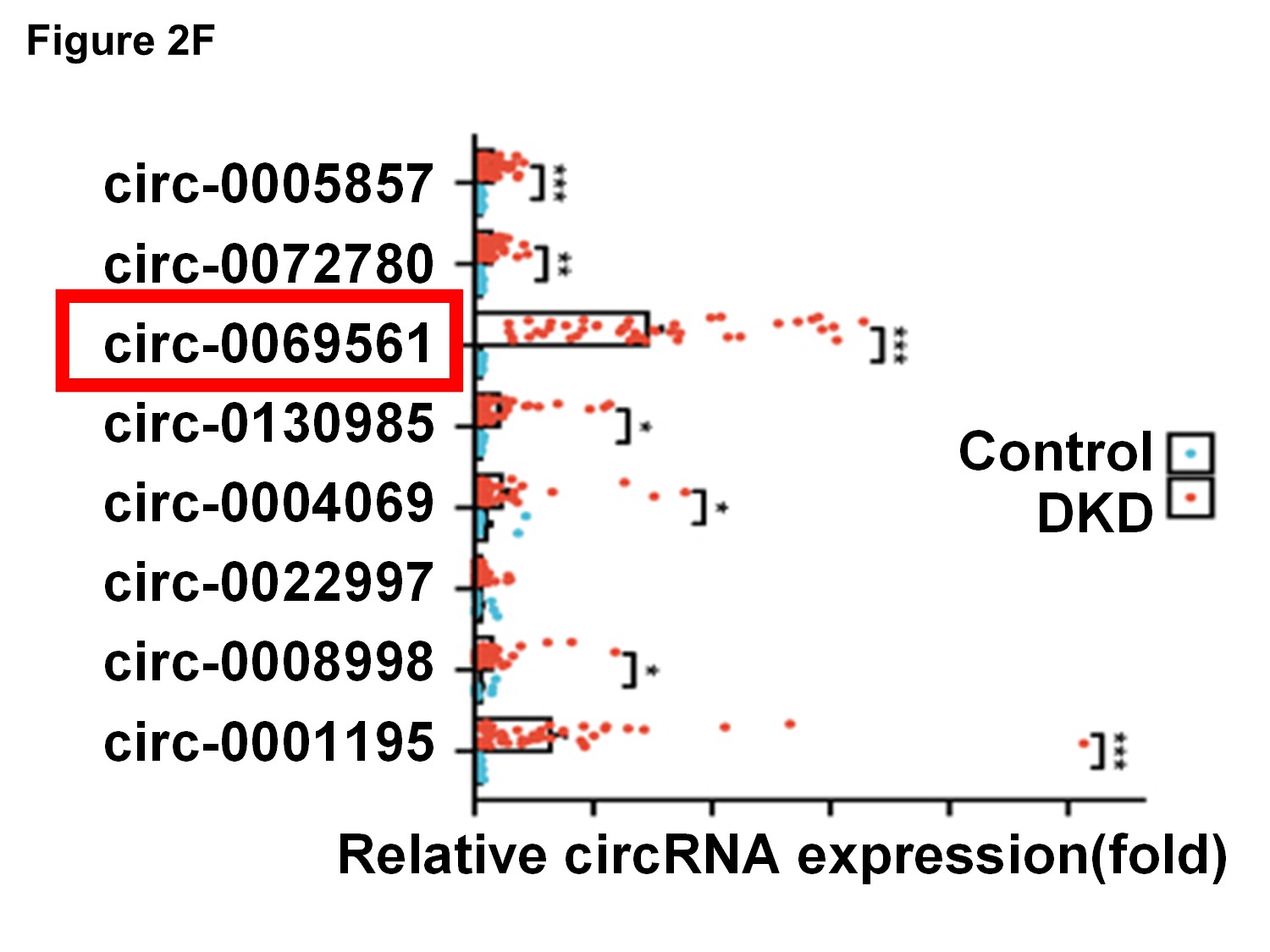

Supplement: Figure 2F.JPG [file IRNF_A_2490200_SM8361.jpg]

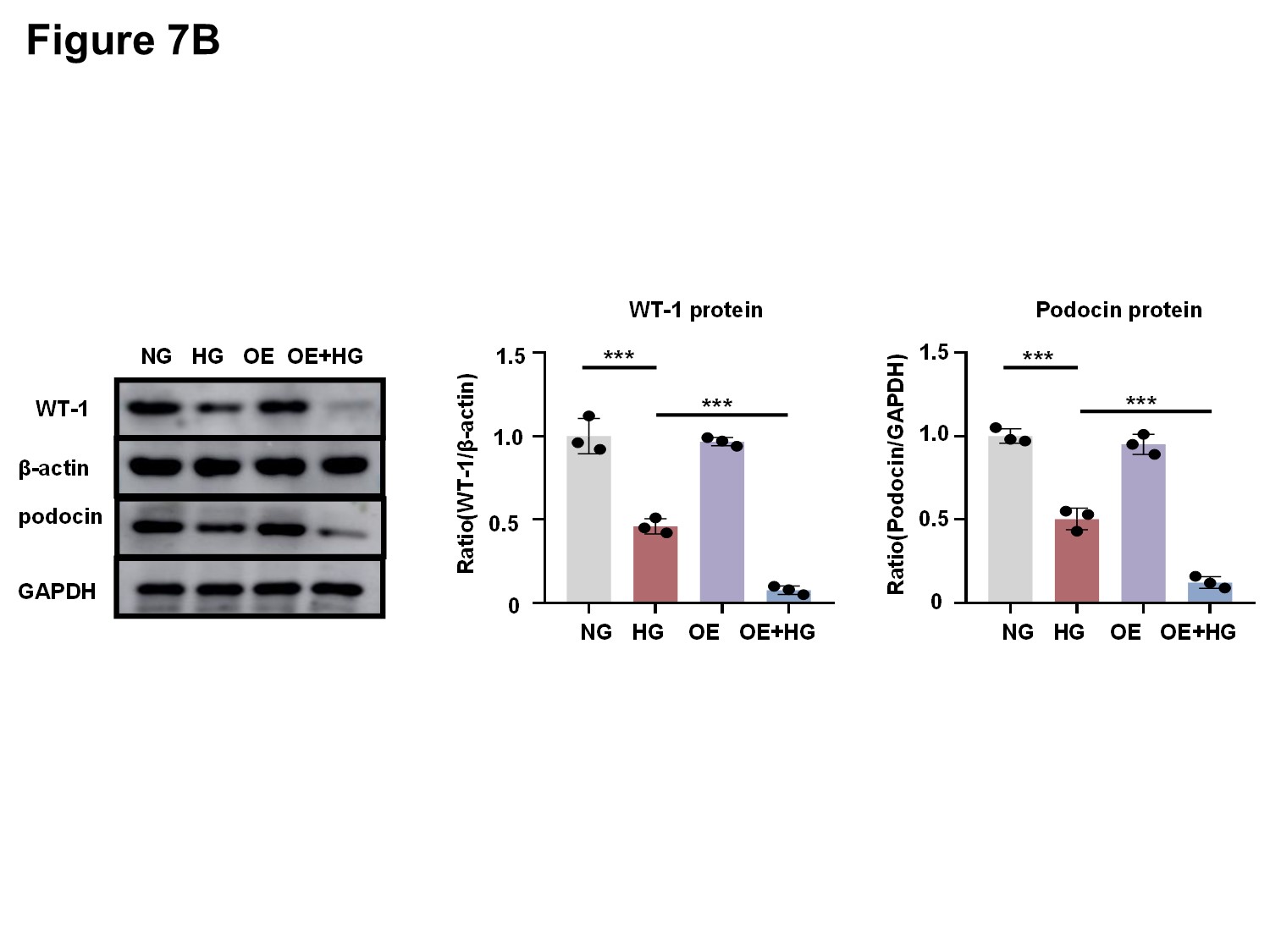

Supplement: Figure 7B.JPG [file IRNF_A_2490200_SM8360.jpg]

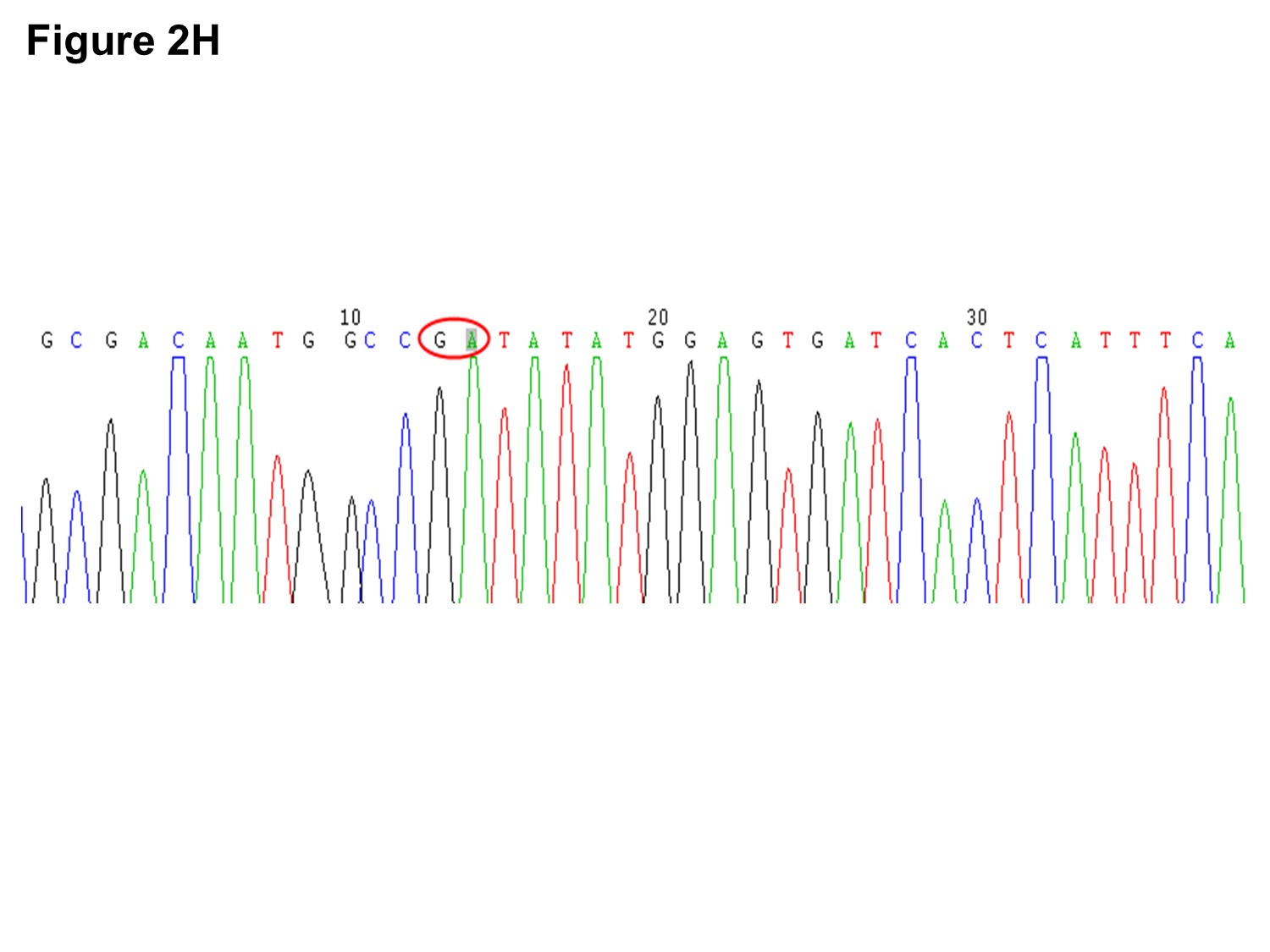

Supplement: Figure 2H.JPG [file IRNF_A_2490200_SM8359.jpg]

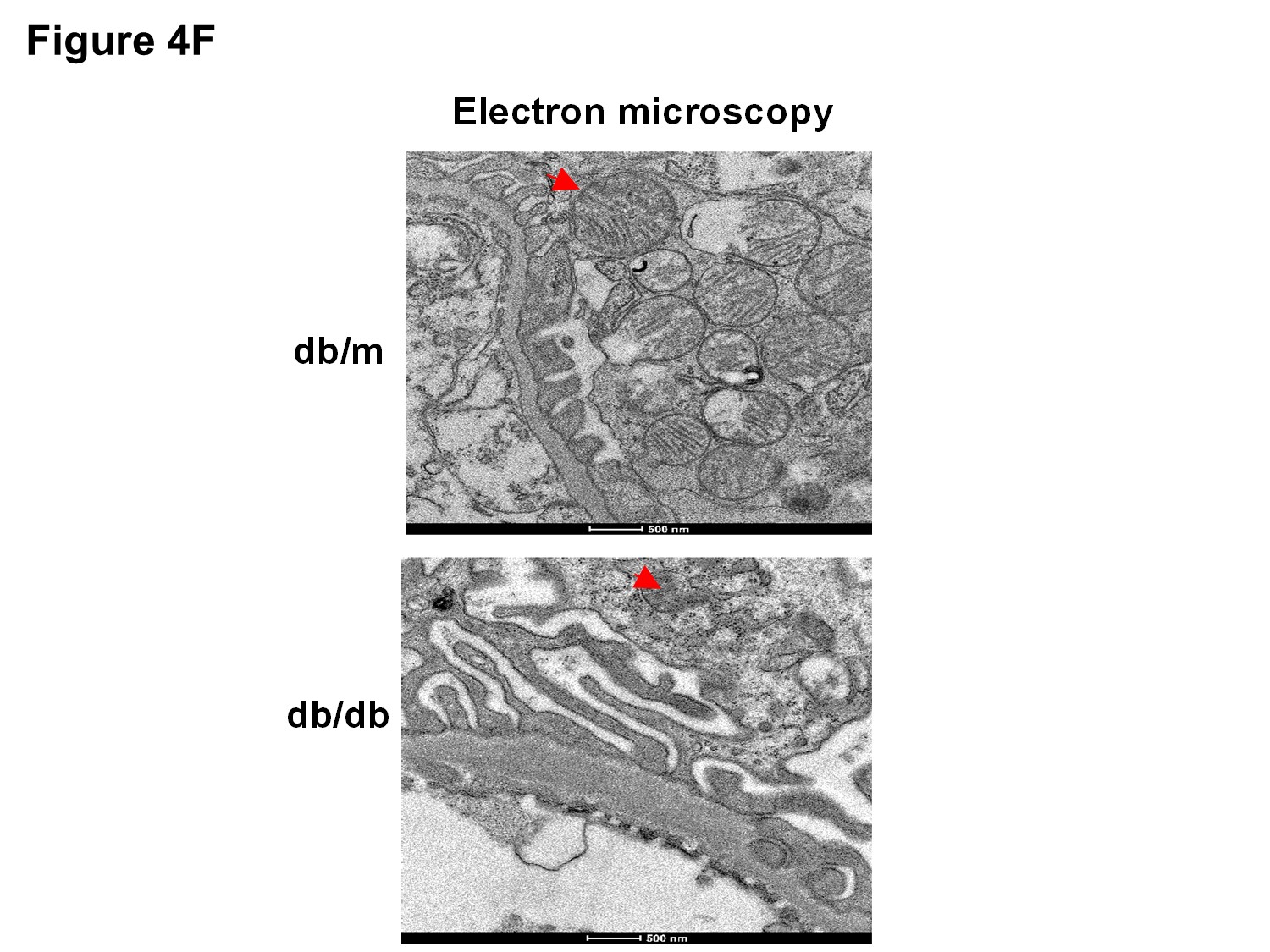

Supplement: Figure 4F.JPG [file IRNF_A_2490200_SM8358.jpg]

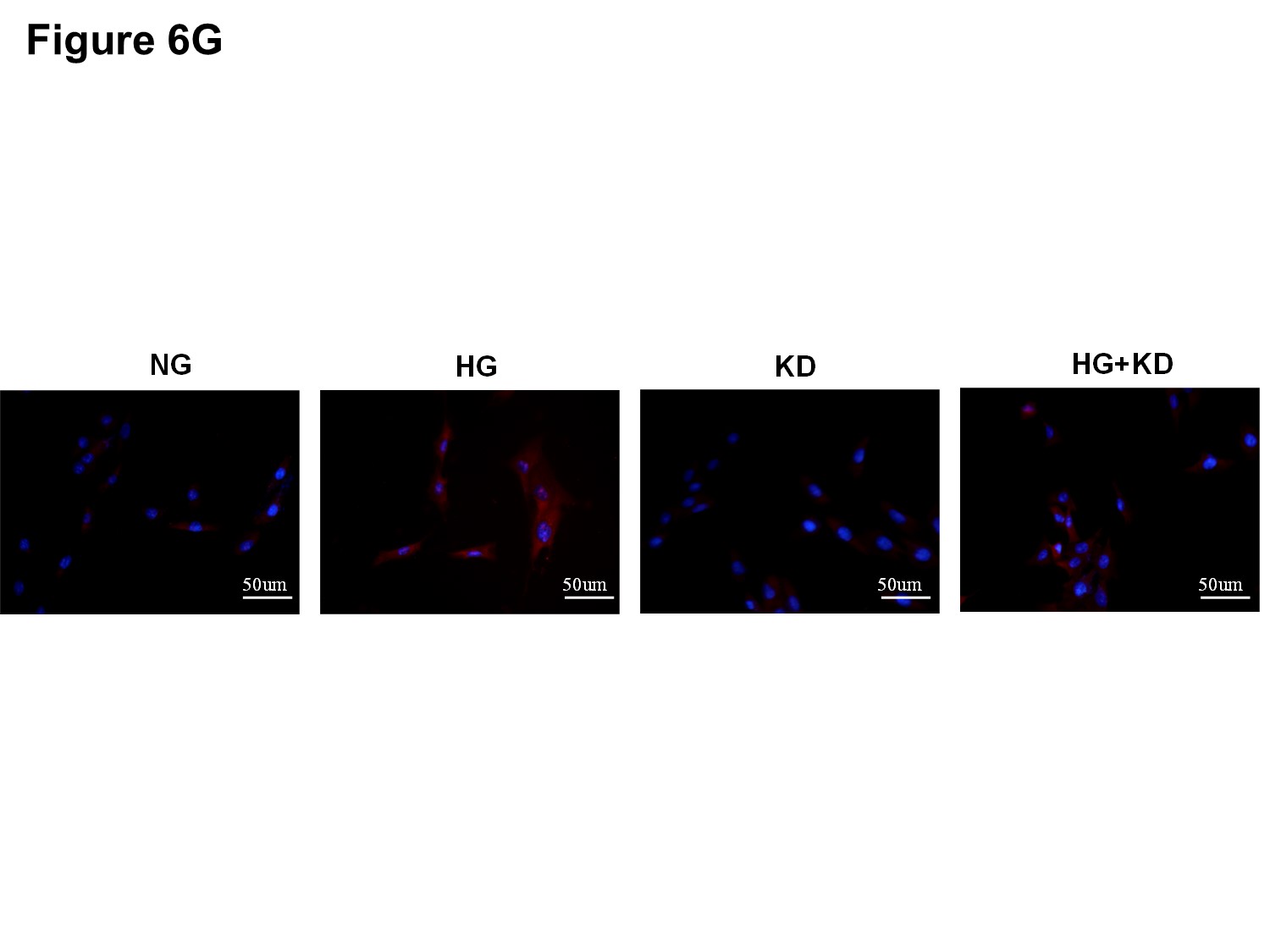

Supplement: Figure 6G.JPG [file IRNF_A_2490200_SM8357.jpg]

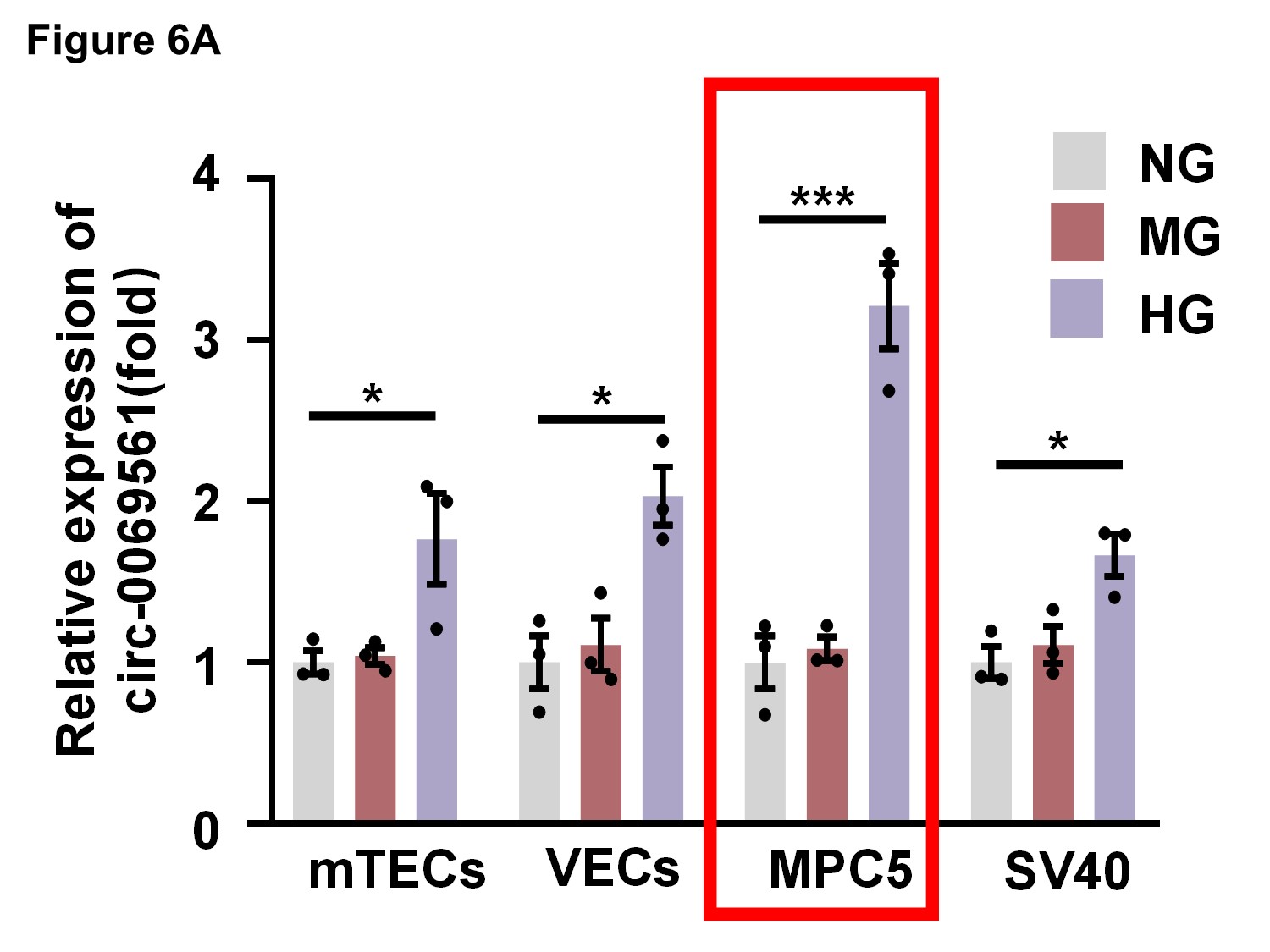

Supplement: Figure 6A.JPG [file IRNF_A_2490200_SM8356.jpg]

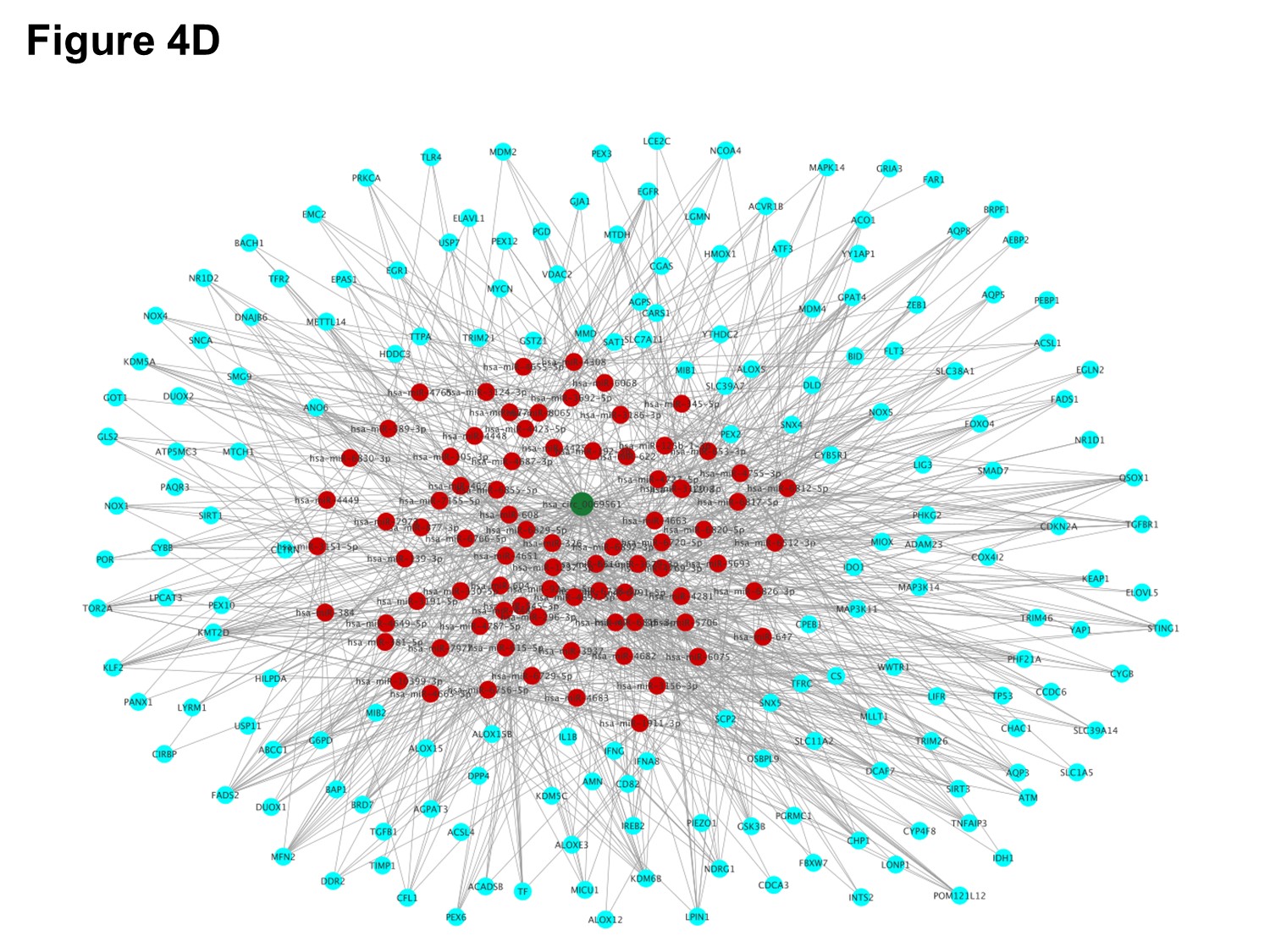

Supplement: Figure 4D.JPG [file IRNF_A_2490200_SM8355.jpg]

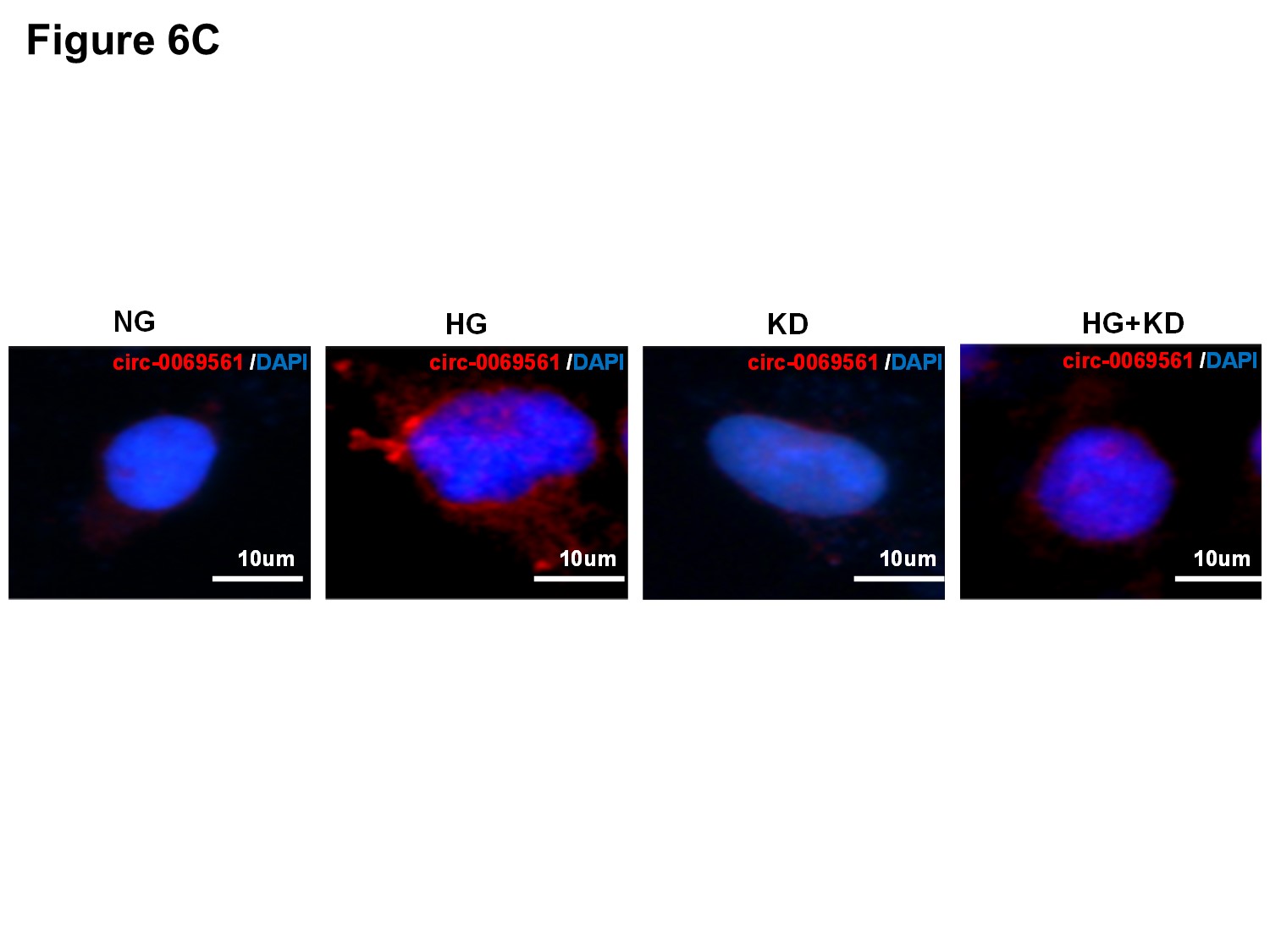

Supplement: Figure 6C.JPG [file IRNF_A_2490200_SM8354.jpg]

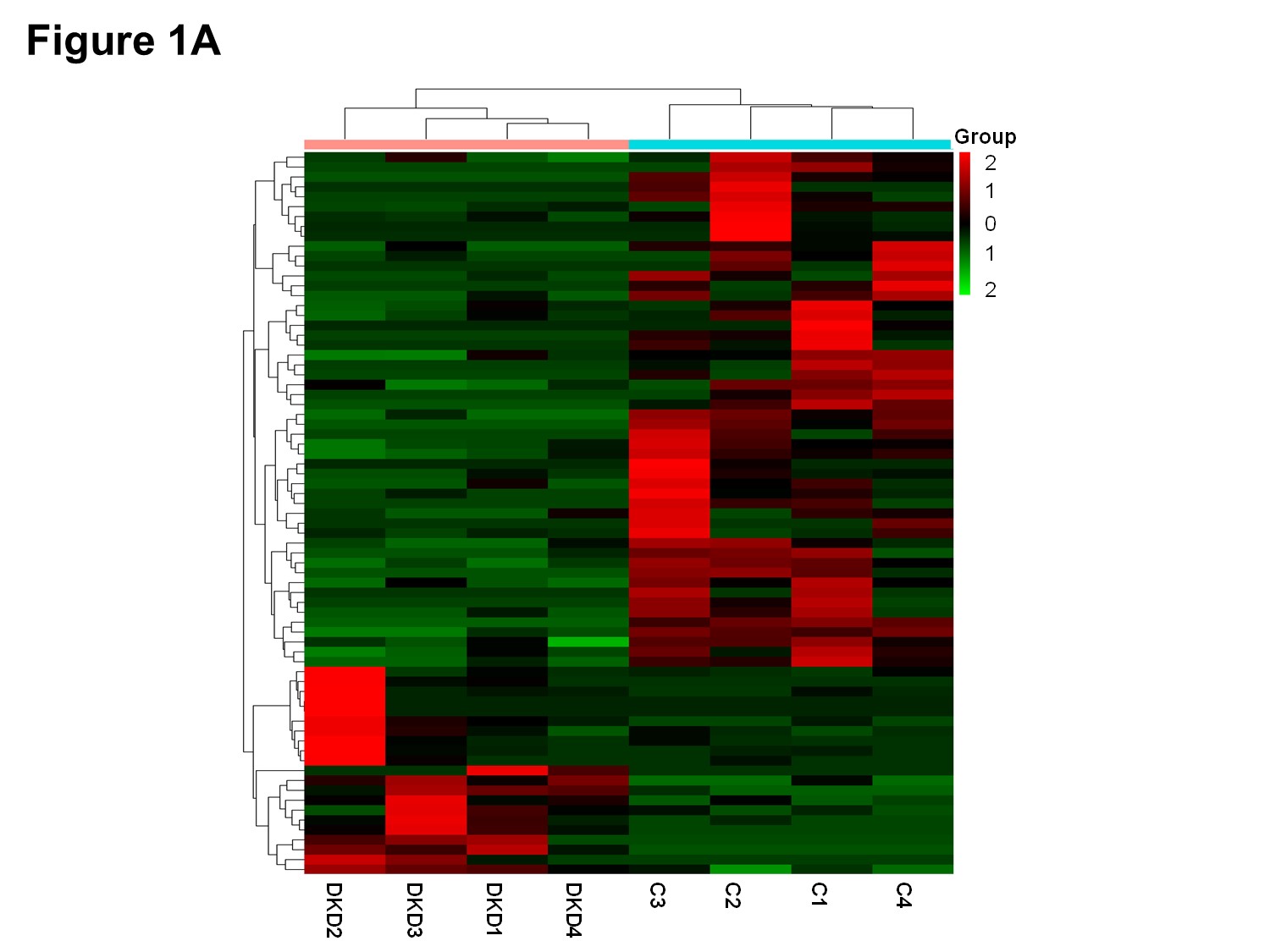

Supplement: Figure 1A.JPG [file IRNF_A_2490200_SM8353.jpg]

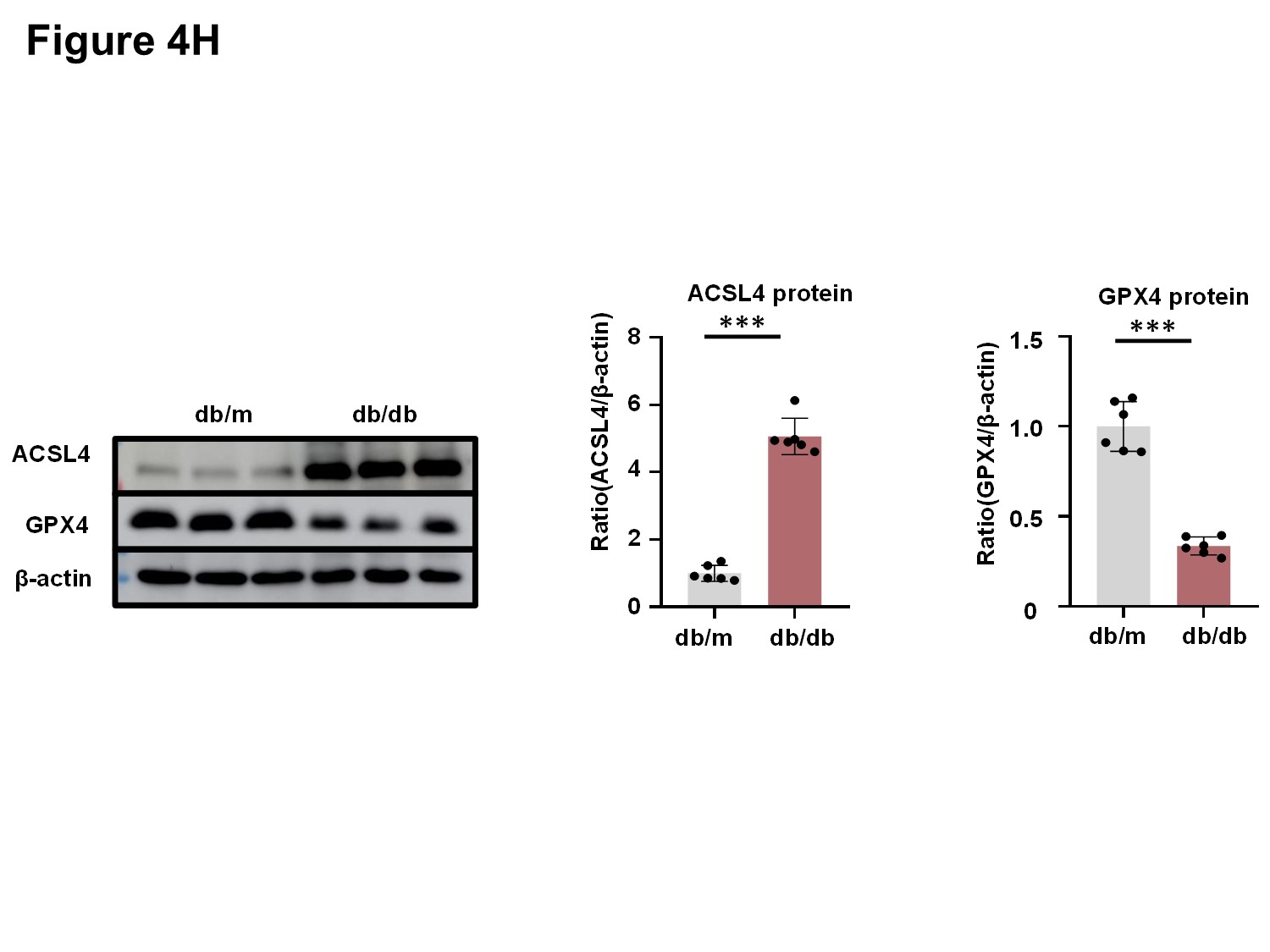

Supplement: Figure 4H.JPG [file IRNF_A_2490200_SM8352.jpg]

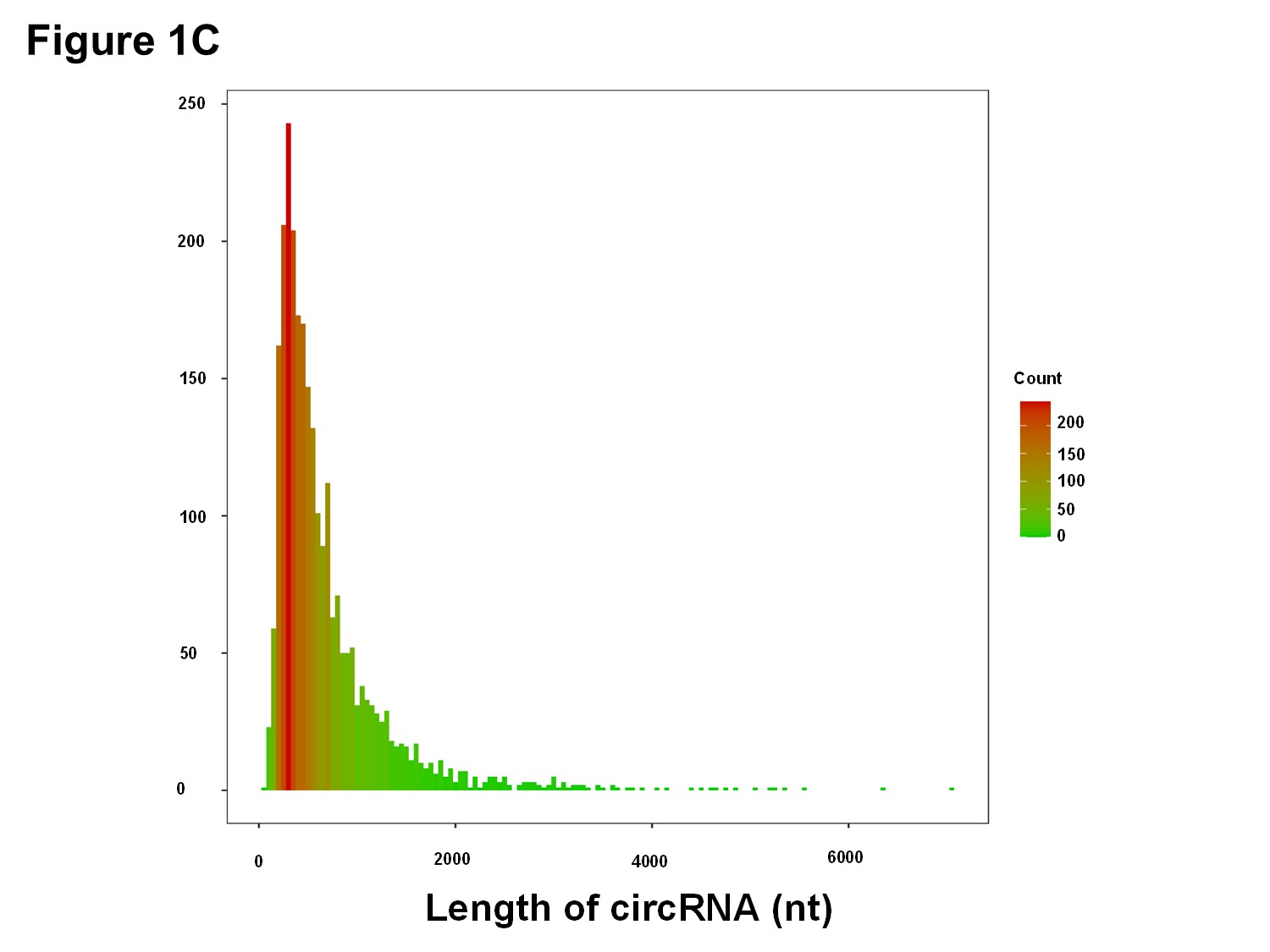

Supplement: Figure 1C.JPG [file IRNF_A_2490200_SM8351.jpg]

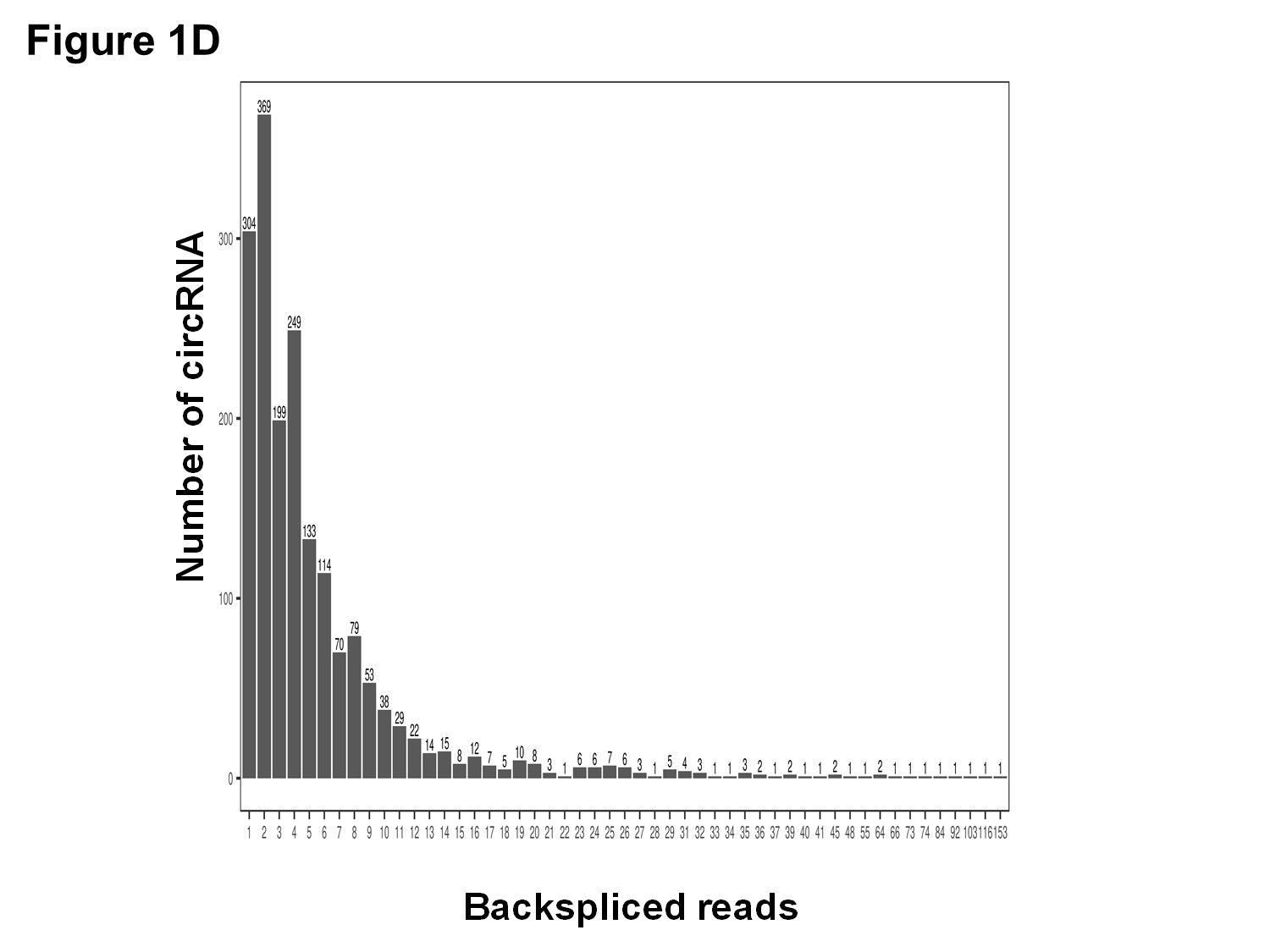

Supplement: Figure 1D.JPG [file IRNF_A_2490200_SM8350.jpg]

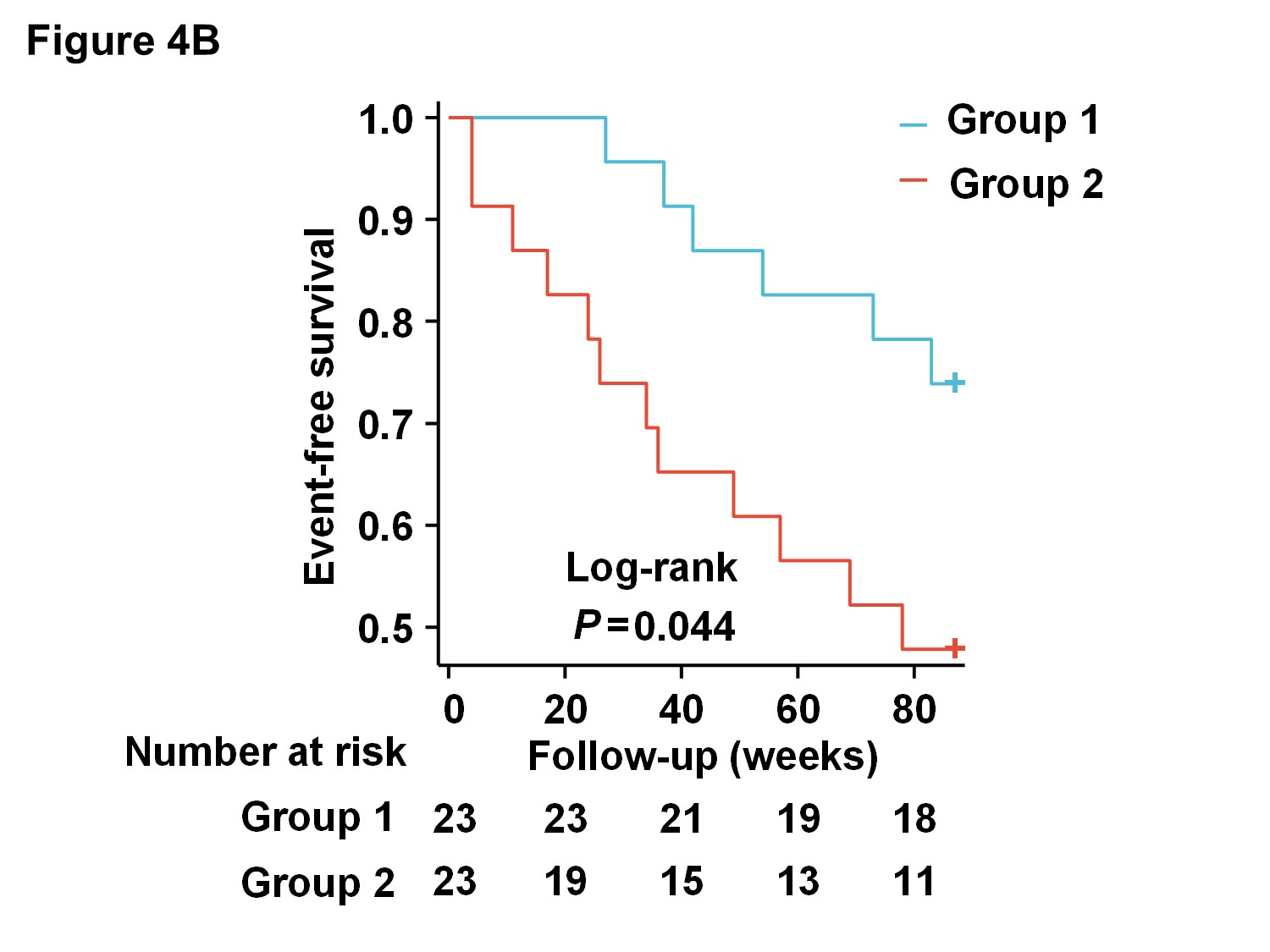

Supplement: Figure 4B.JPG [file IRNF_A_2490200_SM8349.jpg]

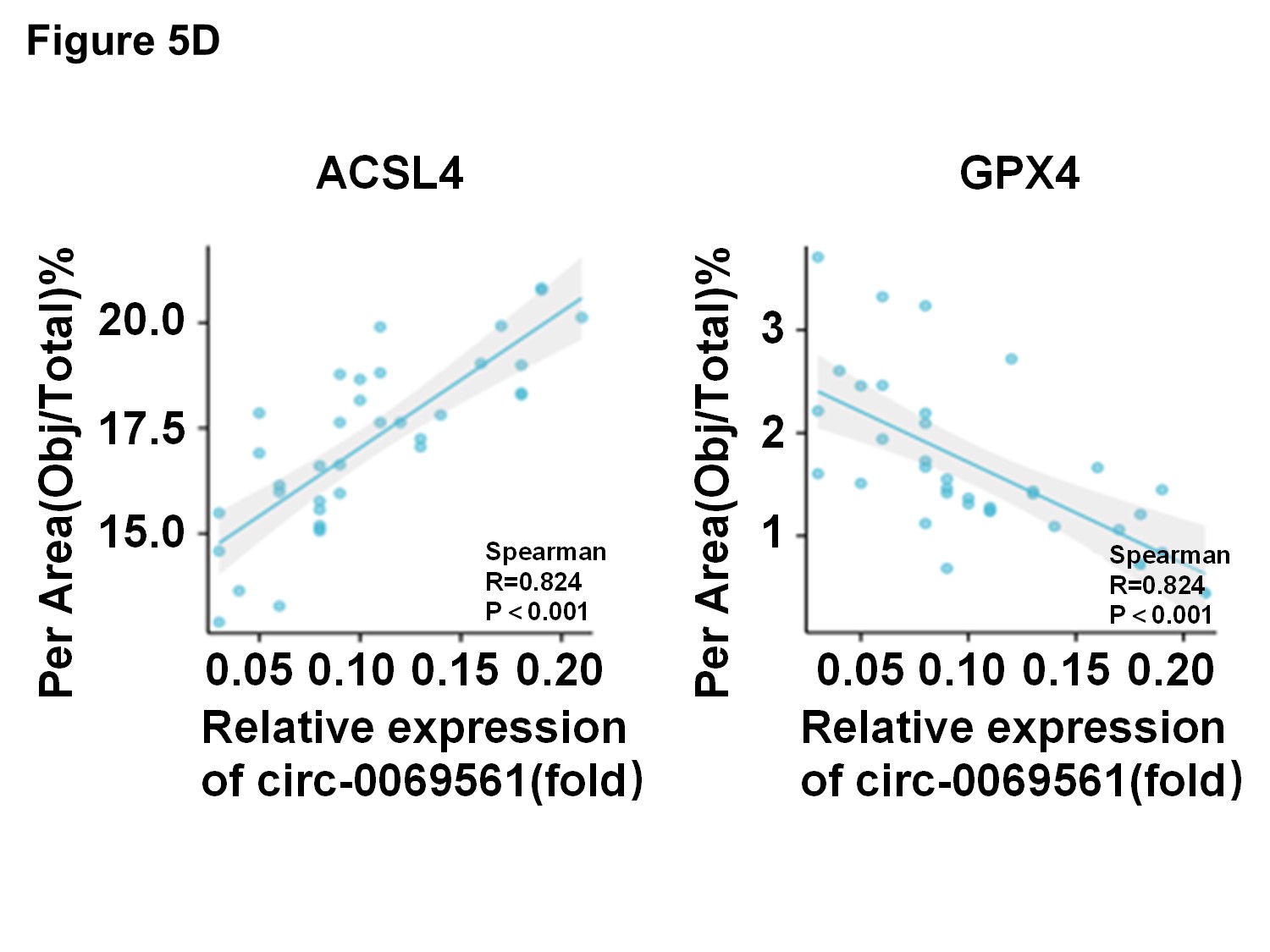

Supplement: Figure 5D.JPG [file IRNF_A_2490200_SM8348.jpg]

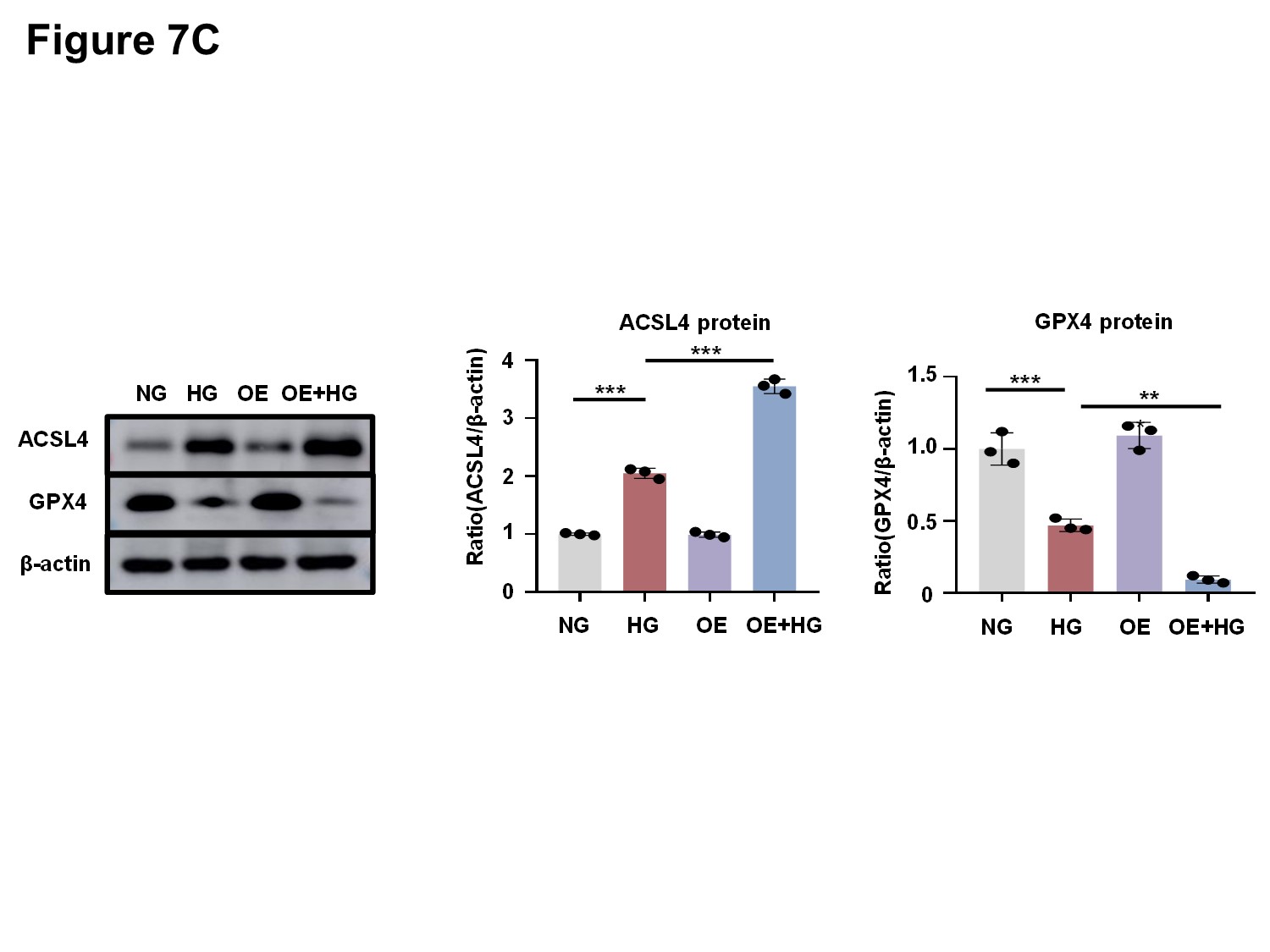

Supplement: Figure 7C.JPG [file IRNF_A_2490200_SM8347.jpg]

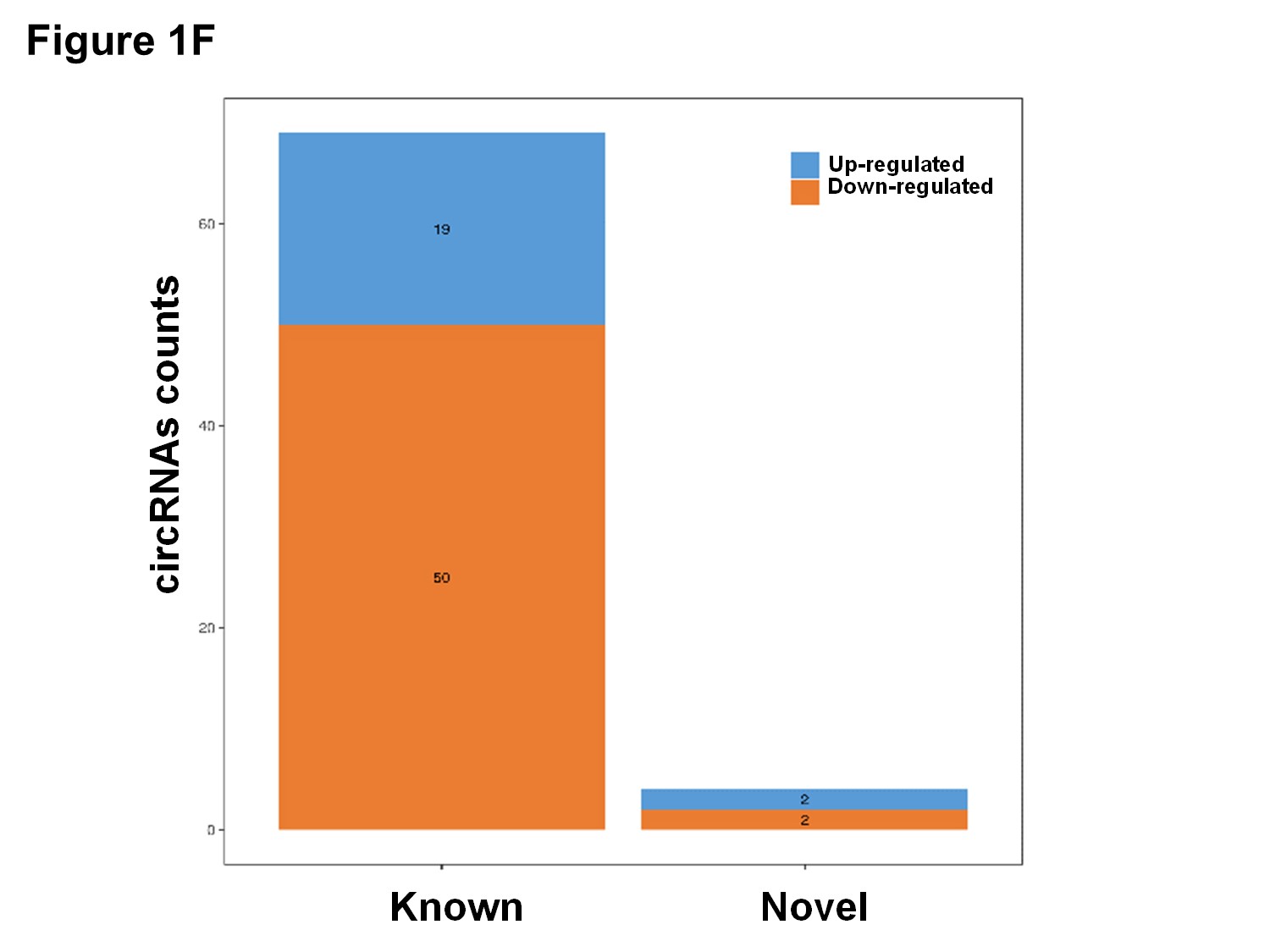

Supplement: Figure 1F.JPG [file IRNF_A_2490200_SM8346.jpg]

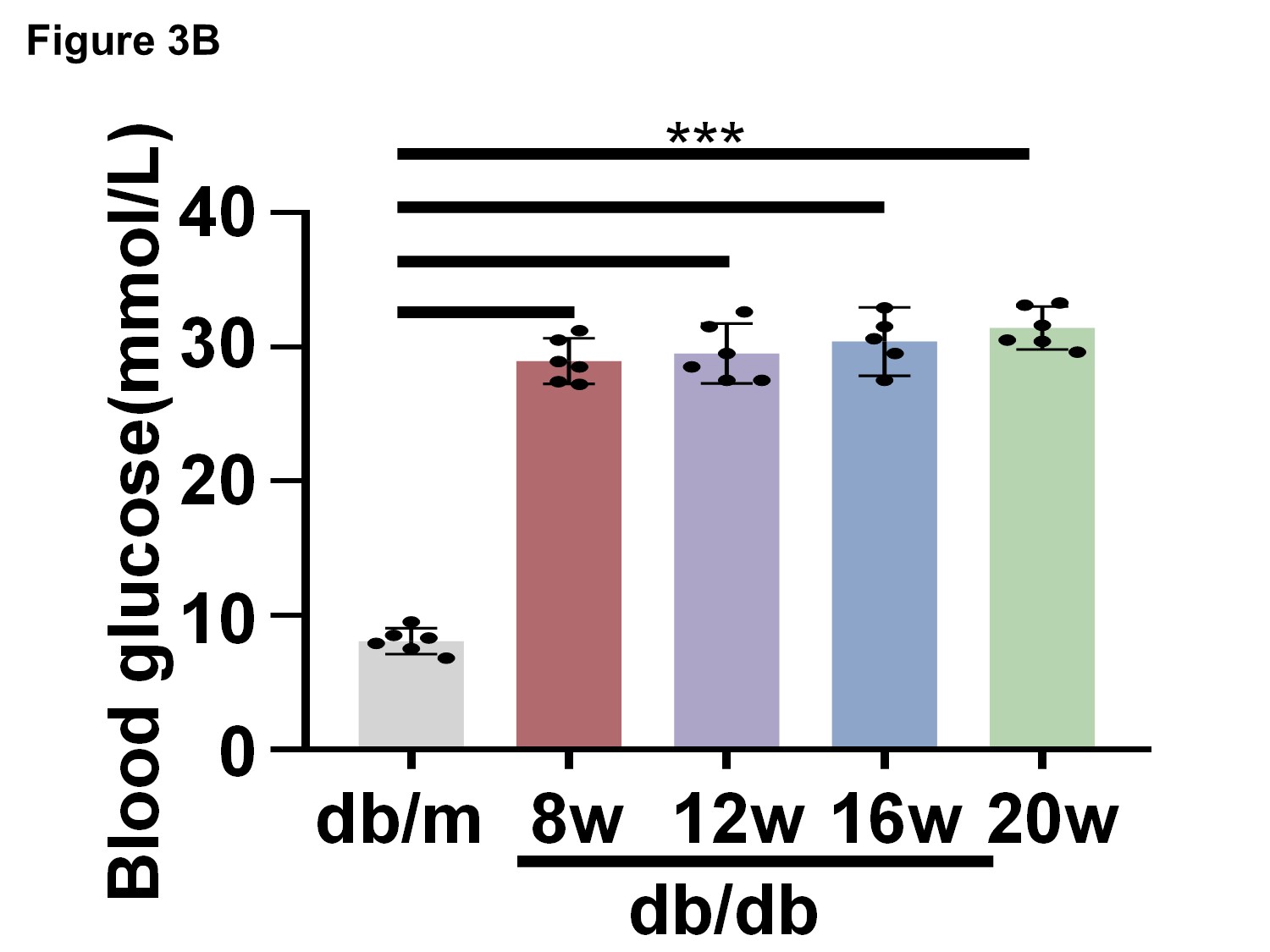

Supplement: Figure 3B.JPG [file IRNF_A_2490200_SM8345.jpg]

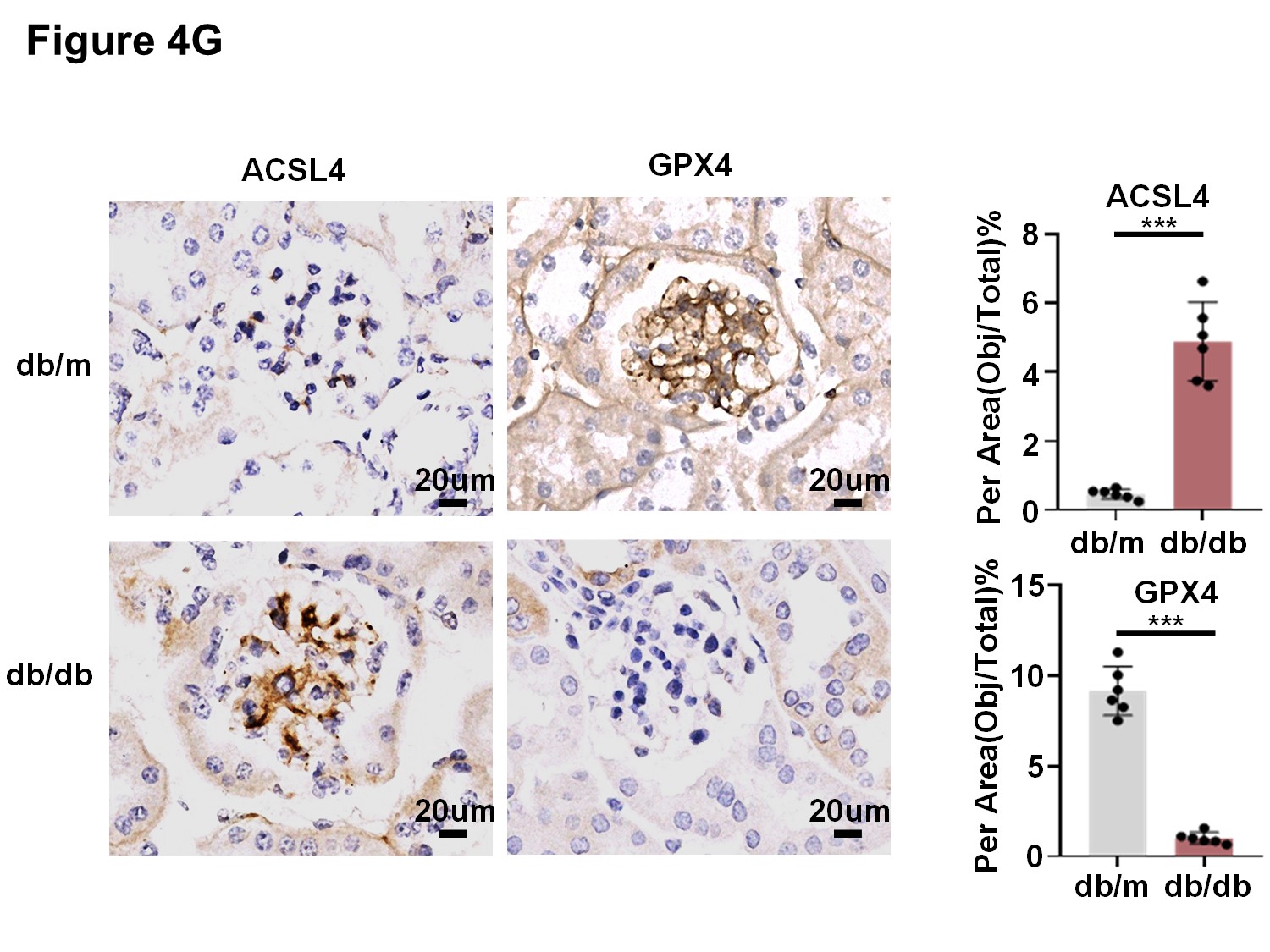

Supplement: Figure 4G.JPG [file IRNF_A_2490200_SM8344.jpg]

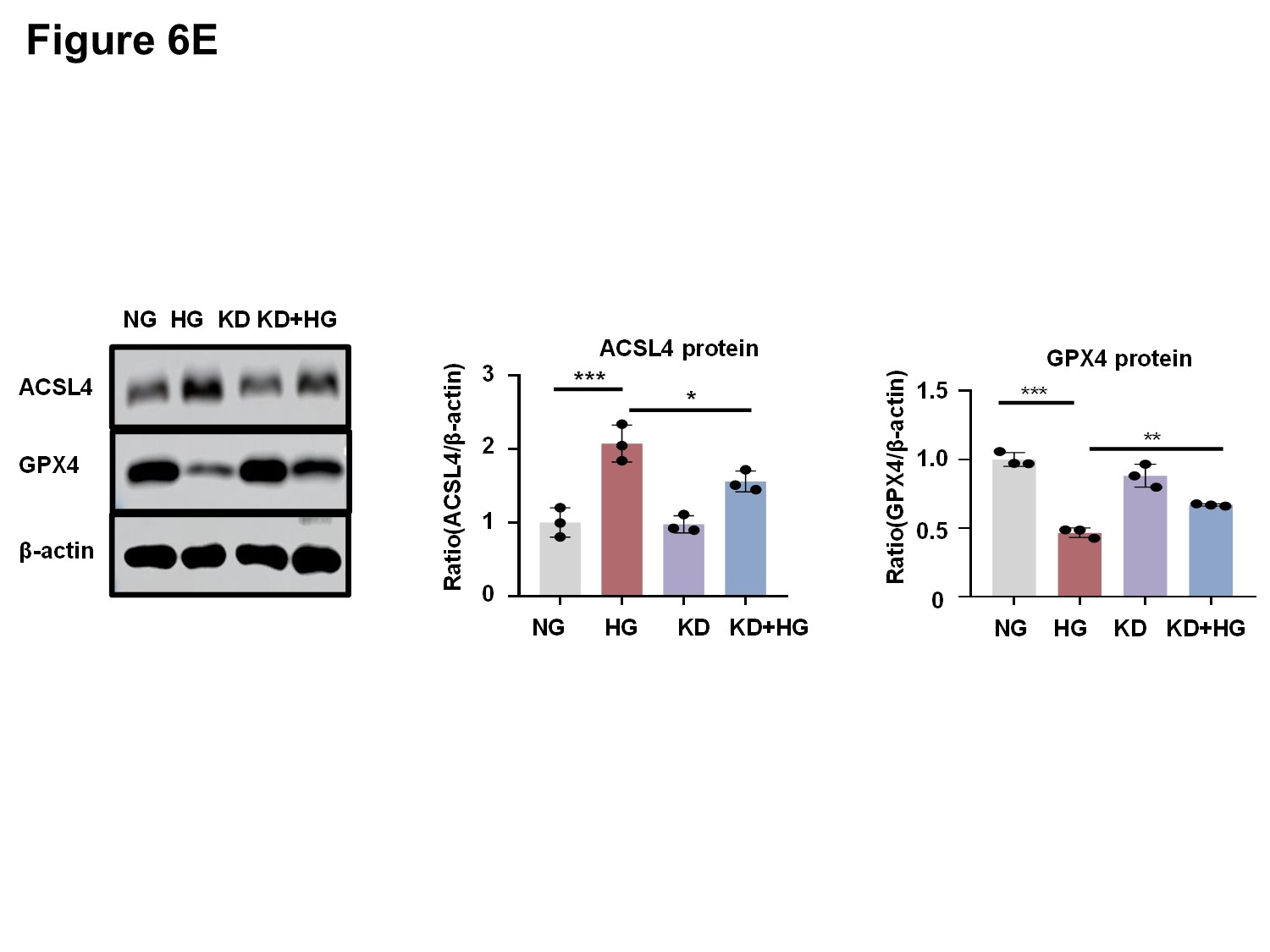

Supplement: Figure 6E.JPG [file IRNF_A_2490200_SM8343.jpg]

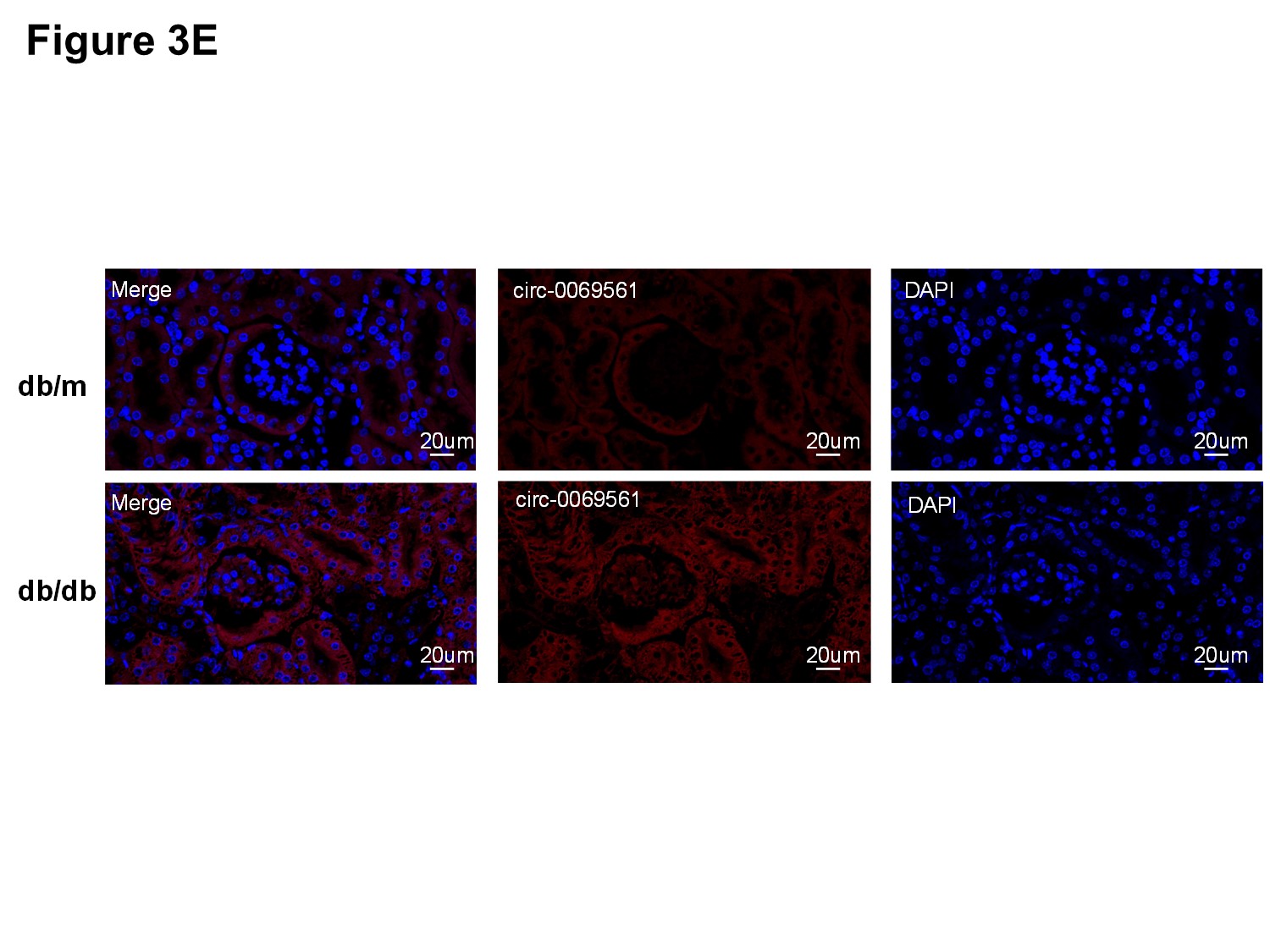

Supplement: Figure 3E.JPG [file IRNF_A_2490200_SM8342.jpg]

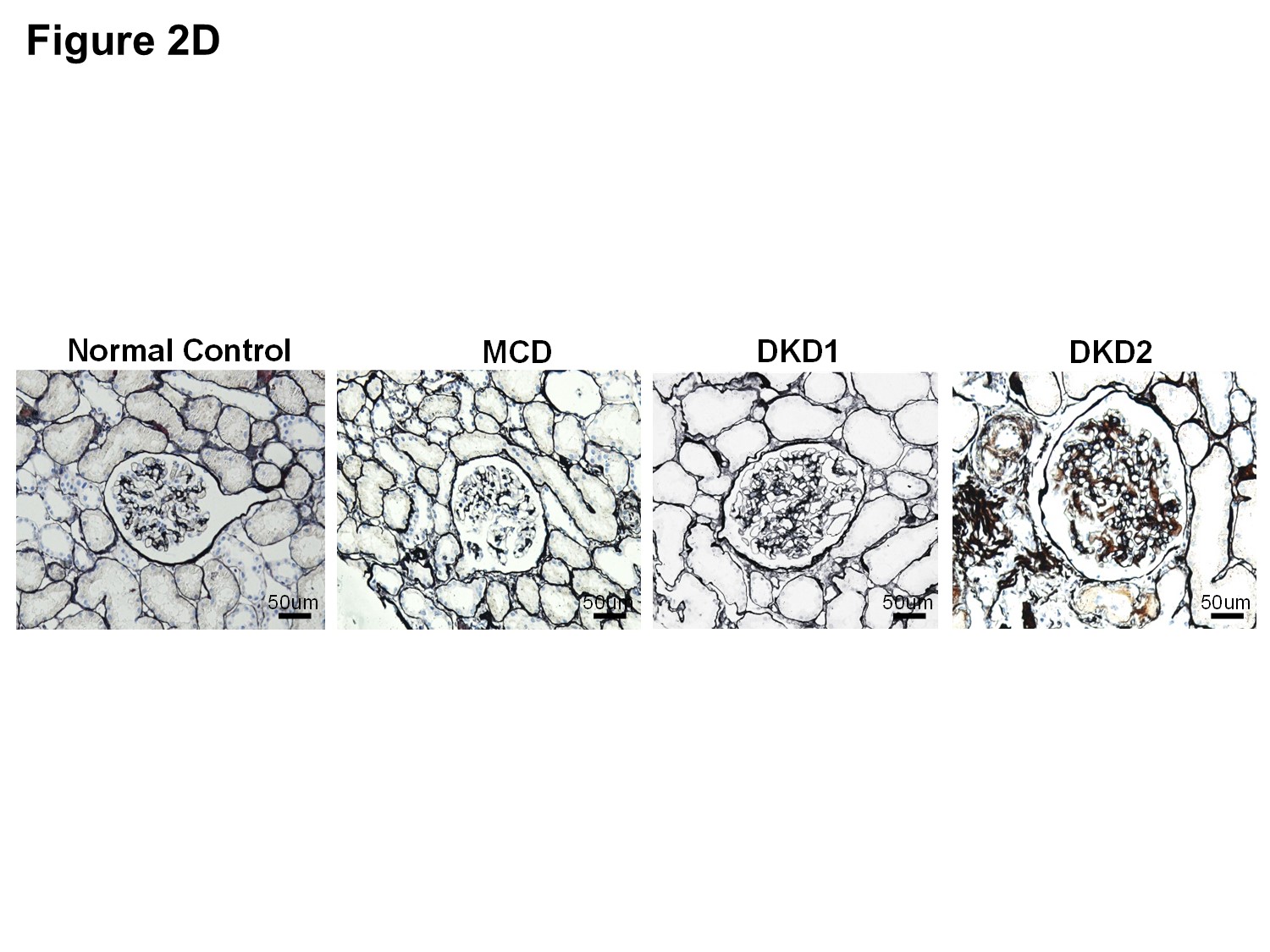

Supplement: Figure 2D.JPG [file IRNF_A_2490200_SM8341.jpg]

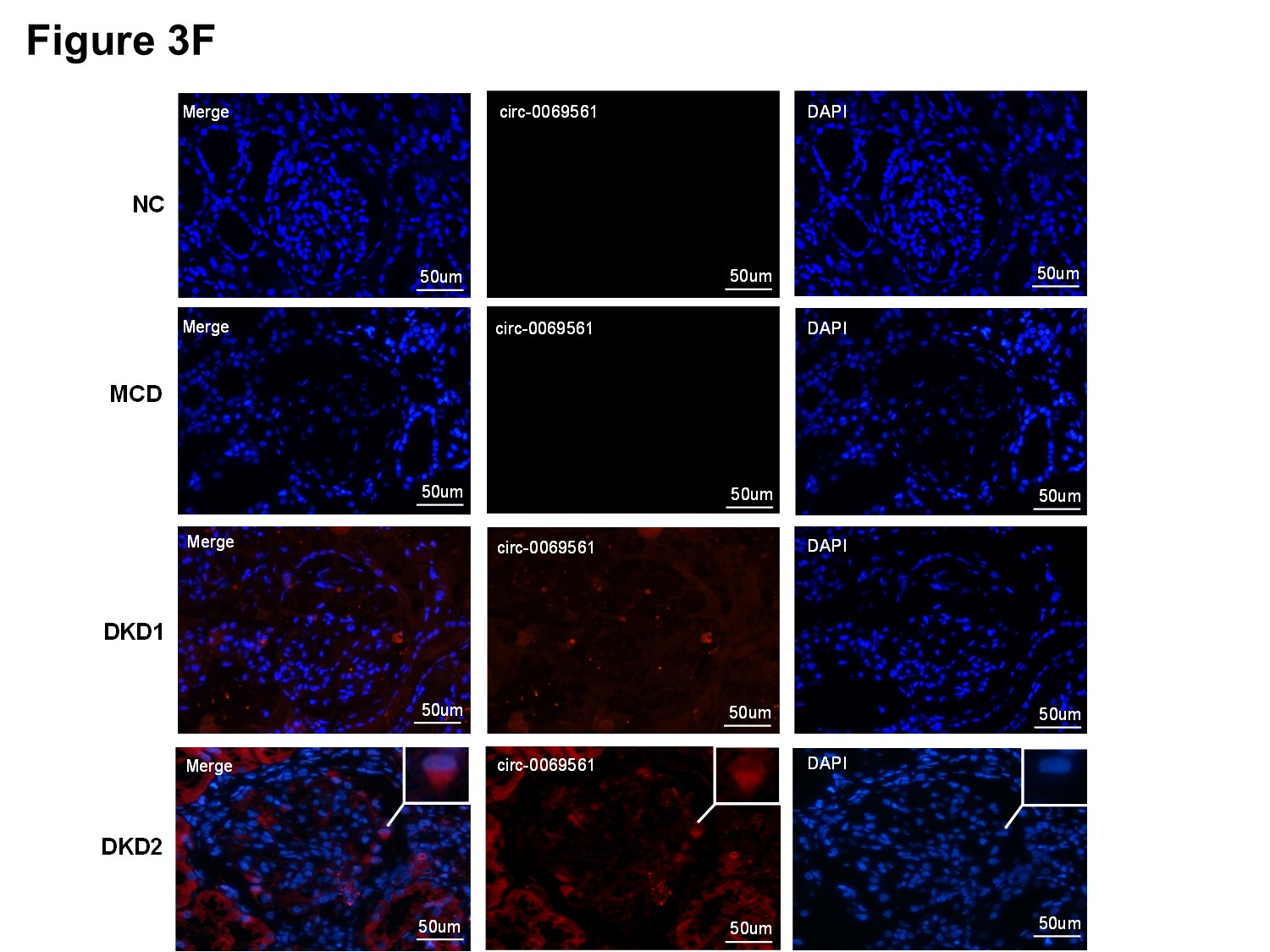

Supplement: Figure 3F.JPG [file IRNF_A_2490200_SM8340.jpg]

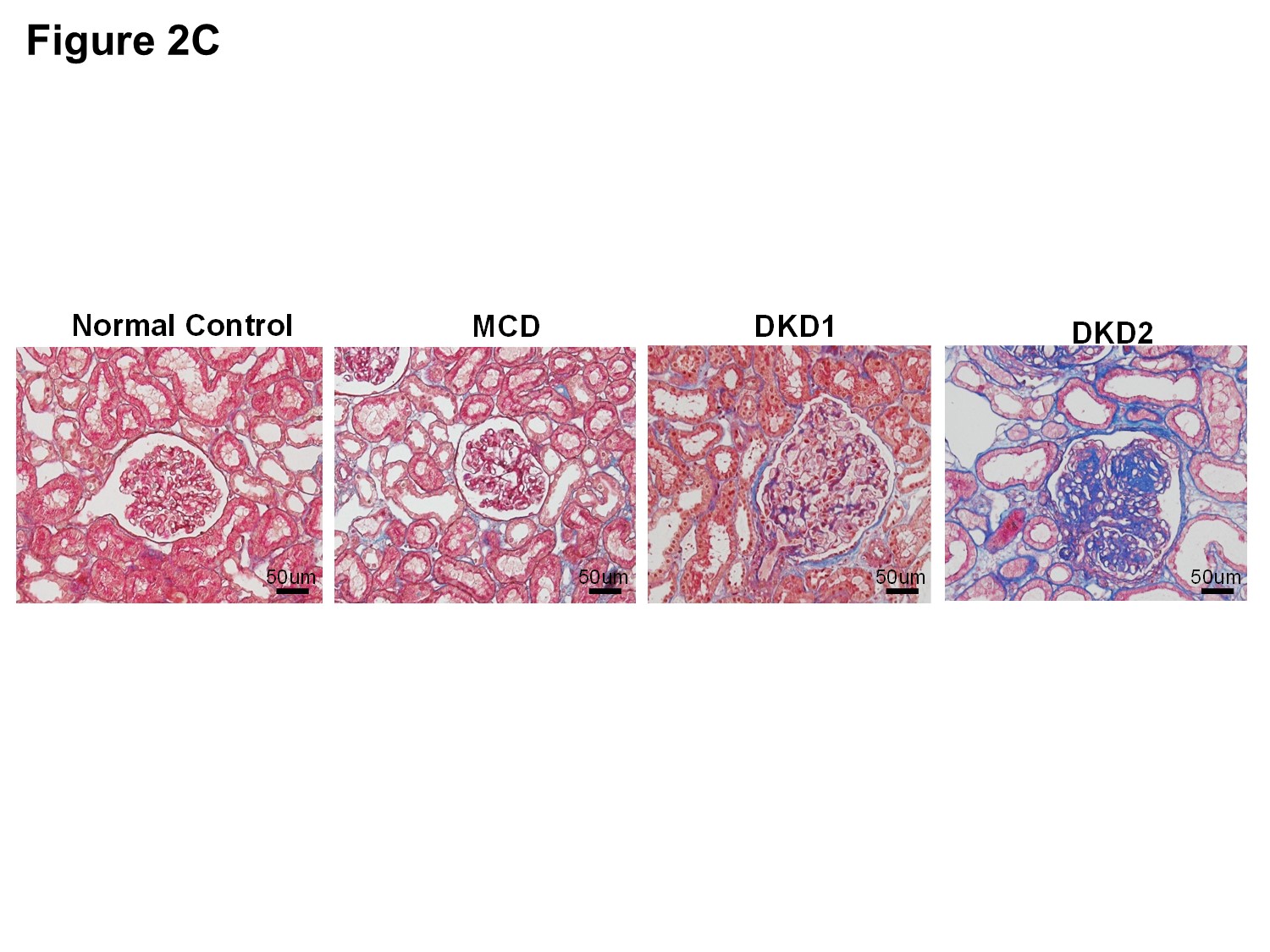

Supplement: Figure 2C.JPG [file IRNF_A_2490200_SM8339.jpg]

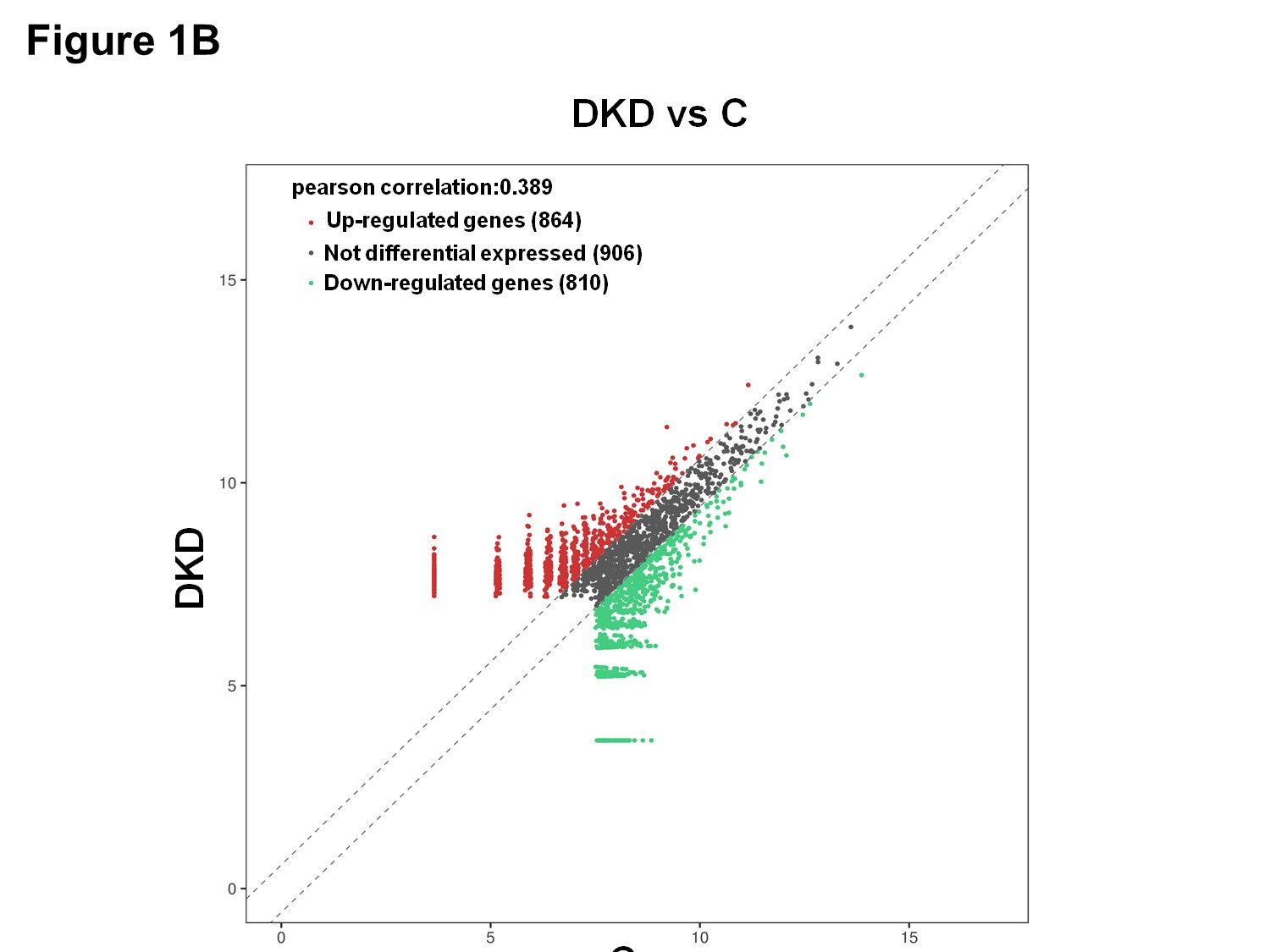

Supplement: Figure 1B.JPG [file IRNF_A_2490200_SM8338.jpg]

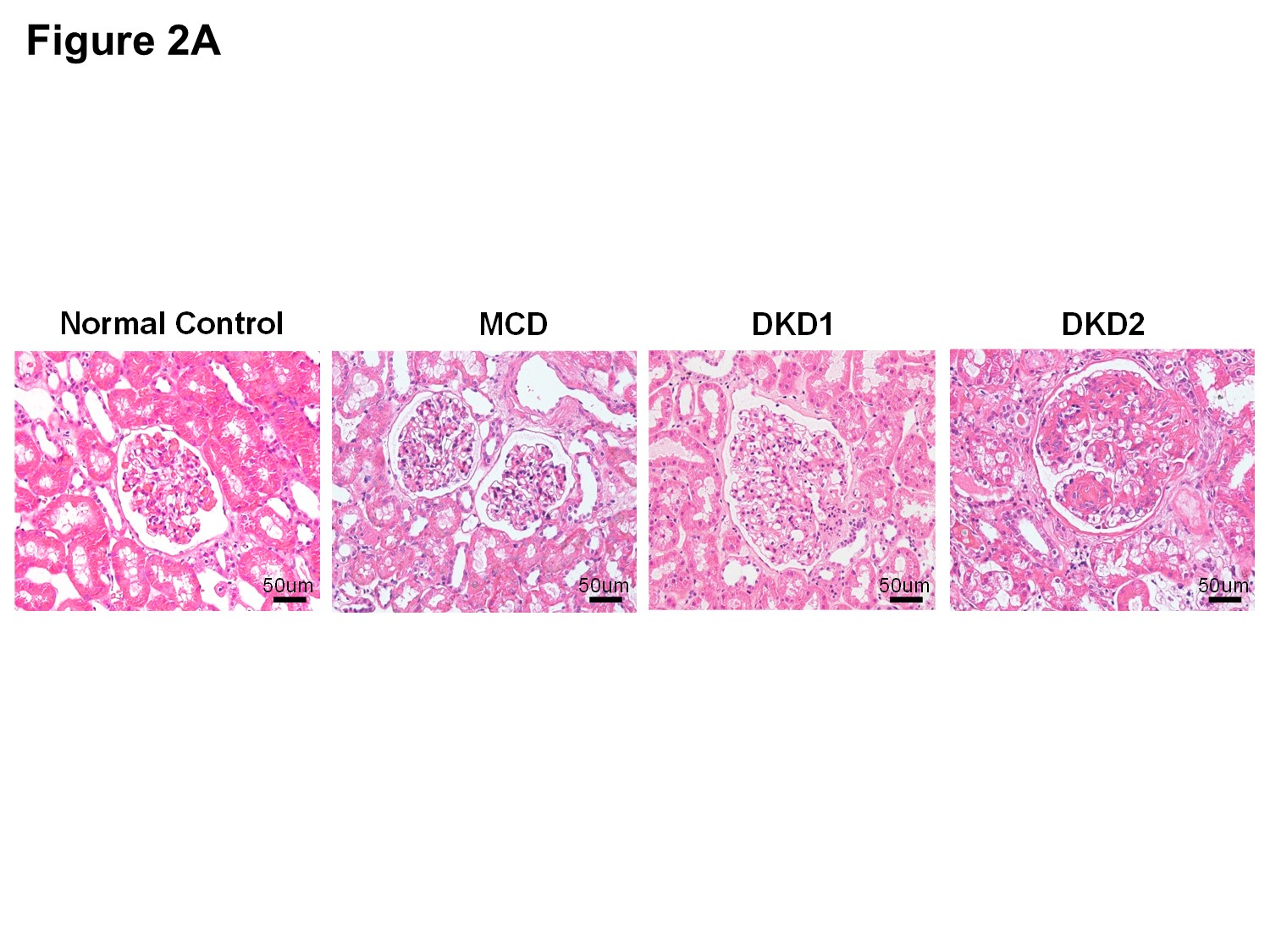

Supplement: Figure 2A.JPG [file IRNF_A_2490200_SM8337.jpg]

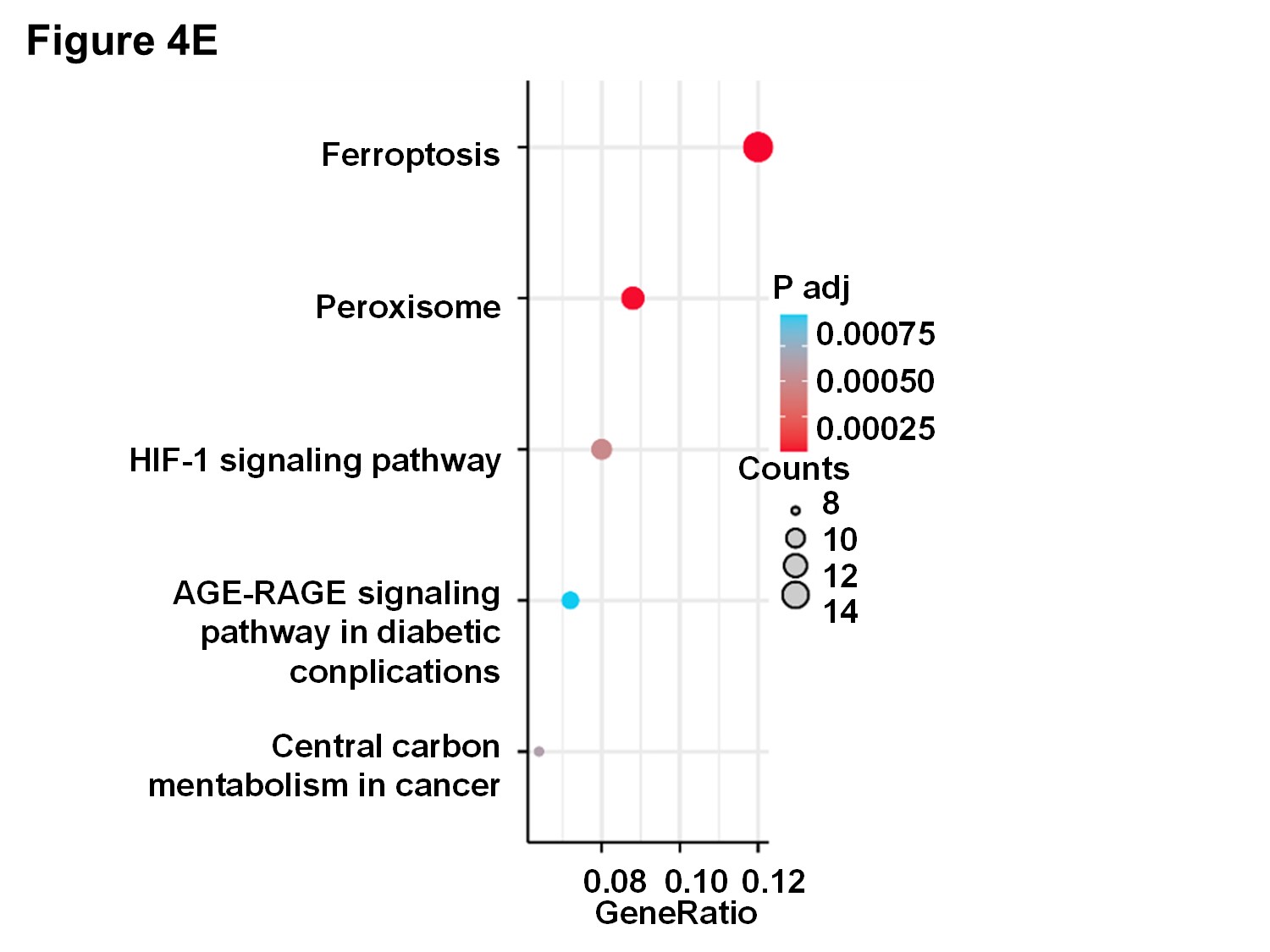

Supplement: Figure 4E.JPG [file IRNF_A_2490200_SM8336.jpg]

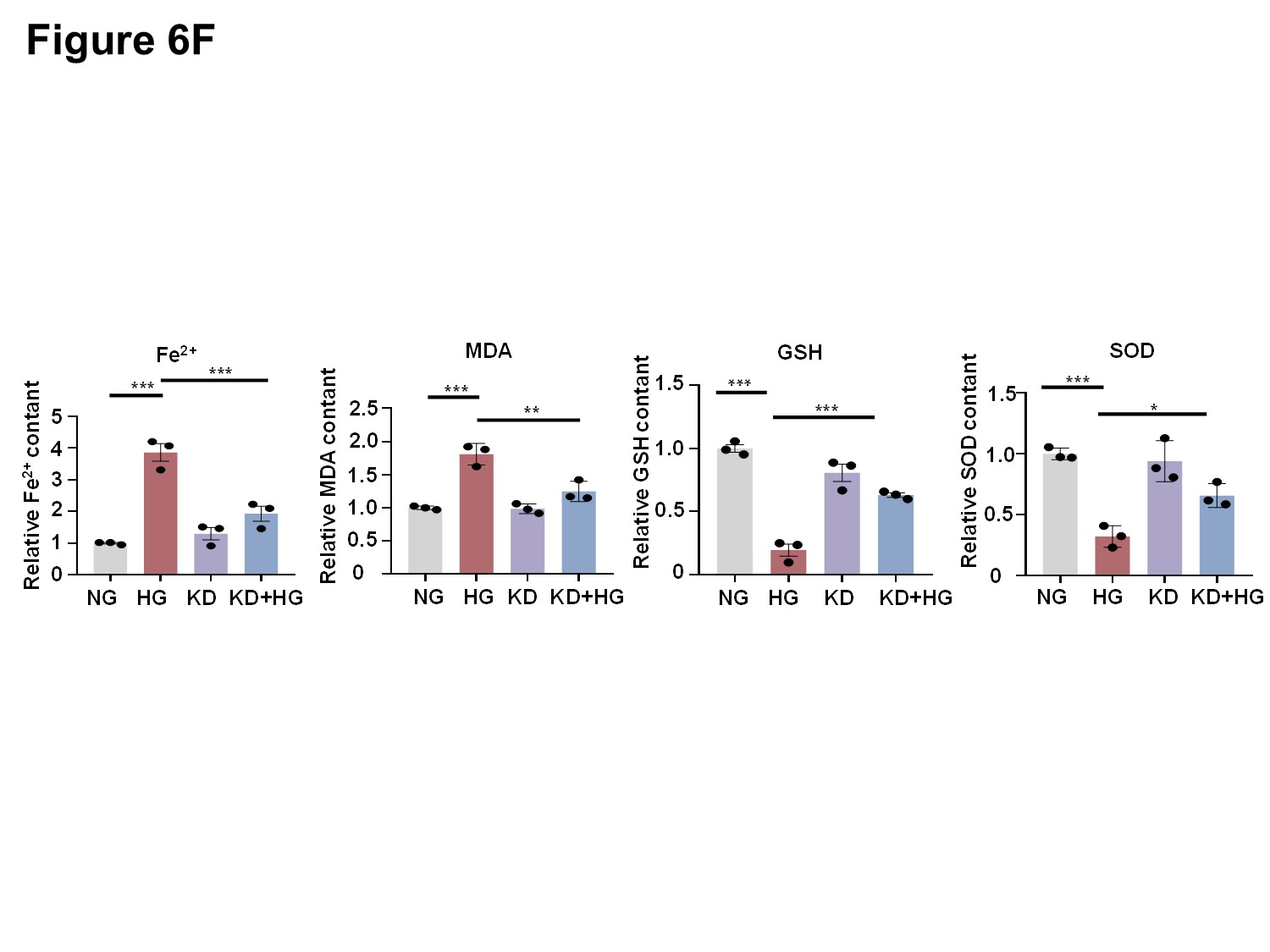

Supplement: Figure 6F.JPG [file IRNF_A_2490200_SM8335.jpg]

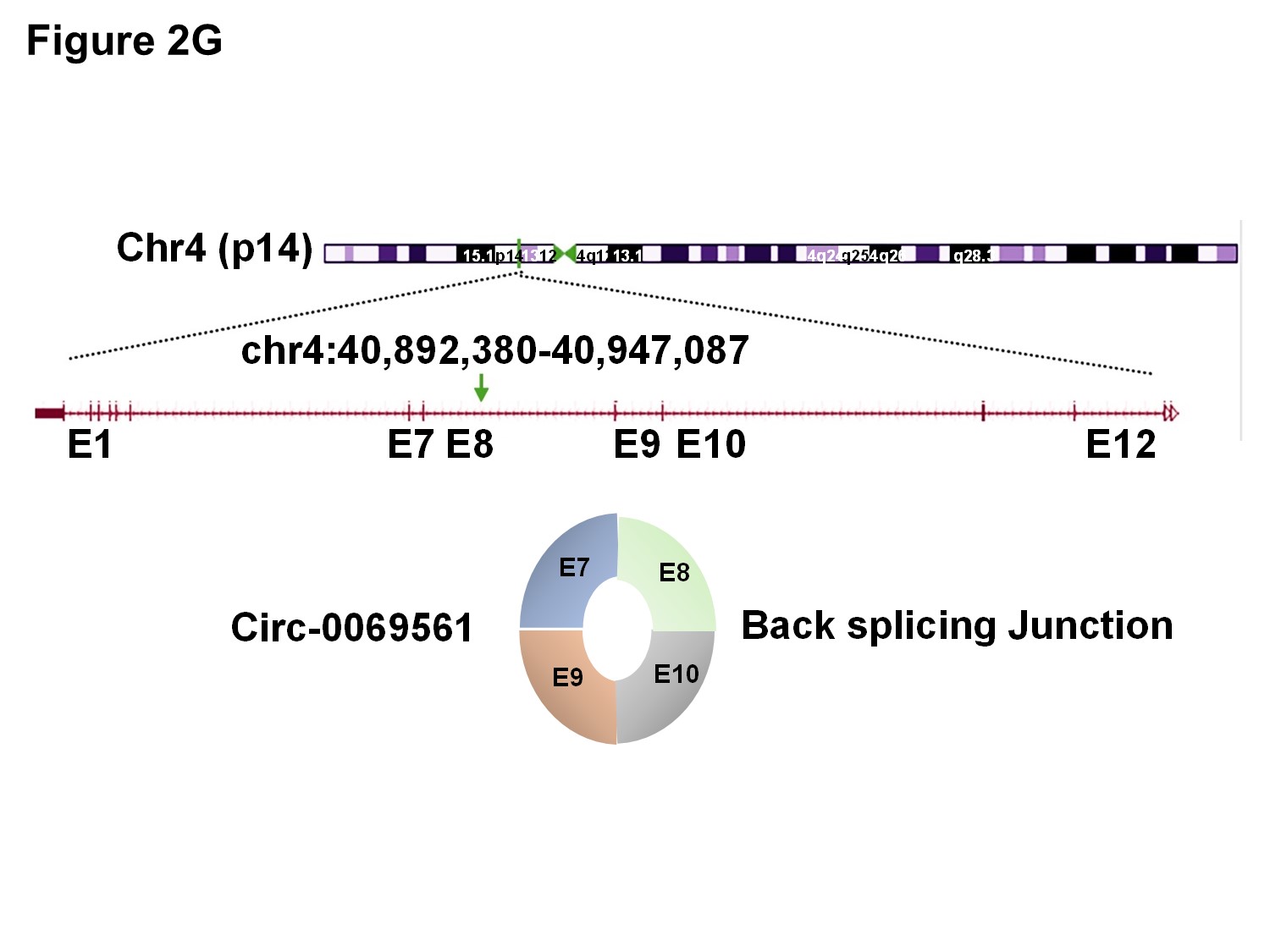

Supplement: Figure 2G.JPG [file IRNF_A_2490200_SM8334.jpg]

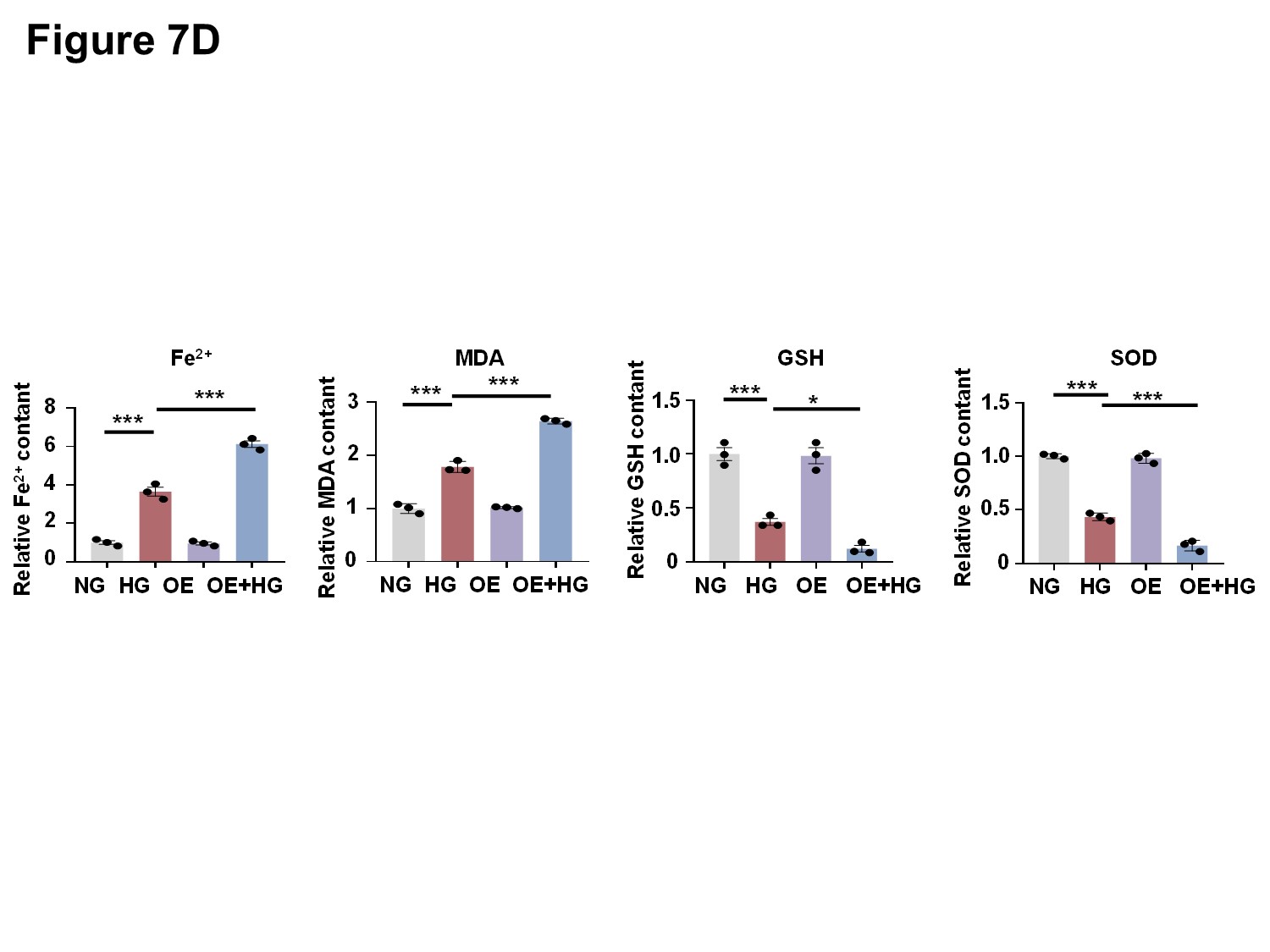

Supplement: Figure 7D.JPG [file IRNF_A_2490200_SM8333.jpg]

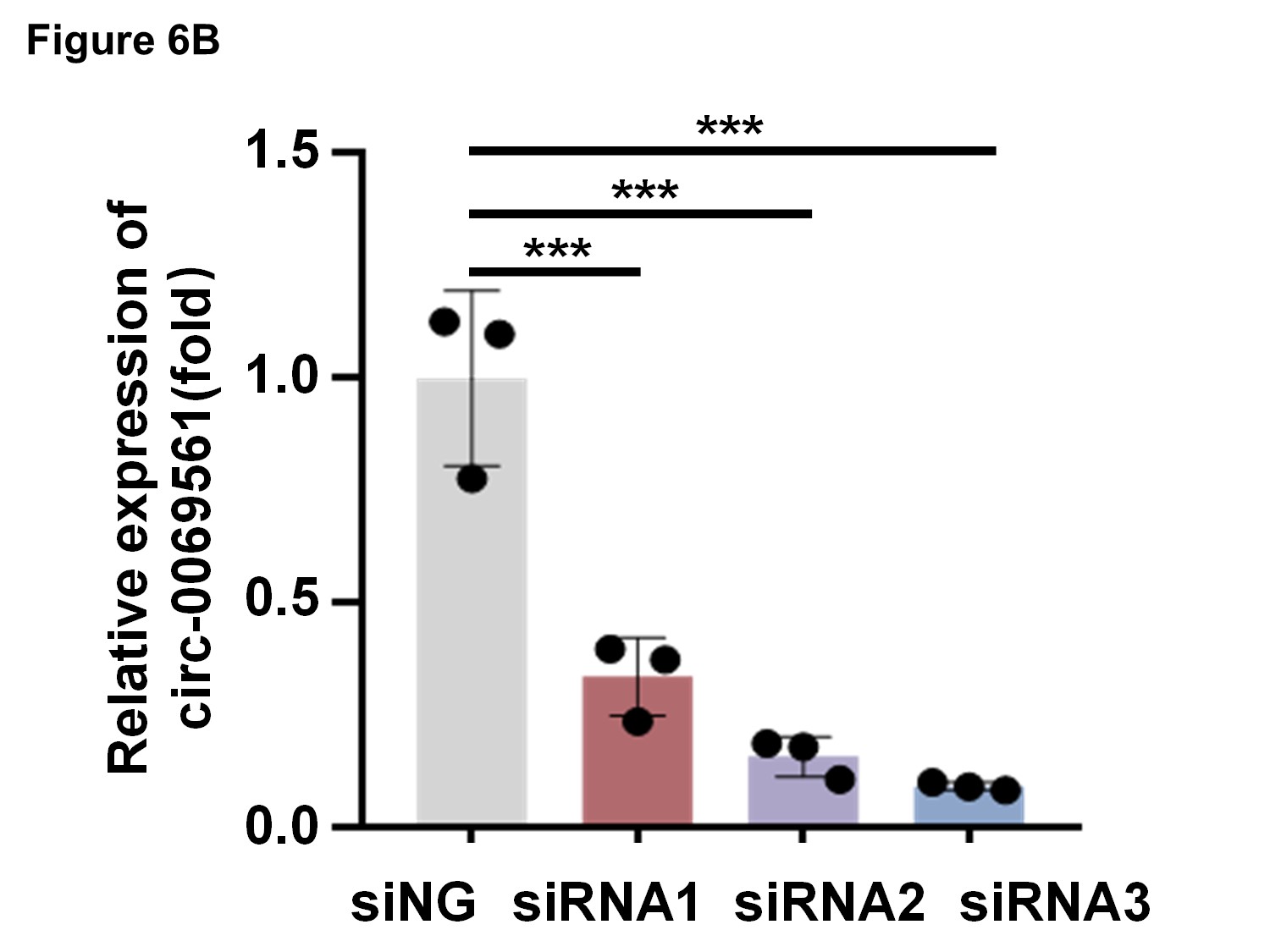

Supplement: Figure 6B.JPG [file IRNF_A_2490200_SM8332.jpg]

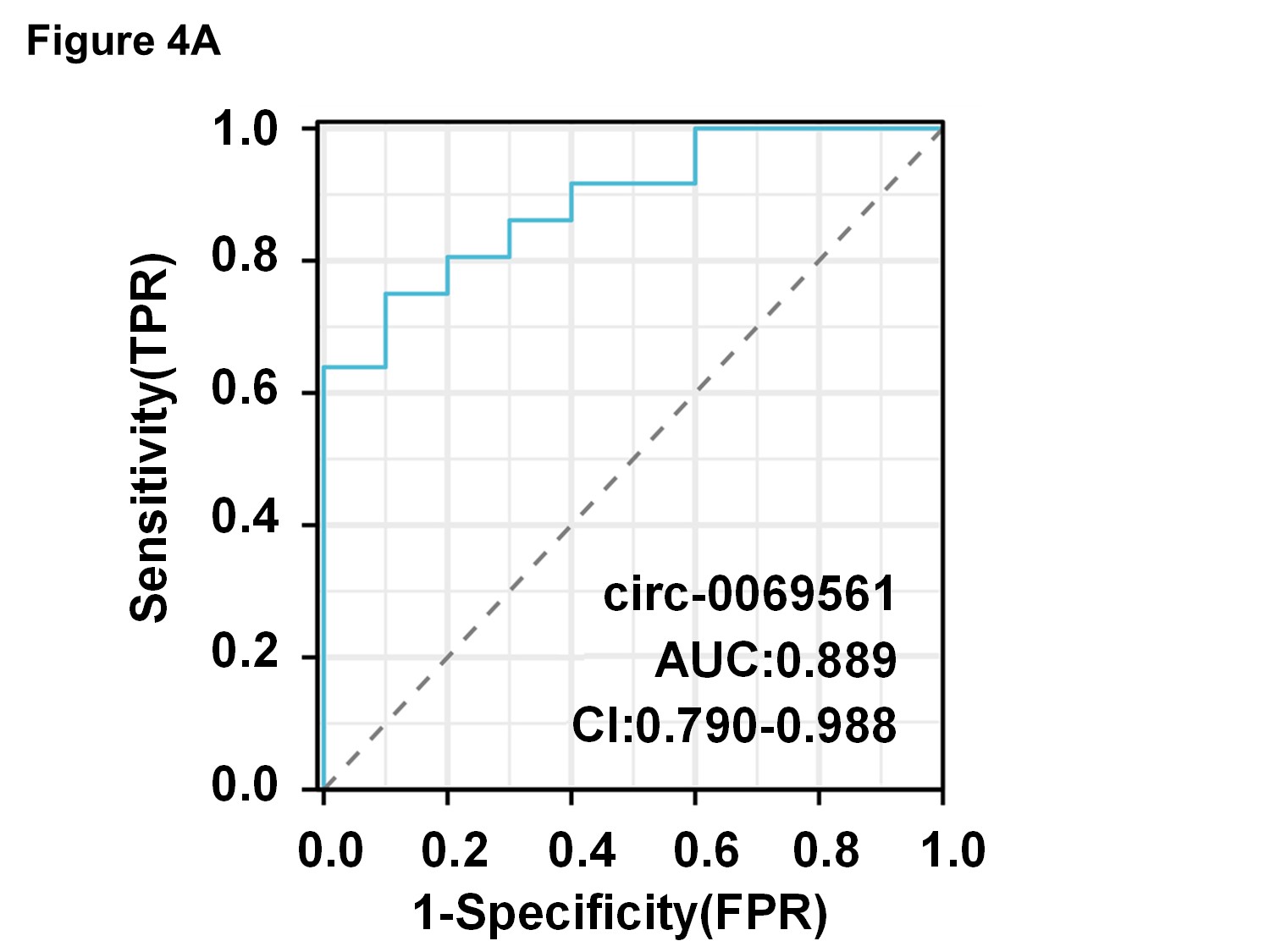

Supplement: Figure 4A.JPG [file IRNF_A_2490200_SM8330.jpg]

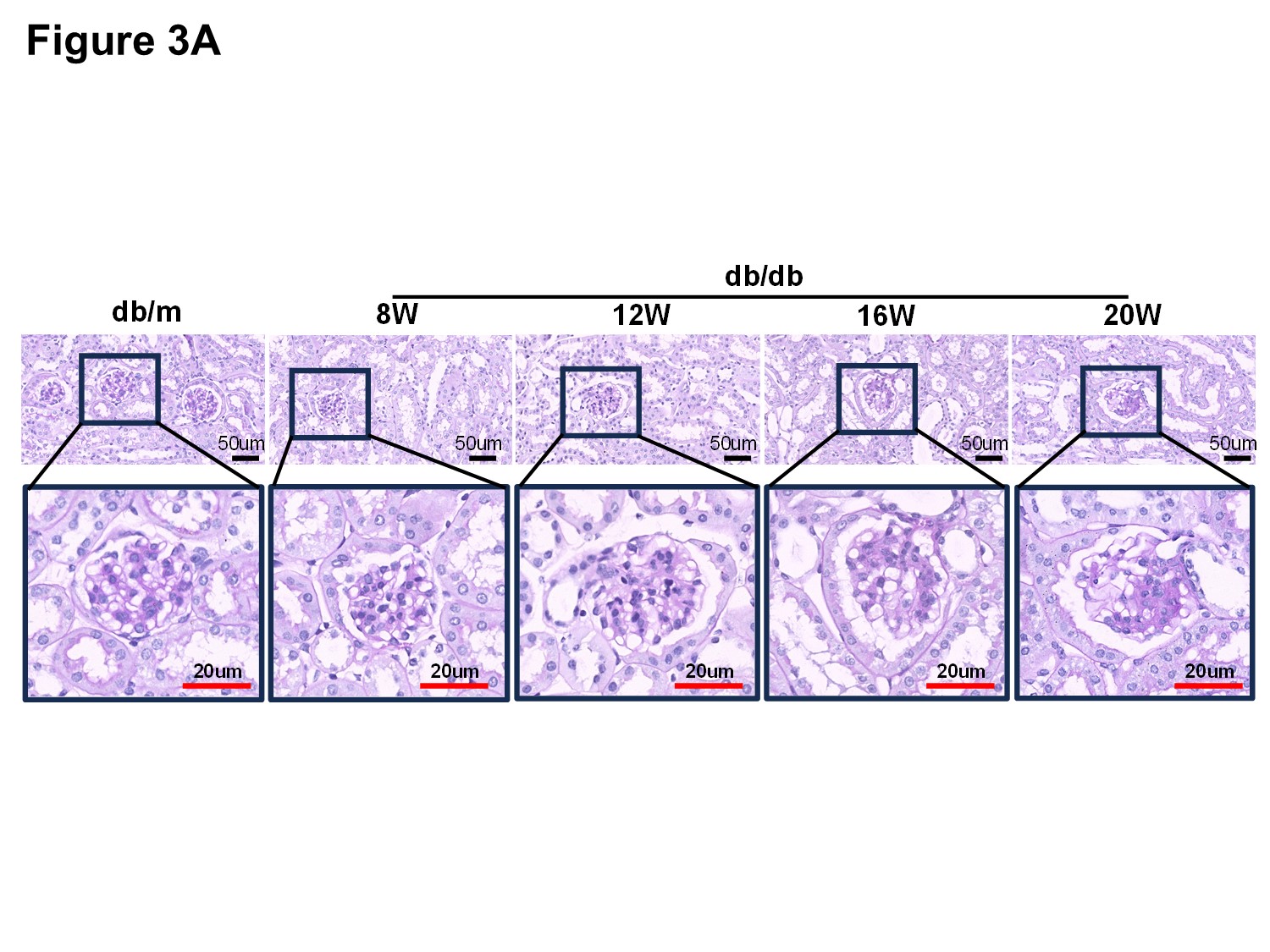

Supplement: Figure 3A.JPG [file IRNF_A_2490200_SM8329.jpg]

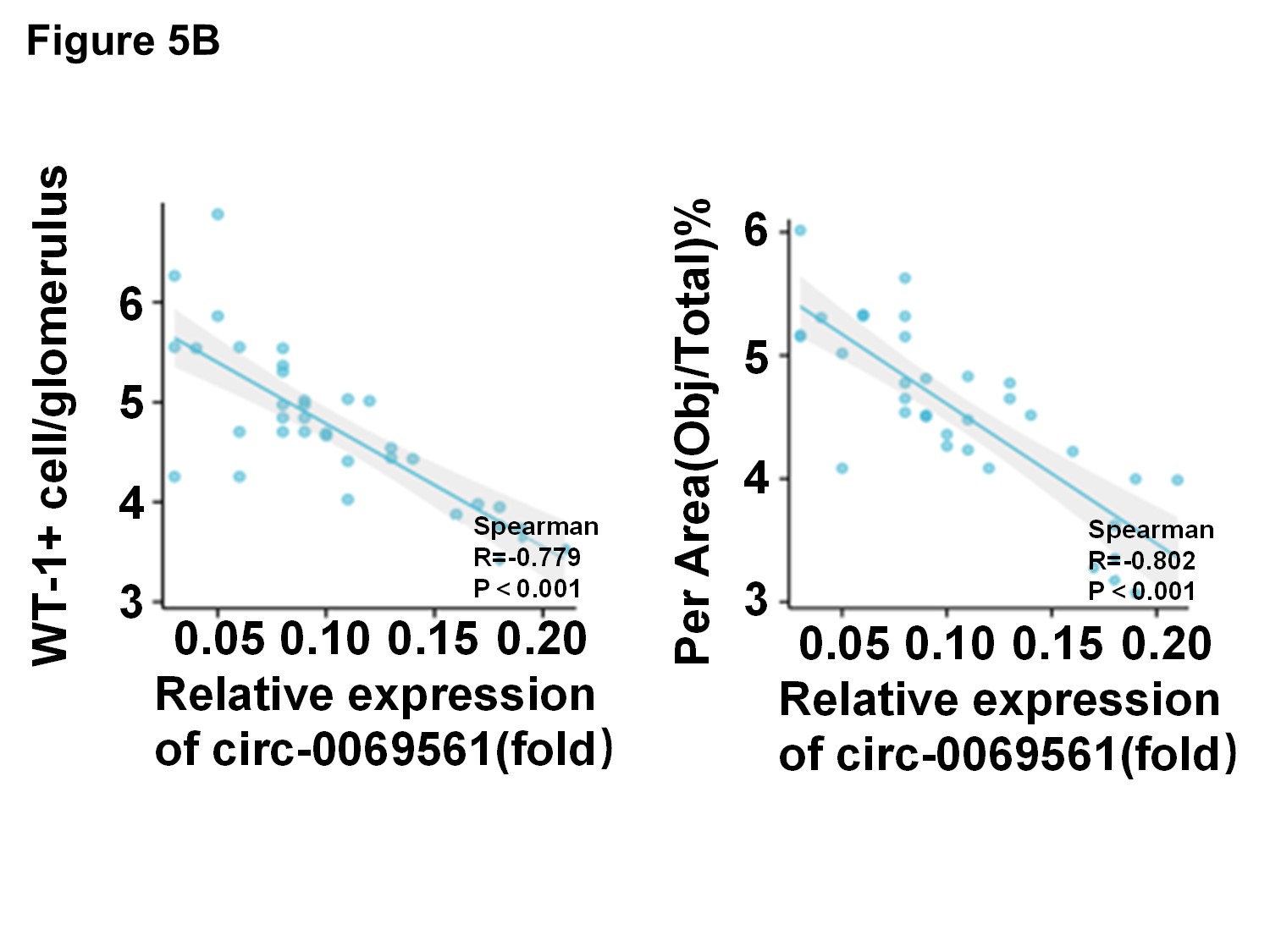

Supplement: Figure 5B.JPG [file IRNF_A_2490200_SM8328.jpg]

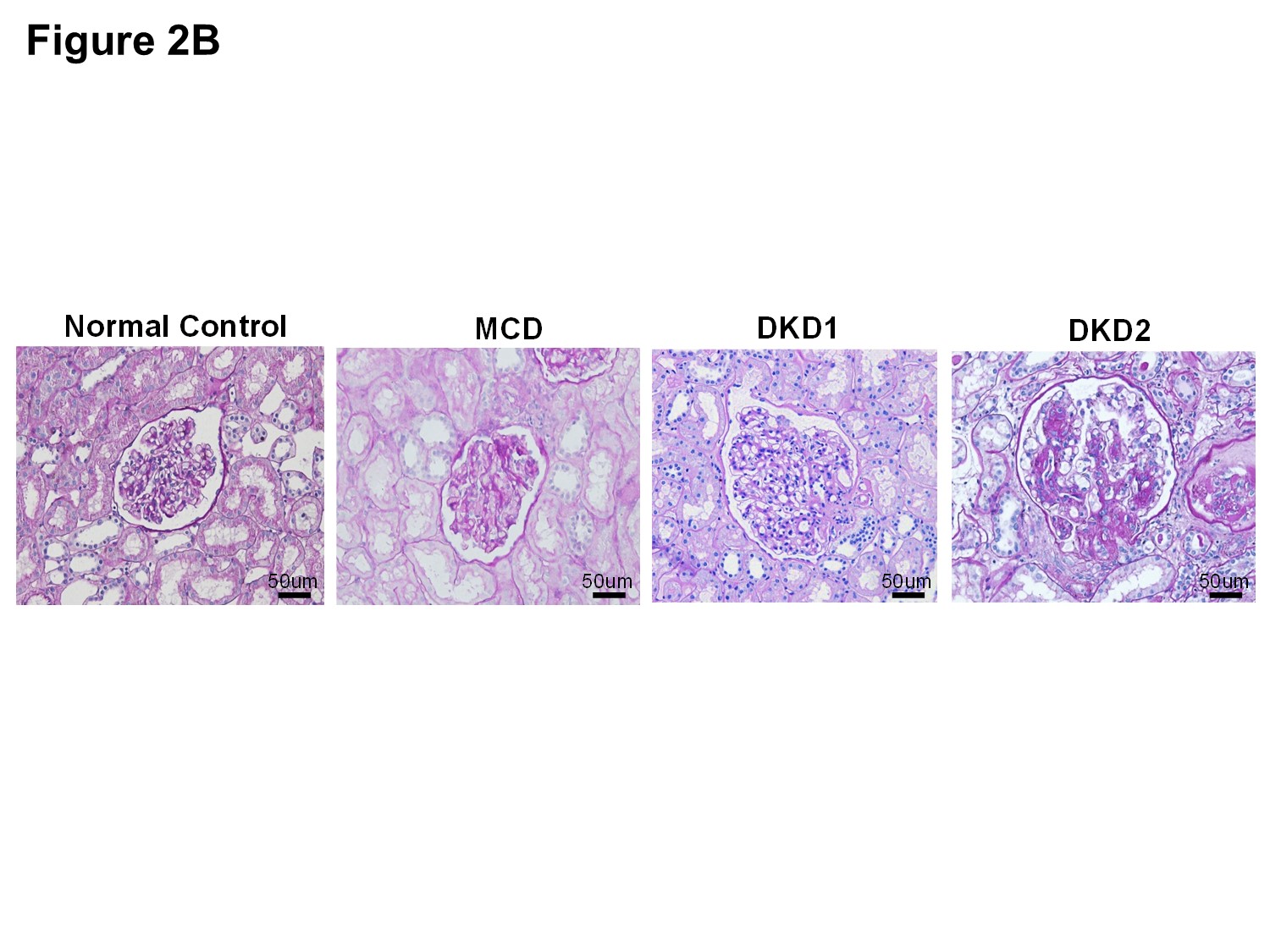

Supplement: Figure 2B.JPG [file IRNF_A_2490200_SM8327.jpg]

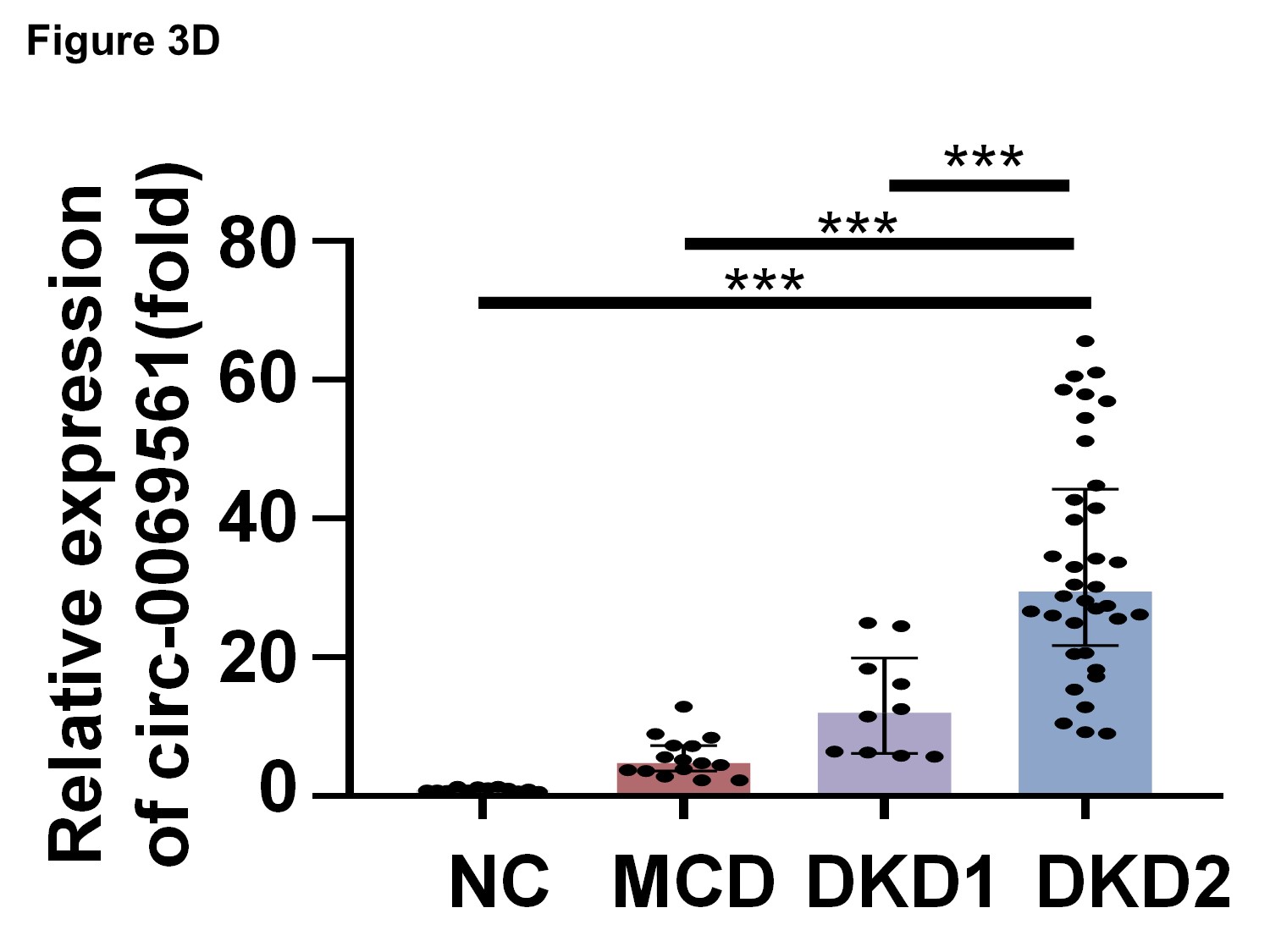

Supplement: Figure 3D.JPG [file IRNF_A_2490200_SM8326.jpg]

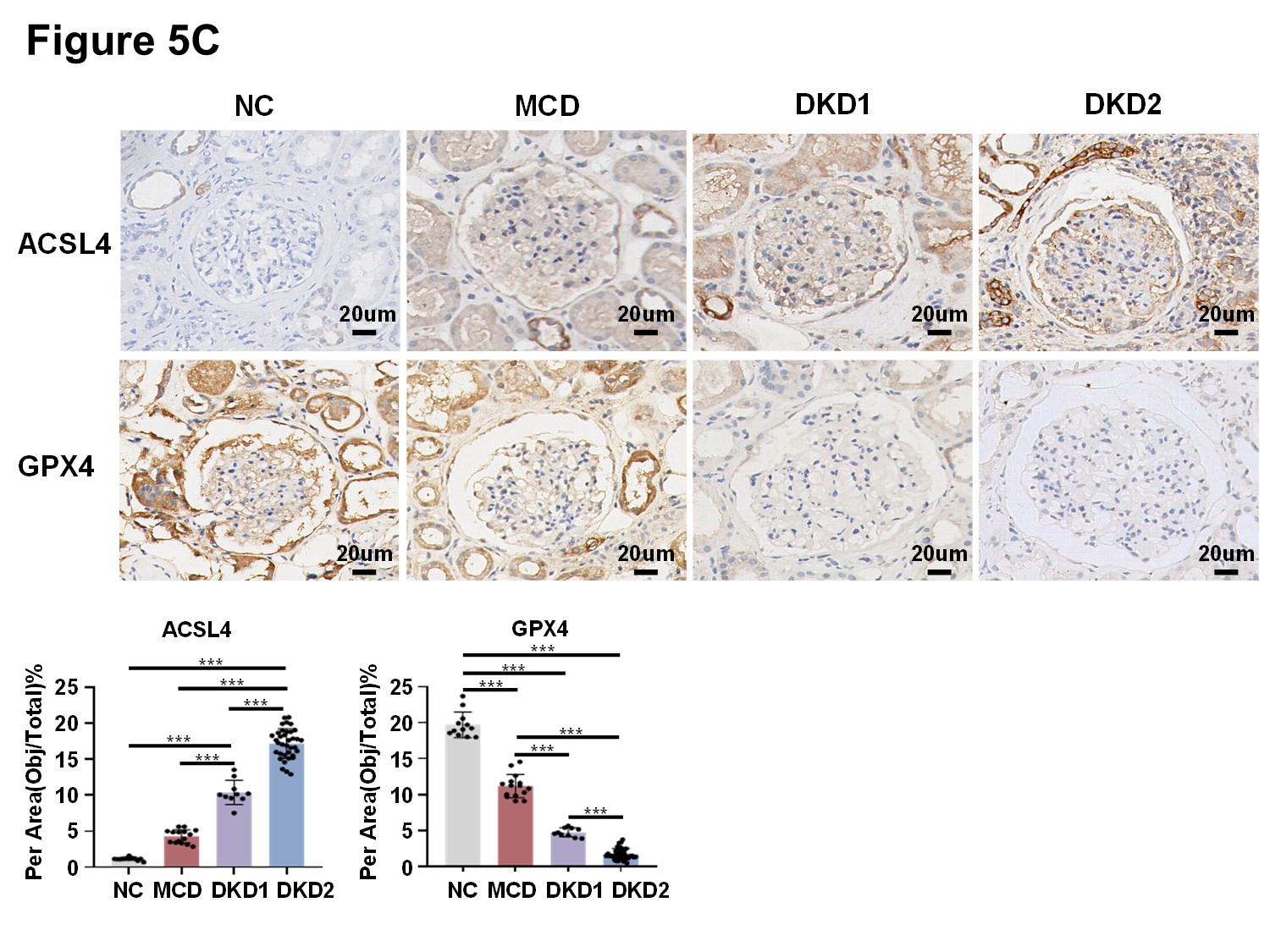

Supplement: Figure 5C.JPG [file IRNF_A_2490200_SM8325.jpg]

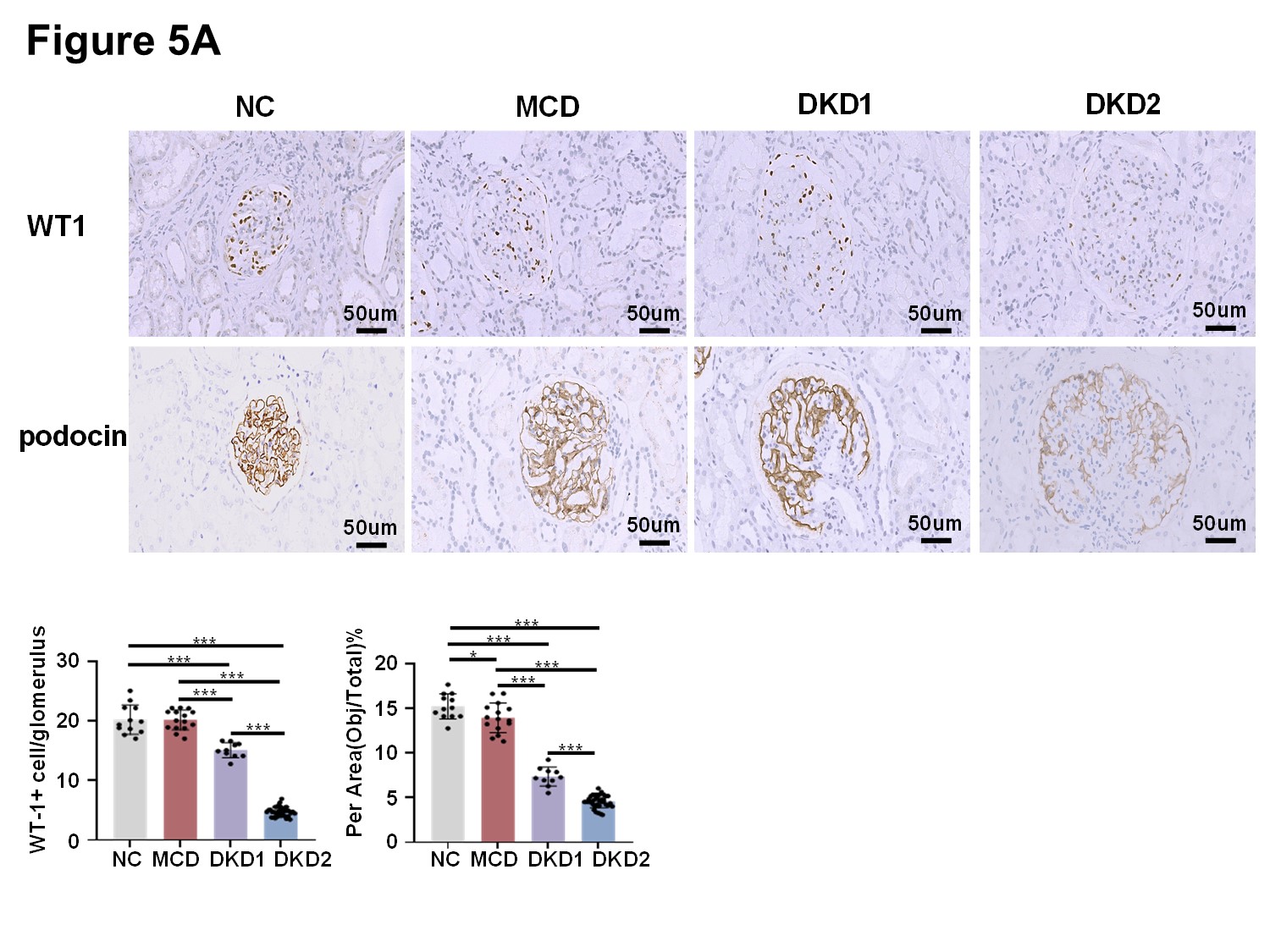

Supplement: Figure 5A.JPG [file IRNF_A_2490200_SM8323.jpg]

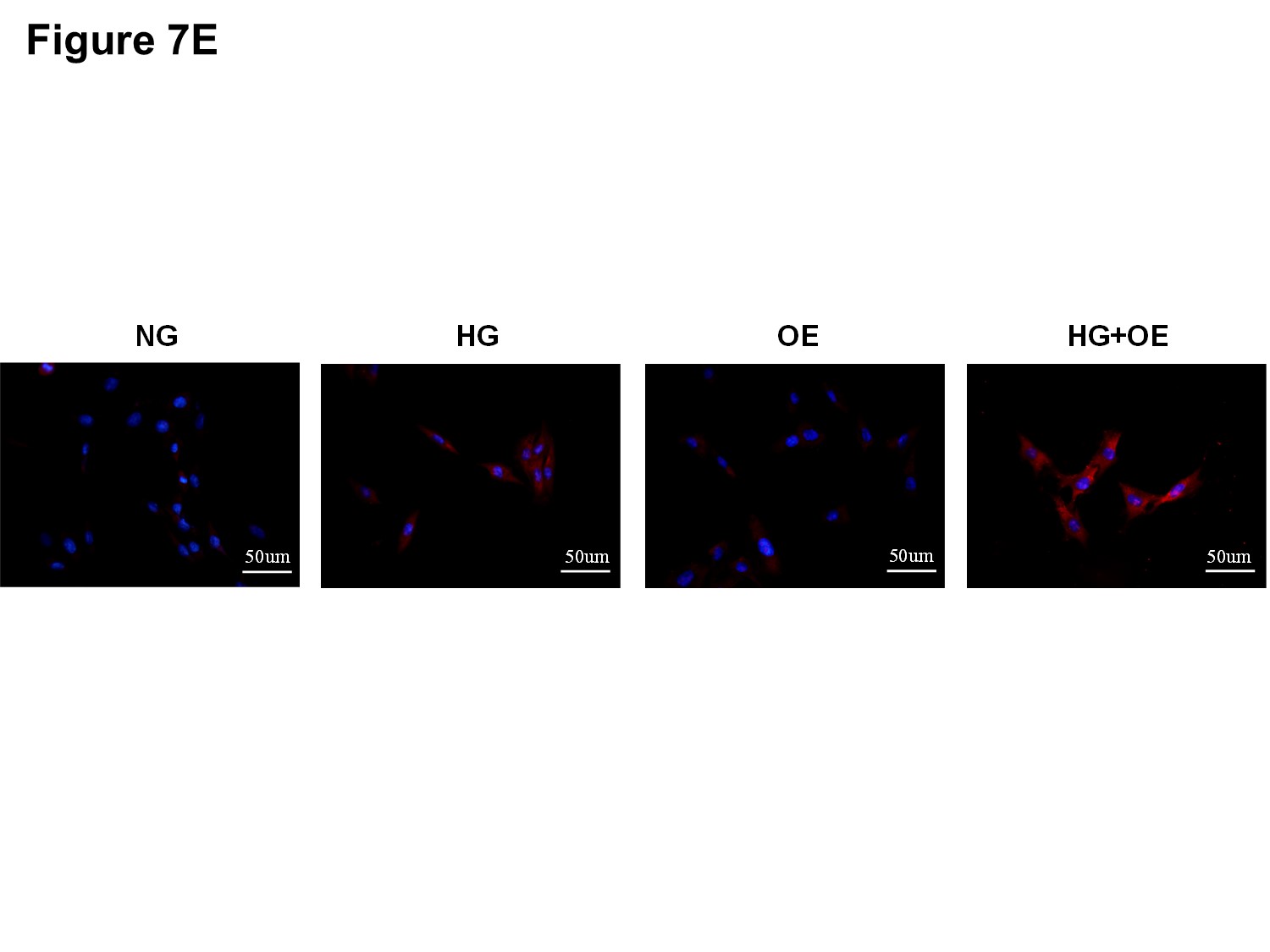

Supplement: Figure 7E.JPG [file IRNF_A_2490200_SM8322.jpg]
