# Supplementary material for: Circ-0069561 as a novel diagnostic biomarker for progression of diabetic kidney disease
Source: Ren Fail. 2025 Apr 22;47(1):2490200. doi: 10.1080/0886022X.2025.2490200 (PMC12016256; doi:10.1080/0886022X.2025.2490200)
Supplement: Figure legends.doc [file IRNF_A_2490200_SM8331.doc]

**Figure legends**

**Figure 1.** **Analysis of differentially expressed circRNA in DKD and NC renal tissues.** (A) Heatmap comparing the expression levels of circRNAs in the DKD group (n = 4) with the NC control group (n = 4). Horizontal axis markers represent DKD and NC samples, and vertical axis markers represent circRNAs expression levels. Expression values are indicated by the color scale. The intensity gradually increases from green (relatively low expression) to red (relatively high expression). Each column represents a tissue sample and each row represents a circRNAs. (B) Scatterplot to assess the distribution of circRNAs between DKD and NC groups. Red dots represent up-regulated circRNAs, green dots represent down-regulated circRNAs, and grey dots represent circRNAs that were not differentially expressed. (C) Length of circRNAs. (D) Reverse shear reads of circRNAs. (E) Volcano plots of differentially expressed circRNAs in the DKD and NC groups. Red dots indicate significantly differentially expressed upregulated circRNAs and green dots indicate significantly differentially expressed downregulated circRNAs. outside the two vertical lines are circRNAs with log2FC absolute value ≥ 0.585, and the horizontal line indicates a p-value of 0.05. (F) Percentage of new and known differentially expressed circRNAs. (G) Location of differentially expressed circRNAs on the position on human chromosomes.

**Figure 2. Screening for differentially expressed circRNAs.** (A) H&E staining of human kidney. Scale bar = 50 μm. (B) PAS staining of human kidney. Scale bar = 50 μm. (C) MASSON staining of human kidney. Scale bar = 50 μm. (D) PASM staining of human kidney. Scale bar = 50 μm. (E) TEC of human kidney. scale bar = 2 μm. (F) Relative expression levels of 8 up-regulated circRNAs in renal tissues of 46 DKD patients and 12 NC controls, **p* < 0.05, ***p* < 0.01, ****p* < 0.001. (G) Public prediction sites predicted the genomic structure of circ-0069561. (H) Sanger sequencing confirmed the circ-0069561 reverse splice site. H&E, haematoxylin and eosin; PAS, periodate-schiff; PASM, periodic acid-silver methenamine; TEM, transmission electron microscopy.

**Figure 3. Circ-0069561 expression is up-regulated in type 2 diabetic mice and DKD patients. (**A) PAS staining showing typical glomerular structure changes in different groups of mice. Scale bars: black 50 um; red 20 um. (B) Diabetic mice have significantly higher blood glucose, ***p < 0.001. (C) RT-PCR analysis revealed that circ-0069561 expression is up-regulated in renal tissues of db/db, ****p* < 0.001. (D) RT-PCR analysis demonstrated that circ-0069561 expression is up-regulated in patients with clinical DKD, ****p* < 0.001. (E) FISH experiment revealed that circ-0069561 expression is up-regulated in renal tissues of db/db. Scale bars: 20 um. (F) FISH experiment revealed that the expression level of circ-0069561 was significantly higher in kidney tissues of patients with clinical DKD than that of controls. Scale bars: 50 um.

**Figure 4.** **Function of mRNAs in the ceRNA network in DKD and construction of circRNA-miRNA-ferroptosis-related mRNA network.** (A) ROC curve analysis of circ-0069561 expression in DKD patients. **(**B) The Kaplan–Meier analysis for DKD patients with different levels of circ-0069561. **(**C) Wayne diagram showing intersection analysis of ferroptosis-associated mRNAs with differentially expressed mRNAs in the ceRNA network of circ-0069561. **(**D) CircRNA-miRNA-ferroptosis-associated mRNA network of circ-0069561. Red circles indicate miRNAs that may be more relevant to DKD. Blue circles represent mRNAs that may be involved in DKD pathogenesis. **(**E) Significant enrichment of the top 5 KEGG pathways. (F) Representative transmission electron micrographs of podocyte in control and db/db groups. Scale bar: 500 nm (n = 6). (G) Immunohistochemistry results showed that ACSL4 levels were increased and GPX4 levels were decreased in the db/db groups. Scale bar: 20 um (n = 6), ****p* < 0.001. (H) Western blot results also showed that ACSL4 levels were increased and GPX4 levels were decreased in the db/db groups. (n = 6), ****p* < 0.001.

**Figure 5. Significant correlation between circ-0069561 expression levels and levels of podocyte injury and ferroptosis in DKD group with massive proteinuria. (**A) Immunohistochemistry staining of glomerulus WT1 and podocin. Scale bar: 50 um, **p* < 0.05, ****p* < 0.001. **(**B) Correlation analysis between circ-0069561 expression level and levels of podocyte injury in DKD group with massive proteinuria. **(**C) Immunohistochemistry staining of glomerulus ACSL4 and GPX4. Scale bar: 20 um, ****p* < 0.001. **(**D) Correlation analysis between circ-0069561 expression level and levels of ferroptosis in DKD group with massive proteinuria.

**Figure 6. Silencing circRNA-0069561 attenuates high glucose-induced podocyte damage and ferroptosis. (**A) Real-time PCR showed that circ-0069561 expression was significantly elevated in high glucose-induced MPC5. mTEC: mouse tubular epithelial cells; vascular endothelial cells: VECs; MPC5: mouse podocyte clone 5; SV40-MES13: mouse glomerulus mesangial cells, **p* < 0.05, ****p* < 0.001. **(**B) Real-time PCR screening of circ-0069561 siRNA sequences for the best knockdown effect, ****p* < 0.001. **(**C) The expression level of circ-0069561 was verified by FISH experiment. Scale: 10 μm, ****p* < 0.001. **(**D) Western blot and quantification showed that silencing circ-0069561 attenuated high glucose-induced loss of the podocyte marker proteins WT1 and podocin, **p* < 0.05, ***p* < 0.01, ****p* < 0.001. **(**E) Western blot and quantification showed that silencing circ-0069561 attenuated high glucose-induced ferroptosis levels in podocytes, **p* < 0.05, ****p* < 0.001. **(**F) Fe^2+^, MDA, GSH and SOD level in each group, **p* < 0.05, ***p* < 0.01, ****p* < 0.001. **(**G) Fluorescent probe DHE staining to detect ROS levels in each group of MPC5 cells. Scale bar: 50 um.

**Figure 7. Overexpression of circRNA-0069561 aggravates high glucose-induced podocyte damage and ferroptosis. (**A) The overexpression of circ-0069561 was verified by Real-time PCR, ****p* < 0.001. **(**B) Western blot and quantitative analysis showed that overexpression of circ-0069561 aggravated high glucose-induced loss of the podocyte marker proteins WT1 and podocin, ****p* < 0.001. **(**C) Western blot and quantification showed that overexpression of circ-0069561 aggravated high glucose-induced ferroptosis levels in podocytes, ***p* < 0.01, ****p* < 0.001. **(**D) Fe^2+^, MDA, GSH and SOD level in each group, **p* < 0.05, ****p* < 0.001. **(**E) Fluorescent probe DHE staining to detect ROS levels in each group of MPC5 cells. Scale bar: 50 um.
