# Supplementary material for: Circ-0069561 as a novel diagnostic biomarker for progression of diabetic kidney disease
Source: Ren Fail. 2025 Apr 22;47(1):2490200. doi: 10.1080/0886022X.2025.2490200 (PMC12016256; doi:10.1080/0886022X.2025.2490200)
Supplement: Supplementary Tables.doc [file IRNF_A_2490200_SM8324.doc]

| **Table S1** SiRNA Sequences | |  |
| --- | --- | --- |
| SiRNA | Sense sequences (5′−3′) | Antisense sequences (5′−3′) |
| NC | UUCUCCGAACGUGUCACGUTT | ACGUGACACGUUCGGAGAATT |
| circ-0069561-si1 | AGACAACGGCCGAUAUCUGGATT | UCCAGAUAUCGGCCGUUGUCUTT |
| circ-0069561-si2 | ACGGCCGAUAUCUGGAGUGAUTT | AUCACUCCAGAUAUCGGCCGUTT |
| circ-0069561-si3 | CCGAGACAACGGCCGAUAUCUTT | AGAUAUCGGCCGUUGUCUCGGTT |

| **Table S2** FISH probe sequences | |
| --- | --- |
| FISH Probes | Sequences (5′−3′) |
| mmu-circ-0069561 | ACTCCAGATATCGGCCGTTGTCTCG |
| has-circ-0069561 | CATATATCGGCCATTGTCGCGG |

| **Table S3** Primer sequences of RT-PCR | | |
| --- | --- | --- |
| Terms | Forward primer (5’-3’) | Reverse primer (5’-3’) |
| human |  |  |
| β-actin | CACCATTGGCAATGAGCGGTTC | AGGTCTTTGCGGATGTCCACGT |
| circ-0001195 | GACAGAGGAATTGGCAGTCTTGAT | CCAGTTGCTGGATCTATAAATGCT |
| circ-0069561 | CGCGACAATGGCCGATATATG | CCGGCAATGTCACTGACTCTT |
| circ-0022997 | TCATCCACACCCCGATGAG | GCCGGAACTCAACATCAAACTG |
| circ-0004069 | CTTCAGTATGGTTATGAGGTGTTTG | TTCACAATCCACAGGCAGCAG |
| circ-0130985 | GGAAGCACTTGCTAGGGAACC | GGTTCTTTCGGAAGTTCTGCC |
| circ-0008998 | CAGCTAGCACTGGACAGGTTCA | TGTATAGCCTGGTAAAGGTACAGCA |
| circ-0072780 | CTTCAGCTCAAACAAGAGGAGGT | CTCTGAGTTGTCTTTGAGATGCC |
| circ-0005857 | GAACCATTTGCAGAAGGAGCAG | GCAGTGTCTGTCTGCAGTGGT |
| mouse |  |  |
| β-actin | CATTGCTGACAGGATGCAGAA | ATGGTGCTAGGAGCCAGAGC |
| circ-0069561 | ATCAACAGTGACCCAGAAGCC | GTTCCCATTGAGTCGTCCCT |

| **Table S4** Top 10 up-regulated circRNAs | | | |
| --- | --- | --- | --- |
| CircRNA_ID | Genomic_Length | log2FC | *P* value |
| chr1:245180543-245180628:+ | 85.000 | 5.013 | 0.008 |
| circ-0001195 | 937.000 | 4.732 | 0.017 |
| circ-0069561 | 54707.000 | 4.585 | 0.033 |
| circ-0057338 | 6890.000 | 4.579 | 0.036 |
| circ-0022997 | 2613.000 | 4.542 | 0.036 |
| circ-0004069 | 3118.000 | 4.497 | 0.036 |
| circ-0130985 | 5678.000 | 4.474 | 0.046 |
| circ-0008998 | 544.000 | 4.418 | 0.048 |
| circ-0072780 | 16412.000 | 3.463 | 0.025 |
| circ-0005857 | 14067.000 | 3.341 | 0.036 |

| **Table S5** ROC curve analysis. | | | | | | | |
| --- | --- | --- | --- | --- | --- | --- | --- |
|  | Cut-off | AUC | 95% CI | *P* | Sensitivity | Specificity | Youden index |
| hsa_circ_0069561 | 0.078 | 0.889 | 0.790–0.988 | < 0.001 | 0.750 | 0.900 | 0.650 |

| **Table S6** Significant terms for the KEGG enrichment analysis | | | | |
| --- | --- | --- | --- | --- |
| Terms | Count | *P* value | *P* adjust | qvalue |
| Ferroptosis | 15 | 1.15E-17 | 2.90E-15 | 2.05E-15 |
| Peroxisome | 11 | 3.89E-08 | 4.90E-06 | 3.48E-06 |
| HIF-1 signaling pathway | 10 | 5.85E-06 | 0.00049 | 0.000348864 |
| Central carbon metabolism in cancer | 8 | 1.03E-05 | 0.00064 | 0.000460247 |
| AGE-RAGE signaling pathway in diabetic complications | 9 | 2.04E-05 | 0.00097 | 0.000696322 |
